# Microglia coordinate cellular interactions during spinal cord repair in mice

**DOI:** 10.1038/s41467-022-31797-0

**Published:** 2022-07-14

**Authors:** Faith H. Brennan, Yang Li, Cankun Wang, Anjun Ma, Qi Guo, Yi Li, Nicole Pukos, Warren A. Campbell, Kristina G. Witcher, Zhen Guan, Kristina A. Kigerl, Jodie C. E. Hall, Jonathan P. Godbout, Andy J. Fischer, Dana M. McTigue, Zhigang He, Qin Ma, Phillip G. Popovich

**Affiliations:** 1 Department of Neuroscience, The Ohio State University Wexner Medical Center, Columbus, OH 43210 USA; 2 Belford Center for Spinal Cord Injury, Center for Brain and Spinal Cord Repair, The Ohio State University Wexner Medical Center, Columbus, OH 43210 USA; 3 Department of Biomedical Informatics, The Ohio State University Wexner Medical Center, Columbus, OH 43210 USA; 4 F.M. Kirby Neurobiology Center, Boston Children’s Hospital, Department of Neurology, Harvard Medical School, Boston, MA USA; 5 Institute for Behavioral Medicine Research, The Ohio State University Wexner Medical Center, Columbus, OH 43210 USA; 6 Present Address: Institute of Neuroscience, Center for Excellence in Brain Science and Intelligence Technology, Chinese Academy of Sciences, Shanghai, China

**Keywords:** Microglia, Neuroimmunology

## Abstract

Traumatic spinal cord injury (SCI) triggers a neuro-inflammatory response dominated by tissue-resident microglia and monocyte derived macrophages (MDMs). Since activated microglia and MDMs are morphologically identical and express similar phenotypic markers in vivo, identifying injury responses specifically coordinated by microglia has historically been challenging. Here, we pharmacologically depleted microglia and use anatomical, histopathological, tract tracing, bulk and single cell RNA sequencing to reveal the cellular and molecular responses to SCI controlled by microglia. We show that microglia are vital for SCI recovery and coordinate injury responses in CNS-resident glia and infiltrating leukocytes. Depleting microglia exacerbates tissue damage and worsens functional recovery. Conversely, restoring select microglia-dependent signaling axes, identified through sequencing data, in microglia depleted mice prevents secondary damage and promotes recovery. Additional bioinformatics analyses reveal that optimal repair after SCI might be achieved by co-opting key ligand-receptor interactions between microglia, astrocytes and MDMs.

## Abstract (web summary)

Here the authors show, using microglia-specific depletion techniques and single cell transcriptomics, that optimal repair after murine spinal cord injury (SCI) requires microglia.

## Introduction

Microglia are central nervous system (CNS)-resident macrophages and comprise ~10% of all CNS cells^1–3^. Microglia help maintains CNS homeostasis via constant interaction with both neuronal and nonneuronal cells^4,5^. In clinical and experimental CNS lesions, including spinal cord injury (SCI)^6–9^, microglia together with monocyte-derived macrophages (MDMs), are the predominant cell types initiating neuroinflammatory reactions and both cell types can repair damaged CNS tissue or cause bystander (secondary) injury to neurons, glia and endothelia^10–14^. Since microglia and MDMs adopt similar morphologies and share phenotypic markers in the pathological CNS, it has been difficult to ascribe distinct roles for either cell type in vivo. However, with the advent of new research tools including microglia-specific depletion techniques and advanced sequencing technologies, it is now possible to study how microglia control diverse cellular and molecular responses after SCI. Here, we apply these tools in three different SCI models and show that optimal repair after SCI and likely other forms of neurological disease, require microglia.

## Results

### Microglia are required to achieve optimal recovery of function and restore lesion homeostasis after SCI

**Fig. 1:**
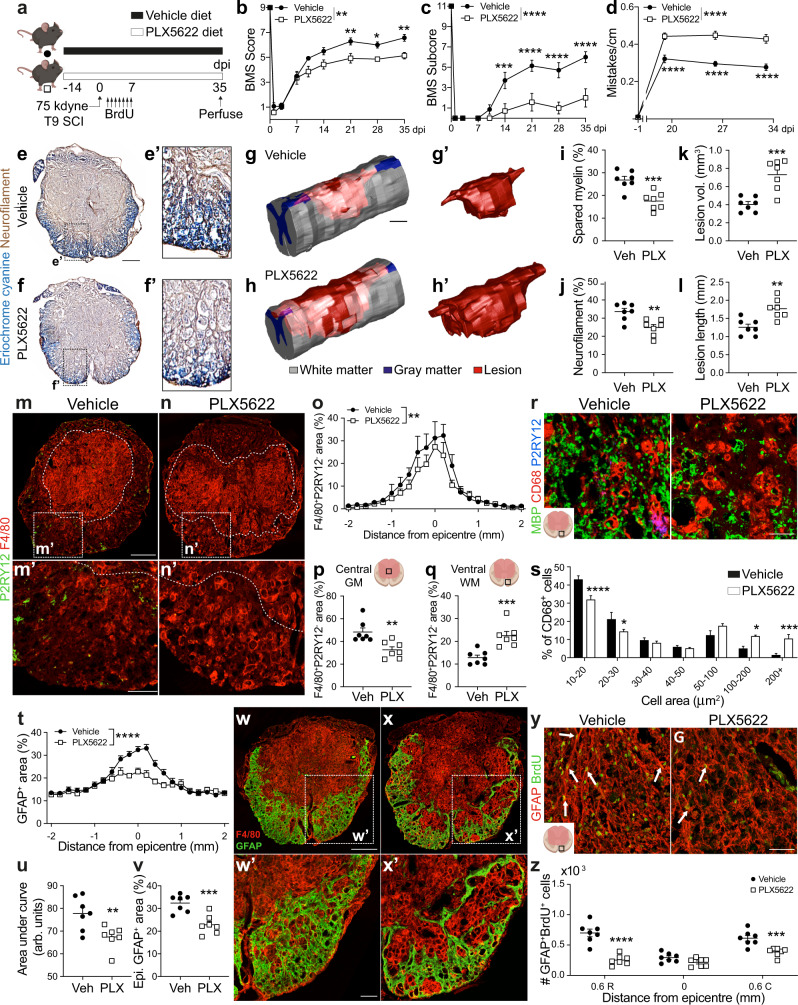
Microglia depletion worsens motor recovery and increases tissue pathology after 75 kdyne T9 contusion SCI. **a** Experimental timeline. **b**–**d** BMS scores (**b**), BMS subscores (**c**) and horizontal ladder (**d**) data. **e**, **f** Eriochrome cyanine (**e**) and neurofilament staining (**f**). Scale bar = 200 μm. **g**, **h** Three-dimensional reconstructions of the lesions. Scale bar = 200 μm. **i**–**l** Myelin sparing (**i**), axon sparing (**j**), lesion volume (**k**) and lesion length (**l**) quantification. **m, n** Representative lesion epicenters showing microglia (P2RY12^+^ cells) and MDMs (P2RY12^-^F4/80^+^ cells). Scale bar = 250 μm. **o**–**q** Microglia depletion reduces overall MDMs (**o**) but enables MDMs to migrate beyond the central GM (**p**) into spared tissue (**q**). **r** Representative epicenter ventromedial white matter phagocytic MDMs (CD68^+^P2RY12^-^ cells). Scale bar = 25 μm. **s** Microglia depletion increases the size of phagocytically active MDMs. **t**–**v** GFAP labeling along the spinal axis (**t, u**) and lesion epicenter (**v**). **w, x** Representative astroglial borders at the lesion epicenter Scale bar = 200 μm. **y** Representative ventromedial white matter showing proliferating astrocytes (arrows). Scale bar = 38 μm**. z** Astrocyte proliferation quantification. R, rostral, C, caudal. **b**–**d** Two-way repeated measures ANOVA with Bonferroni post hoc; **b** Group x Time effect: *p* = 0.0013, post hoc group effect: 21d: *p* = 0.008, 28d: *p* = 0.043, 35d: *p* = 0.0044; **c** Group x time effect *p* < 0.0001, post hoc group effect: 21d, 28d, 35: *p* < 0.0001; **d** Group x time effect *p* < 0.0001, post hoc group effect: 20d, 27d, 34d: *p* < 0.0001. **i**–**l**, **p**, **q**, **u**, **v** Student’s two-sided *t* tests; **i**, *p* = 0.001, **j**, *p* = 0.004, **k**, *p* = 0.0006, **l**, *p* = 0.002, **p**, *p* = 0.0045, **q**, *p* = 0.0009, **u**, *p* = 0.0095 **v**, *p* = 0.0007; **o**, **s**, **t**, **z** Two-Way ANOVA with Bonferroni post hoc, mean and SEM, *n* = 7 mice/group; **o** SS = 399.6, df = 13, F = 6.963, *p* = 0.0088; s: 10–20 μm, *p* < 0.0001; 20–30 μm, *p* = 0.025; 100–200 μm, *p* = 0.046; >200 μm, *p* = 0.0013; **t** SS = 459.4, df = 13, F = 38.21, *p* < 0.0001; **z** 0.6 mm rostral, *p* < 0.0001; 0.6 mm caudal, *p* = 0.0007; *p < 0.05; **p < 0.01, ***p < 0.001; ****p < 0.0001. Source data are provided as a Source Data file.

To assess the effects of microglia on functional recovery after SCI, microglia were depleted by feeding mice chow laced with a CSF1R antagonist (PLX5622) beginning two weeks before receiving a 75 kdyne T9 contusion SCI, with feeding continuing until 35 days post-injury (dpi) (Fig. 1a). Quality control studies show that two weeks of continuous feeding is sufficient to deplete 99% of spinal cord microglia (Sup. Fig. 1a–h), and >95% depletion was still achieved by 35 dpi (Sup. Fig. 2)^15^. PLX5622 does not cause locomotor deficits in uninjured mice (Sup. Fig. 1i–o), nor does the drug deplete circulating myeloid-lineage cells or their ability to respond to inflammatory challenges (e.g., LPS) (Sup. Fig. 1 p–z’). These observations confirm published data^16–18^.

All mice, even those without microglia, display normal overground locomotion before SCI, but at 1 dpi, develop near-complete paralysis (Fig. 1b, c). In SCI mice-fed vehicle, locomotor recovery improved gradually but plateaued after 3 weeks (Fig. 1b–d). While mice fed PLX5622 diet recovered similarly to mice fed vehicle diet for 7 dpi, after that they performed worse than controls, with only 2/7 mice (29%) achieving consistent plantar stepping by 35 dpi compared to 100% of controls (Fig. 1b). These exacerbated locomotor deficits were accompanied by severe deficits in trunk stability and tail position from 14–35 dpi (Fig. 1c). Non-subjective behavioral analysis using the horizontal ladder test confirmed the essential role played by microglia in regulating spontaneous recovery of function after SCI. Microglia depletion did not affect baseline performance on this task, but, without microglia, the number of mistakes (i.e., missed ladder rungs) increased at 20, 27, and 34 dpi (*p* < 0.0001 vs. vehicle) (Fig. 1d). Impaired functional recovery was associated with exacerbated myelin and axon pathology (Fig. 1e–l).

Since microglia help maintain tissue homeostasis and coordinate local immune responses, we predicted that depleting microglia would affect the recruitment of monocyte-derived macrophages (MDMs), the predominant infiltrating leukocyte subpopulation after SCI. Consistent with published data^8,19^, reactive macrophages (mostly F4/80^+^P2ry12^−^ MDMs) were restricted to the lesion core in SCI mice fed vehicle (Fig. 1m). Conversely, in SCI mice without microglia, large, ectopic MDM clusters migrated into spared white matter (Fig. 1n–q). MDMs in these clusters were larger and reminiscent of the ‘foamy’ macrophages that have been linked to neurotoxicity and myelin pathology^20,21^ (Fig. 1r, s). Without microglia, the contiguous border of hypertrophied glia (“glial scar”) that normally separates the lesion core from spared white matter, was attenuated (Fig. 1t–x). Since the glial scar is achieved partly through proliferation of astrocytes^22^ and NG2 glia^23^, we quantified the number of proliferating (BrdU^+^GFAP^+^) astrocytes and NG2^+^BrdU^+^ cells in the injured spinal cord. Without microglia, astrocyte (Fig. 1y, z) and NG2 cell proliferation decreased (Sup. Fig. 3). The roles that microglia play in promoting optimal recovery of function and restoring tissue homeostasis through regulation of glial scarring, intraspinal inflammation and limiting axonal dieback, also were evident in more severe models of SCI, including complete forceps crush injuries at the T9 or L1 spinal levels (Sup. Fig. 4). Having replicated the microglia depletion phenotype in these models, we next performed gene sequencing in mice with a 75 kdyne T9 contusion SCI.

### Microglia coordinate the earliest phases of tissue repair after SCI by regulating the injured spinal cord transcriptome

To gain insight into how microglia influence tissue homeostasis after SCI, sham SCI spinal cords from mice-fed vehicle or PLX5622 were analyzed using bulk RNA sequencing. Since microglia are exquisitely sensitive to changes in tissue homeostasis^24^, we predicted that microglia-dependent effects on the injured spinal cord transcriptome would be evident by 7 dpi.

**Fig. 2:**
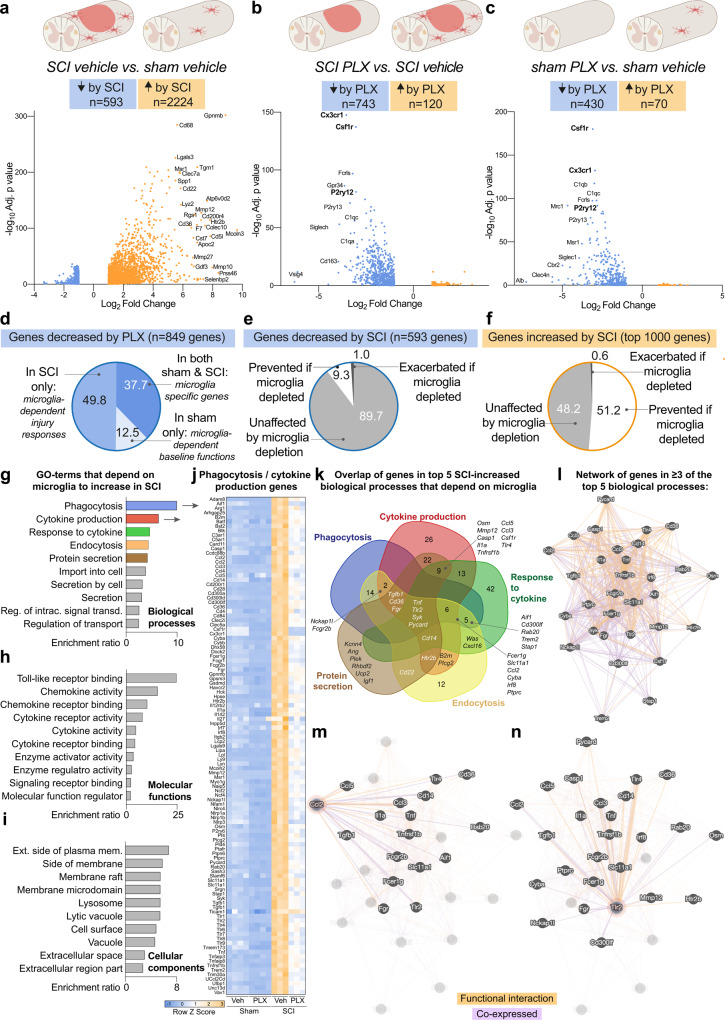
RNA sequencing shows microglia control immune responses to SCI. **a**–**c** Volcano plots showing SCI-dependent genes (**a**), microglia-dependent genes in SCI (**b**), and microglia-dependent genes at baseline (**c**). Selected genes that changed the most are labeled. The main effect of SCI was increased gene expression, and the main effect of microglia depletion was decreased gene expression. **a**–**c** Data generated from analysis performed In DeSeq2 (Wold test) and Gene Network Analyst. **d** Pie chart showing more genes were decreased by microglia depletion in the SCI condition than the sham condition. **e**, **f** Analysis of SCI-dependent genes shows microglia depletion only had a modest effect on SCI-decreased genes (**e**), but prevented an increase in most of the top (51.2%) SCI-increased genes (**f**). **g**–**i** The 512 genes increased by SCI that failed to increase when microglia were depleted were entered into pathway analysis for biological processes (**g**), molecular functions (**h**) and cellular components (**i**). **j** Heatmap showing specific genes involved in the biological processes ‘Phagocytosis’ and/or ‘Cytokine production’ (scale = Z scores of FPKM values). **k** Venn diagram showing overlap between genes in the top 5 biological process. **l** Network analysis showing co-expression (purple) and predicted functional interactions (orange) between genes that overlapped in ≥3 of the top 5 biological processes (*n* = 31). **m**, **n** Interactions between the Ccl2 edge node (**m**) and Tlr2 center node (**n**) with other genes in the network. *N* = 3-4 mice per group. Source data are provided as a Source Data file. See also Supplemental Tables 1–3.

At 7 dpi, the main effect of SCI was to increase gene expression (Fig. 2a). Conversely, the main effect of microglia depletion was to decrease gene expression in injured spinal cord (Fig. 2b). The reduction in gene expression after microglia depletion cannot be explained by a loss of microglia intrinsic genes. Indeed, microglia-specific genes account for only 37.7% (320/849) of reduced genes in both sham and SCI groups (Fig. 2b–d). In contrast, 422 unique genes (~50% of all genes reduced by PLX) were reduced only in the SCI group; these are *microglia-dependent* genes (Fig. 2d).

Next, we determined the nature of the post-SCI transcriptional changes that are controlled by microglia. Although the main effect of SCI was to increase gene expression, 593 genes were downregulated after SCI, and most (538/593 or 89.7%) were unaffected by microglia depletion (Fig. 2e). In contrast, without microglia, 51.2% of genes that were normally increased after SCI (512 of the top 1000) were no longer increased (Fig. 2f). These microglia-dependent genes encode proteins required for: lipid transport/processing, cell cycle, complement, cytokines, pattern recognition receptors, interferons, interleukins, and other factors related to injury responses, proliferation, and tissue remodeling (Supplemental Table 1).

Next, using the GEne SeT AnaLysis Toolkit (WebGestalt)^25^, we determined that microglia-dependent genes control the following major biological processes after SCI: phagocytosis, cytokine production, response to cytokines, endocytosis and protein secretion (Fig. 2g, j). The main microglia-dependent molecular functions increased by SCI were toll-like receptor binding, chemokine activity, chemokine receptor binding, cytokine receptor activity, and cytokine activity (Fig. 2h). In line with this, the main cellular components affected by SCI and microglia depletion were membrane domains and structures involved in endocytosis (e.g., exterior side of plasma membrane, lysosome, vacuole) (Fig. 2i). Additional analysis using the Reactome pathway database^26^ confirmed that after SCI, microglia are important for activating signal transduction, immune responses, metabolism, metabolism of proteins, hemostasis and cell cycle (Supplemental Table 2). Gross and histopathological observation of spinal cords with and without microglia at 7, 14 and 21 dpi confirmed the predictions made from bulk RNAseq data, i.e., that microglial coordinate the cellular and molecular processes needed to achieve optimal hemostasis, preservation of myelin and axons, phagocytosis of myelin debris, controlled spatiotemporal recruitment of MDMs, and glial border formation (Sup. Fig. 5).

To understand if these microglia-dependent effects are encoded by a consistent genetic “signature”, a Venn diagram was created to visualize genes in the top 5 biological processes that were both increased after SCI and were also microglia-dependent (Fig. 2k). Although most genes (*n* = 140/171) were involved in only 1 or 2 processes, 31 genes were involved in ≥3 biological processes: 17 genes were involved in 3 processes; 10 genes were involved in 4 processes; and 4 genes (*Tnf*, *Tlr2*, *Syk*, and *Pycard*) were involved in all of the top 5 biological processes (Fig. 2k, Supplemental Table 3). Gene network analysis, with linkages of co-expression and predicted functional interactions, was performed to understand how these 31 genes were interrelated (Fig. 2l). Every gene connected to at least 8 others by co-expression and/or functional interaction, indicating that this gene network is a “core” transcriptional signature of microglia-dependent functions after SCI. Indeed, using GeneMANIA (https://genemania.org) to explore gene interactions, we found that a key SCI-dependent and microglia-dependent node positioned at the edge of the network was *Ccl2* (Fig. 2m), and a key node positioned at the center of the network was *Tlr2* (Fig. 2n).

### Restoring microglia-dependent signaling networks in microglia-depleted spinal cord promotes tissue healing and neurological recovery

If the “core” transcriptional signature of microglia-dependent functions identified above is required to achieve homeostatic neuroinflammation, gliosis, and vascular repair after SCI, then it should be possible to restore tissue homeostasis to the injured spinal cord and improve recovery of function in microglia-depleted mice by introducing into the injury site, key microglia-dependent signaling molecules. Notably, *Tlr2* and *Ccl2* were notably deficient in the injured spinal cord of microglia-depleted mice (Fig. 2k–n). Since CCL2 controls intraspinal MDM recruitment^27^, and TLR2 agonists confer neuroprotection after SCI^28–30^, we posited that injecting a combination of recombinant CCL2 (rCCl2) and PAM3CSK4, a TLR2 agonist (TLR2 ag) into the injury site would reverse the microglia depletion phenotype.

**Fig. 3:**
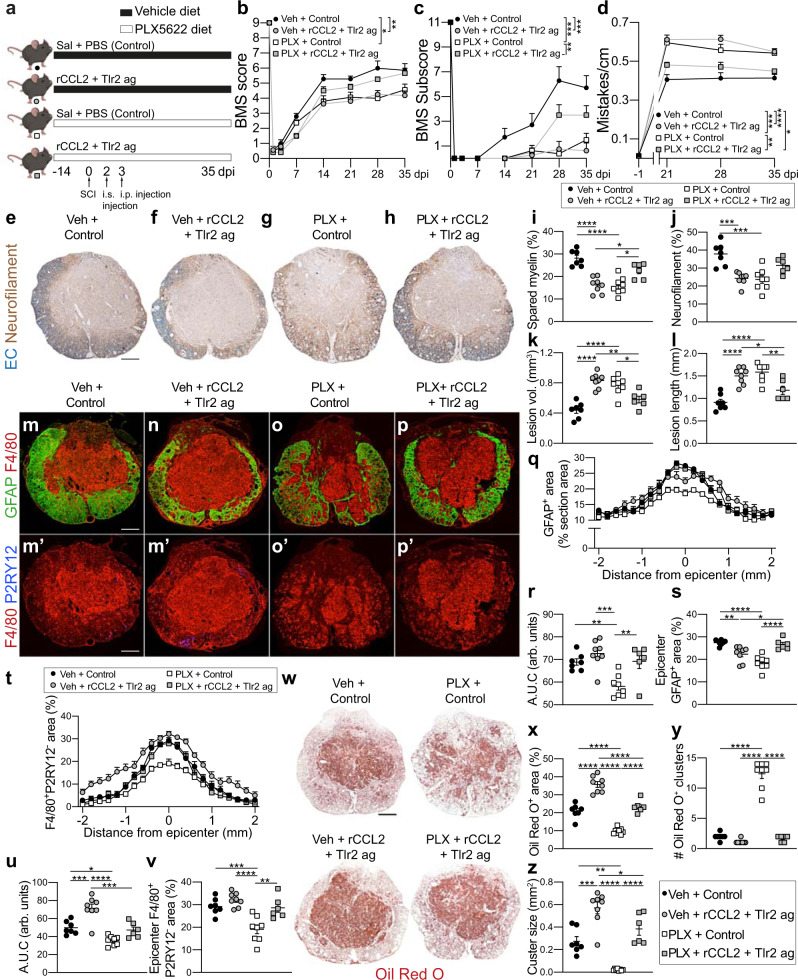
Treatment with rCCL2 and Tlr2 ag rescues the microglia depletion phenotype. **a** Experimental groups and timeline. Mice were continuously fed vehicle or PLX5622 (PLX) and microinjected intraspinally (i.s.) with rCCL2 (or saline) and a Tlr2 agonist (PAM2CSK4, Tlr2 ag) (or saline) at 2 dpi and i.p. with Tlr2 ag (or PBS) at 3 dpi. Behavior was monitored until the end point for tissue collection at 35 dpi. **b**–**d** Microglia depletion worsened SCI recovery based on BMS scores (**b**), subscores (**c**) and horizontal ladder (**d**); rCCl2 and Tlr2 ag improved motor recovery in mice with microglia depletion but worsened outcomes in mice with microglia. **e**–**l** Reprograming the MDM phenotype improved tissue sparing in microglia-depleted mice but worsened pathology in control mice. Scale bar, **e**–**h** = 200 μm. **m**–**s** Glial scarring (**m**–**p, q**–**s**) and MDM pathology (**m**–**p, r**–**v**) in microglia-depleted mice was rescued by rCCL2 and TLR2 ag. Scale bar, **m**–**p** = 220 μm. **w**–**z** Lipid phagocytosis as measured by Oil Red O showed rCCL2 and TLR2 ag improved lipid phagocytosis in microglia depleted mice. Scale bar (**w**) = 200 μm. **B**–**D**: Two-Way repeated measures ANOVA with Bonferroni post hoc; **i**–**l, r, s, u, v, x**–**z**: One-way ANOVA with Bonferroni post-hoc tests; **q, t** Left is rostral; Two-Way ANOVA with Bonferroni post-hoc tests; **q** SS = 1135, df = 28, F = 40.19, *p* < 0.0001; **T** SS = 6674, df=28, F = 143.7, *p* < 0.0001; **b-d, i-l, q, r-v, x-z**: *n* = 6–8 mice per group (*n* = 7 Veh + control, *n* = 8 Veh + rCCL2 + Tlr2 ag; *n* = 8 PLX + Control, *n* = 6 PLX + rCCL2 + Tlr2 ag); mean ± SEM; *p < 0.05; **p < 0.01; ***p < 0.001; ****p < 0.0001. A.U.C, Area under the curve. Source data are provided as a Source Data file.

Mice were fed vehicle or PLX5622 starting two weeks before SCI, and then at 2 dpi, coinciding with the normal peak of endogenous CCL2 production^31^ and *Tlr2* expression^28,32^, vehicle or rCCL2 and TLR2 ag were injected into the injury site in an effort to recruit and then reprogram infiltrating MDM phenotypes. In these same mice, at 3 dpi, preceding peak MDM recruitment after experimental SCI^8^, TLR2 ag was injected (i.p.). Pilot studies revealed this to be the optimal paradigm for enhancing MDM recruitment and lipid phagocytosis in animals without microglia (Sup. Fig. 6). When this paradigm was applied in an independent replicate experiment with survival extended to 35 dpi (Fig. 3a), rCCL2 + TLR2 ag injections prevented the exacerbated pathology and functional impairment caused by microglia depletion (Fig. 3b–v). However, in SCI mice with microglia, the same treatment impaired neurological recovery (Fig. 3b–d) and exacerbated lesion pathology (Fig. 3e–z). When sections from each group were stained for GFAP and Oil Red O, it was evident that re-introduction of microglia-dependent signals, i.e., rCCL2 + TLR2 ag, restored lipid binding, phagocytosis, glial scarring, MDM corralling, i.e., cellular functions that were otherwise impaired in microglia-depleted injured spinal cords (Fig. 3w–z). Collectively, these data indicate that post-injury induction of CCL2 and TLR2 signaling, a “core” transcriptional signature of microglia-dependent functions revealed by bulk RNAseq, is needed to effectively coordinate the restorative/reparative effects of neuroinflammation and reactive astrogliosis after SCI. However, interfering with or amplifying this microglia-dependent signaling network worsens injury outcomes.

### Single cell RNA sequencing (scRNA-seq) reveals time-dependent and cell-specific changes in the injured spinal cord transcriptome

**Fig. 4:**
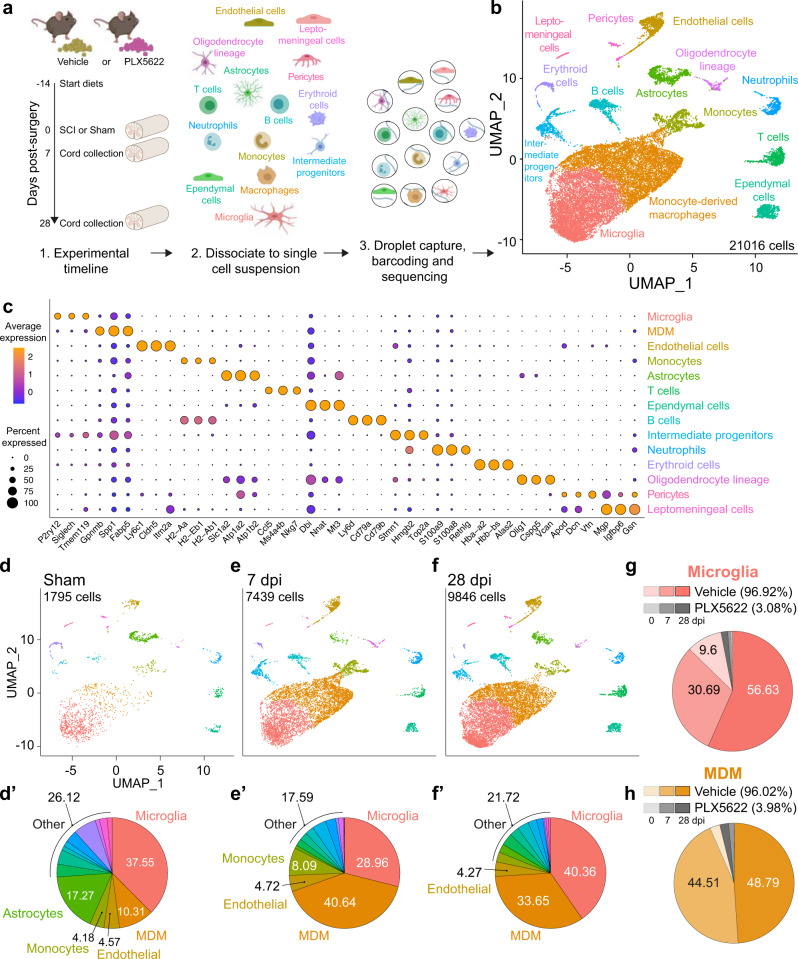
Single-cell RNA sequencing of the mouse spinal cord reveals changes in cell composition after SCI and microglia depletion. **a** Mice received either vehicle or PLX5622, starting 2 weeks before surgery, until 7 or 28 days post-surgery. At the end point, single-cell RNA sequencing was performed on fresh (un-perfused) mouse spinal cords by dissecting a 1 cm piece of fresh tissue (T4–T13), including the meninges, centered on the laminectomy site. This method optimizes cell viability, allows comparison of leukocyte profiles present in both sham and SCI conditions, and enriches for resident glia and peripheral immune cells. **b** UMAP plot showing fourteen major cell types pooled from all groups. Each dot represents a single cell. **c**: Dot Plot showing the top 3 DEGs in each cluster based on adjusted p values. The dot color indicates the average scaled RNA expression of that gene in the cell type, and the dot size represents the percentage of cells in the cluster that express that gene. **d**–**f** UMAP plots of vehicle-fed mice split by time point. **d’**–**f’** The percentage of cell types in **d**–**f**. **g**, **h** Pie chart showing that most of the microglia (**g**) and MDMs (**h**) were derived from samples in the vehicle group. Data for each condition were pooled from *n* = 3–4 mice. Source data are provided as a Source Data file. See also Sup. Fig. 7.

To better understand how microglia coordinate interactions between various cell types to achieve optimal post-injury tissue homeostasis and recovery of function^33,34^, single-cell RNA sequencing (scRNA-seq) was used. RNA was prepared and barcoded from 21,016 single cells (16,003 genes) isolated from spinal cords pooled from sham, 7 dpi, or 28 dpi mice fed vehicle or PLX5622 (Fig. 4a). Uniform Manifold Approximation and Projection for Dimension Reduction (UMAP)^35^ clustering and annotation revealed 14 cell types that have transcriptional profiles consistent with: microglia (33.4%), MDMs (32.3%), endothelial cells (5.1%), monocytes (5.1%), astrocytes (4.6%), T cells (3.7%), ependymal cells (3.4%), B cells (3.1%), intermediate progenitors (2.9%), neutrophils (2.5%), erythroid cells (1.3%), oligodendrocyte lineage (1.2%), pericytes (0.8%), and leptomeningeal cells (0.6%) (Fig. 4b). Cell types were confirmed by identifying the most significant differentially expressed genes (DEGs) for each cluster (Fig. 4c, Sup. Fig. 7) then cross-referencing DEG profiles with independent libraries^36,37^. Microglia and MDM clusters were over-represented in our integrated library, likely a result of capture-bias and/or high tolerance of these cells to the dissociation process^38^. Monocle 3 was used to further assess microglia and MDM cluster robustness across pseudotime. When pseudotime information was extracted and reduced to one dimension, we found extensive separation between microglia and MDM peaks, with ~80% of cells uniquely identified as microglia and ~73% of cells uniquely identified as MDMs. Thus, only ~20% of microglia and ~27% of MDMs could be considered ambiguously clustered “CNS macrophages” located in the interface between microglia and MDMs.

In vehicle and PLX5622 groups, SCI clearly affected the composition of spinal cord cells, including those in blood vessels and meninges (Fig. 4d–f). At baseline, >50% of cells were resident glia (37.55% microglia; 17.27% astrocytes), with fewer numbers of endothelial cells and vessel-associated leukocytes and MDMs (Fig. 4d, d**’**). However, after SCI, as MDMs infiltrate the injured tissue (MDMs, sham = 10.31%, 7 dpi = 40.64%, 28 dpi = 33.65%), the proportion of resident glia decreased (Fig. 4d’–f’). Overall, due to microglia depletion (Fig. 4g) and a decrease in MDM recruitment (Fig. 4h**;** Sup. Fig. 5g–j), fewer cells were isolated from spinal cords of mice fed PLX5622 compared to vehicle (PLX5622 = 1,936 vs. vehicle = 19,080).

### Functional heterogeneity of microglia in injured spinal cord revealed by scRNA-seq

**Fig. 5:**
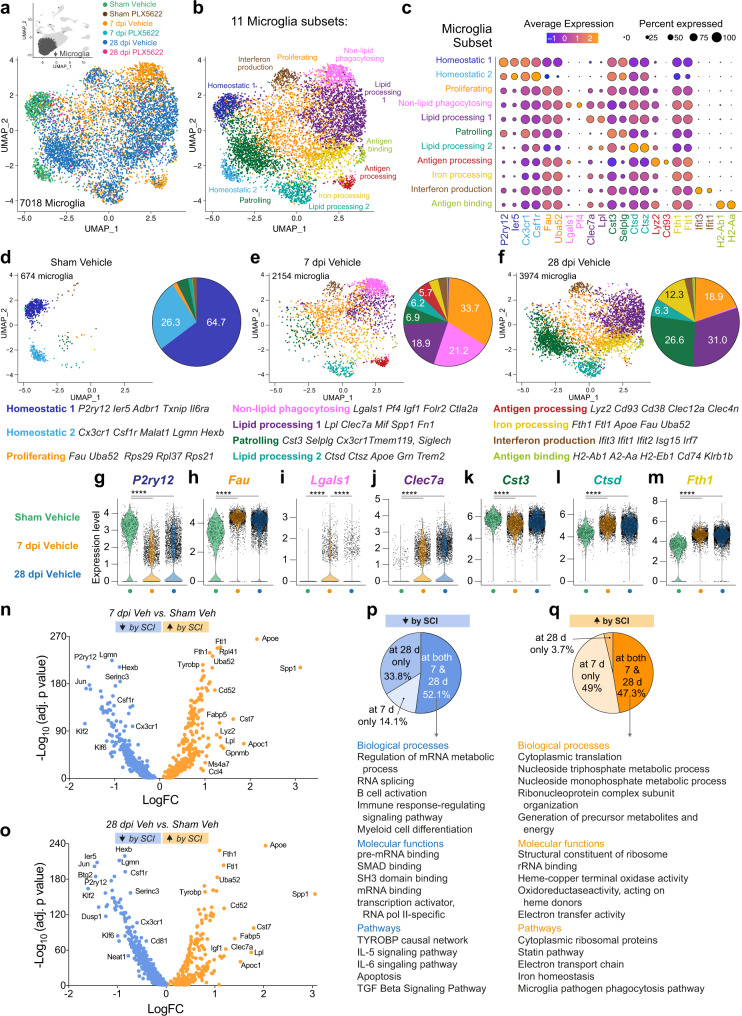
Microglia adopt diverse, the time-dependent transcriptional phenotype in response to SCI. **a** Microglia were computationally isolated for analysis between experimental conditions. **b**, **c** Microglia is subdivided into 11 subsets based on dominant gene expression. **d**–**f** UMAP plots showing the microglia subsets split by time post-SCI. Numbers in pie charts show major subsets as a percent of total microglia in each group. **g**–**m** Violin plots showing gene-level changes within individual microglia. Wilcox rank-sum tests, ****p < 0.0001. **n**, **o** Volcano plots showing increased genes (orange) and decreased genes (blue) in microglia following SCI at 7 dpi (**n**) or 28 dpi (**o**). **n**–**o** Wilcox rank-sum tests. Selected genes are labeled. **p** Most of the microglia genes decreased at 7 dpi remained decreased at 28 dpi; WebGestalt gene ontology and pathway analysis showed these genes control immune regulation and myeloid cell differentiation. **q** Less than half of the microglia genes increased at 7 dpi were also increased at 28 dpi; WebGestalt gene ontology and pathway analysis showed consistently increased genes control cell metabolism, translation, and response to heme, oxidants, and phagocytosis. Genes exclusively increased in microglia at 7 dpi control aspects of chemotaxis and cell metabolism. Data for each condition were pooled from *n* = 3-4 mice. Source data are provided as a Source Data file. See also Sup. Fig. 8.

Data in Fig. 1 and Sup. Fig. 5 indicates that after SCI, microglia become activated and coordinate intercellular communication to promote tissue repair. To determine how SCI affects the functional potential of microglia, we computationally selected the microglia cluster from SCI mice to reveal microglia-specific gene expression changes before and after SCI (Fig. 5a). Eleven major microglia subsets were identified (Fig. 5b, c, Sup. Fig. 8): (1) ‘Homeostatic 1’ and (2) ‘Homeostatic 2’ expressed high levels of *P2ry12* and *Csf1r;* (3) ‘Proliferating’ expressed ribosomal genes (e.g., Fau, Rps); (4) ‘Non-lipid phagocytosing’, high *Lgals1*;^39^ (5) ‘Lipid processing 1’, high *Lpl*; (6) ‘Patrolling’, high homeostatic-like markers (e.g., *Selplg*); (7) ‘Lipid processing 2’, high Ctsd; (8) ‘Antigen processing’, high Lyz2; (9) ‘Iron processing’, high Fth1; (10) ‘Interferon production’, high Ifit3; and (11) ‘Antigen binding’, high H2-Aa1.

To determine the predominant microglia phenotype before and after SCI, we quantified the proportion of each microglia subset in vehicle samples at baseline and also at 7 and 28 dpi (Fig. 5d–f). At baseline, Homeostatic 1 (64.7%) or Homeostatic 2 (26.3%) microglia predominate (Fig. 5d). However, by 7 dpi, homeostatic microglia are replaced by Proliferating (33.7%), Non-lipid phagocytosing (21.2%) and Lipid processing 1 microglia (18.9%) (Fig. 5e). By 28 dpi, Non-lipid phagocytosing microglia were replaced by Lipid processing 1 microglia (31%), and also Patrolling (26.6%), Proliferating (18.9) and Iron processing (12.3%) microglia (Fig. 5f). Lipid processing 2 microglia also increased from baseline (from 1.5% to >6%) at both 7 and 28 dpi. When microglial gene expression was analyzed on a per-cell basis, representative microglia homeostatic genes decreased after SCI (Fig. 5g), whereas proliferating (Fig. 5h), lipid processing (Fig. 5j, l) and iron processing **(**Fig. 5m) genes increased; non-lipid phagocytosing genes increased transiently at 7 dpi (Fig. 5i).

Over half (52.1%) of microglia genes that were decreased at 7 dpi were also decreased at 28 dpi (Fig. 5n–p). Consistently decreased genes (e.g., *P2ry12, Hexb, Lgmn, Csf1r, Cx3cr1*) are those that regulate mRNA metabolism, immune signaling, differentiation, and apoptosis (Fig. 5p**)**. When we explored microglia genes that were increased by SCI, 47.3% were increased at both 7 and 28 dpi (Fig. 5n, o, q). Consistently increased genes (e.g., *Apoe, Spp1, Fth1*) were those that enhance translation, transcription, cell metabolism, proliferation, response to heme and oxidants, lipid processing (statin) pathways, and pathogen phagocytosis (Fig. 5q). Almost half (49.0%) of the genes increased in microglia were only increased at 7 dpi. These were also related to enhancing metabolic activity and immune function. Few (3.7%) genes were increased only at 28 dpi: *C1qa, Ly6e, Ccl12, Rgs1, B2m, Ccnd1, Cacybp, Itgax, Sf3b6, Lasp1, Jtb, Kcmf1, Ccl6, Cd74, Btf3l4, Ube2d3, and Kdelr2* (Fig. 5q). Together, these data validate the bulk RNA seq data (Fig. 2) and also show that the main response of microglia to SCI is increased transcription of genes that control proliferation, protein translation, metabolic activity, iron and lipid processing, and immune responses.

### Microglia control the recruitment and function of MDMs after SCI

**Fig. 6:**
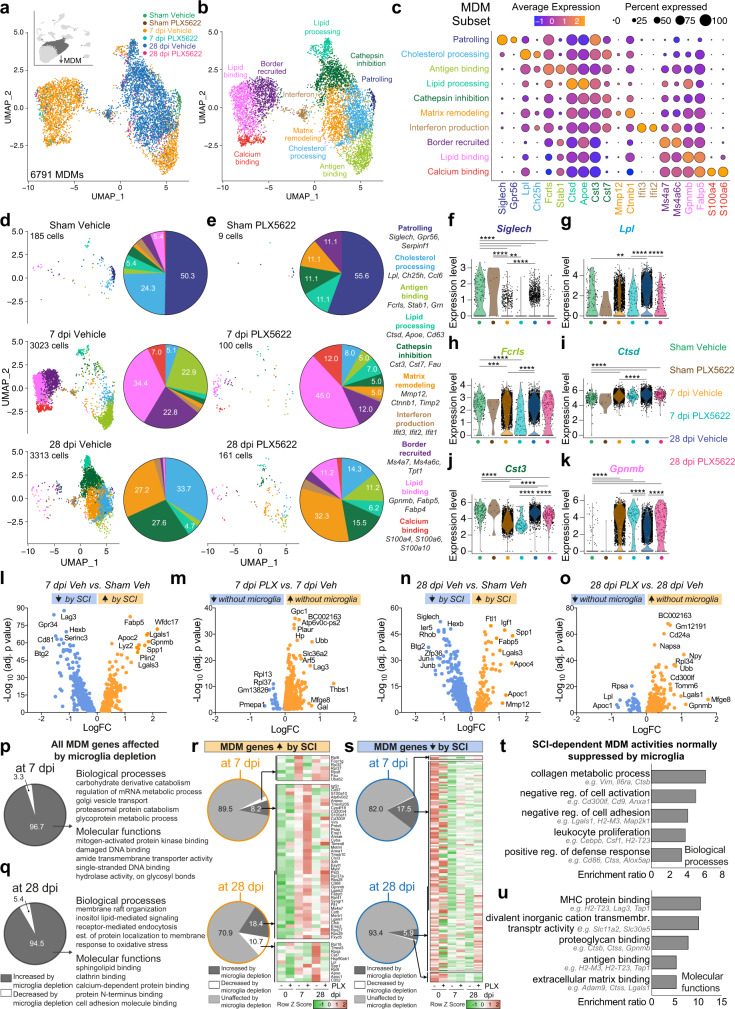
Monocyte-derived macrophages (MDMs) adopt diverse, time-dependent transcriptional phenotype in response to SCI. **a** MDMs were computationally isolated for analysis between experimental conditions. **b**, **c** MDMs subdivided into 10 subsets based on dominant gene expression. **d**, **e** UMAP plots showing MDM subsets split by diet group and time post-SCI. Numbers in pie charts show major subsets as a percent of total MDMs in each group. **f**–**k** Violin plots showing gene-level changes within individual MDMs. Wilcox rank-sum tests, **p < 0.01, ***p < 0.0001, ****p < 0.0001. **l**–**o** Volcano plots showing increased genes (orange) and decreased genes (blue) in following SCI and microglia depletion. Selected genes are labeled. Wilcox rank-sum tests. **p**, **q** Pie charts of data in **m** and **o** showing that microglia depleti**o**n mostly caused an increase in MDM genes. Gene ontology shows these increased genes carbohydrate metabolism, calcium signaling and cell adhesion. **r**, **s** The effect of microglia depletion on MDM genes that were increased (**r**) or decreased (**s**) by SCI (see Sup. Fig. 10 for gene names in **s**). **t**, **u** Pathway analysis of all MDM genes affected by SCI and 7 or 28 dpi and also increased by microglia depletion, showing that microglia normally suppress several biological processes (**t**) and molecular functions (**u**) in intraspinal MDMs after inj**u**ry. Data for each condition were pooled from *n* = 3–4 mice. Source data are provided as a Source Data file. See also Sup. Figs. 9 and 10.

Next, to understand how MDMs respond to SCI and also how microglia regulate MDM functions, we computationally selected the MDM cluster (Fig. 6a). Injury and time-dependent differences in MDM gene expression were noted (Fig. 6a), with 10 major MDM subsets identified (Fig. 6b, c, Sup. Fig. 9). These subsets were: (1) ‘Patrolling’ - high *Siglech*; (2) ‘Cholesterol processing’ - high Lpl; (3) ‘Antigen binding’ - high Fcrls; (4) ‘Lipid processing’ - high Ctsd; (5) ‘Cathepsin inhibition’ - high Cst3; (6) ‘Matrix remodeling’ - high Mmp12; (7) ‘Interferon production’ - high Ifi*t3*; (8) MDMs transiently recruited that are high in ‘CNS border associated genes’ (e.g., Ms4a7)^40^, termed ‘Border recruited’ MDMs; (9) ‘Lipid binding’ - high Gpnmb; and (10) ‘Calcium binding’ - high S100a4. Although few MDMs could be collected from uninjured spinal cords, Patrolling (50.3%) and Cholesterol processing (24.3%) MDM subsets predominated at baseline (sham vehicle, Fig. 6d). By 7dpi, these subsets were replaced by Lipid binding (34.4%), Antigen binding (22.9%), Border recruited (22.8%) and Calcium-binding MDMs (7%). By 28 dpi, Cholesterol processing MDMs again comprise a major subset (33.7%), and different MDM subsets emerge, including Cathepsin inhibition (27.6%) and Matrix remodeling MDMs (27.2%).

Microglia depletion limited the number of MDMs present, and also skewed the phenotype of MDMs in the injured spinal cord at 7 and 28 dpi (Fig. 6e). Indeed, at 7 dpi without microglia, Lipid binding (45% vs. 34.4%), Calcium-binding (12% vs. 7%) and Lipid processing (7% vs. 2%) MDMs predominated, with fewer Antigen binding (5% vs. 22.9%) and Border recruited MDMs (12% vs. 22.8%) present (Fig. 6e). At 28 dpi without microglia, Matrix remodeling (32.3% vs. 27.2%) and Lipid processing (6.2 vs. 4.7%) MDMs predominated, and fewer Cathepsin inhibition (15.5% vs. 27.6%) and Cholesterol processing MDMs (14.3% vs. 33.7%) were present (Fig. 6e). The SCI and microglia-dependent effects on MDM clusters were also assessed by analyzing gene expression on a per-cell basis. Figure 6f–k shows selected gene changes from 6 predominant MDM subsets, with Patrolling genes decreased after SCI (Fig. 6f) but Cholesterol processing (Fig. 6g), Lipid processing (Fig. 6i) and Lipid binding (Fig. 6k) genes increased at 7 and/or 28 dpi. Microglia depletion decreased Cholesterol processing (Fig. 6g) and Cathepsin inhibition genes (Fig. 6j) but increased Lipid binding genes (Fig. 6k) at 28 dpi.

At 7 and 28 dpi, the main effect of SCI on MDMs was to increase their expression of genes related to inflammation and lipid processing (e.g., *Cd68*, *Spp1*, *Lgals3*, *Vim*, *Ctsd*, *Cstb*, *Gpnmb*, *Cd63*, *Lyz2*, *Apoe*, and *Igf1*), although genes controlling antigen processing and chemotaxis (e.g., *Cd38*, *Cd28*, *Lrp1*, *Cxcr4*, *Cd36*, *Il7r*, *Lrp1*, *Clec12a)* and matrix remodeling (e.g., *Mmp12*, *Fn1*, *Cspg,* and *Timp2*) were also increased in MDMs at 7 or 28 dpi, respectively (Fig. 6l, n).

The main effect of microglia depletion was to increase overall gene expression in MDMs (Fig. 6m, o). At 7 dpi, these genes were mainly related to carbohydrate metabolism and vesicle transport (Fig. 6p). At 28 dpi, MDM genes increased by microglia depletion were mostly related to cell adhesion and cell membrane reorganization (Fig. 6q).

Next, we identified those MDM genes that were increased (Fig. 6r) or decreased (Fig. 6s) by SCI and were also affected by microglia depletion. As predicted from Fig. 6p**, q**, the main effect of microglia depletion on SCI-dependent MDM genes was increased gene expression (Fig. 6r, s, Sup. Fig. 10 for gene names). GO term analyses of all MDM genes affected by SCI and also increased without microglia showed that microglia normally inhibit several functionally relevant pathways in MDMs including collagen metabolism, antigen and extracellular matrix protein binding, and negative regulation of cell activation and cell adhesion (Fig. 6t, u). Thus, microglia control both the intraspinal recruitment of MDMs and their functional potential upon entry into the lesion site. Without microglia, MDM recruitment is impaired, MDMs fail to adhere to and migrate within the primary lesion zone, and those that do are poised for unchecked matrix degradation.

### scRNA-seq reveals SCI- and microglia-dependent effects on astrocyte transcriptional profiles

**Fig. 7:**
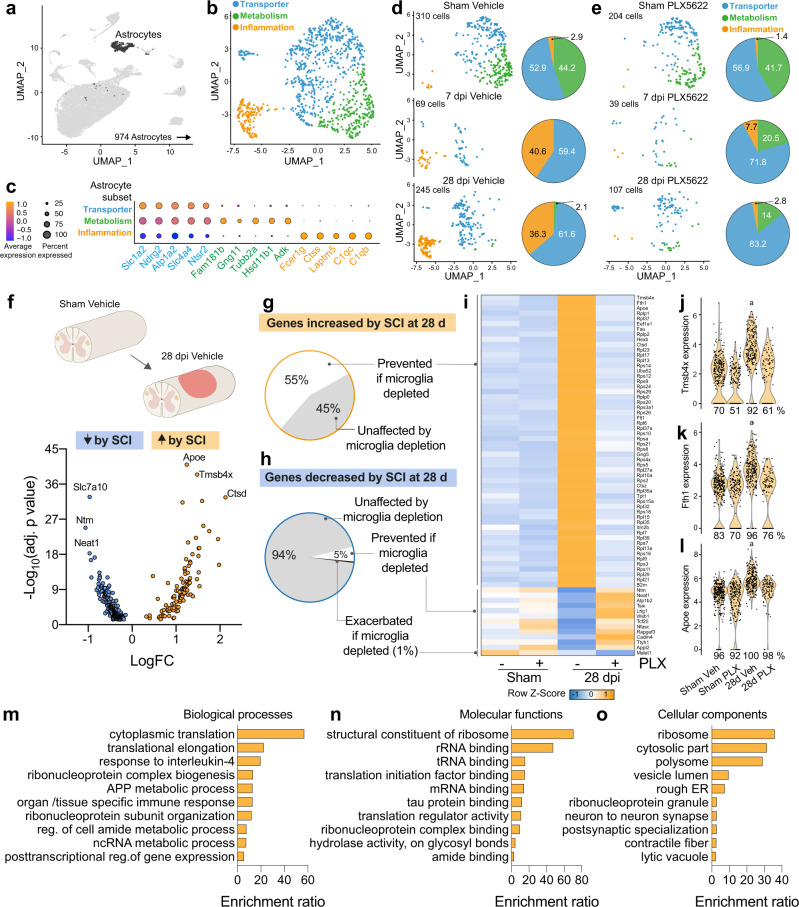
Microglia depletion impairs SCI-induced transcriptional changes in astrocytes. **a** Astrocytes were computationally isolated for analysis between experimental conditions. **b** Astrocytes were divided into three clusters based on dominant gene expression, and were characterized as: Transporter (green), Metabolism (blue), and Inflammation (orange). **c** Dot Plot showing the top DEGs in each cluster. **d**, **e** UMAP plots showing astrocytes in the vehicle (**d**) and PLX5622 (**e**) conditions. The Inflammation astrocyte subset increases proportion over time post-SCI only when microglia are present. Numbers in pie charts show each subset as a percent of total astrocytes in each group. **f** To determine how microglia depletion affected long-term SCI-induced changes in astrocytes, we compared 28 dpi astrocytes to sham vehicle astrocytes. Select genes are labeled. **g** Of all the genes increased in astrocytes at 28 dpi, 55% failed to increase if microglia are absent. **h** Of all the genes decreased in astrocytes at 28 dpi, 5% failed to decrease if microglia are absent. **i**: Heatmap showing SCI-dependent and microglia-dependent astrocyte genes. **j**–**l** Violin plots showing the first 3 genes in **h**. The percentages under each violin indicate the percent of cells in the group that express the gene^a^. *p*_adj_ < 0.0001 compared to other groups, Wilcox rank-sum test, *n* = 107–310 astrocytes per group pooled from 3–4 mice per group. **m**–**o** Astrocyte genes increased by SCI and reversed by microglia depletion were entered into gene ontology (GO) term analysis using WebGestalt. Astrocyte biological processes (**l**), molecu**l**ar functions (**m**), and cellular components (**n**) related to translation, cell proliferation, metabolism, and inflammation were impaired by microglia depletion. Source data are provided as a Source Data file.

Because microglia depletion disrupts the stereotypical formation of a delimiting astroglial border at the injury site (Fig. 1, Sup. Figs. 4 and 5), a phenotype that is associated with the spreading of MDMs into spared tissue, we next evaluated the effects of microglia depletion on the astrocyte transcriptome after SCI. Computational isolation of the astrocyte cluster revealed three major astrocyte subsets based on DEGs broadly related to: 1) solute Transporter, (e.g., *Slc1a2, Atp1a2*), 2) Metabolism, (e.g., *Gng11, Adk*), and 3) innate immune Inflammation, (e.g., Fc*er1g, C1qc*) (Fig. 7a-c). Separation of the UMAP plot by experimental condition revealed that in control spinal cords (sham vehicle), Metabolism (52.9%) and Transporter (44.2%) astrocytes predominate (Fig. 7d). However, at 7 and 28 dpi, Metabolism astrocytes are replaced by Transporter and Inflammation astrocytes (Fig. 7d). At 28 dpi, a time corresponding with the formation of a “mature” astroglial border in vivo^41^ 101 astrocyte genes increased (e.g., *C1qb, Fau, Tmsb4x, Rps genes, ApoE, H2-D1, Fth1,* and *Ctsd*), and 225 genes decreased (e.g., *Ntm, Neat1, Vegfa, Nfix,* and *Kcna2*) relative to sham (Fig. 7f). Notably, regardless of time post-injury, the SCI-dependent increase in Inflammation astrocytes was microglia-dependent; few Inflammation astrocytes were found in spinal cords without microglia (Fig. 7e). Of all genes that were increased in astrocytes at 28 dpi, 55% failed to increase without microglia (Fig. 7g, i). These genes control cell proliferation (e.g., *Rpl-*, *Rps-*, *Fau*), lipid processing (e.g., *Apoe, Ctsz, Ctsd*), and antigen presentation (*B2m*)^42^ (Fig. 7g–l). A few (5%) astrocyte genes that were normally decreased after SCI, (including the cell adhesion genes *Ntm, Nfasc, Cadm4*, and also *Neat1, Atp1b2, Tsix, Lrig1, Wsb1, Tcf25, Rapgef3, Ttyh1*, and *Appl2*), did not decrease if microglia were depleted (Fig. 7h, i). GO term analyses revealed that depleting microglia prevents the biological processes and molecular functions, and cellular components that control in astrocytes, among other things, cytoplasmic translation, response to IL-4, RNA binding, and tau protein binding (Fig. 7m–o). Together these data show that many stereotypical astrocyte-specific functions triggered by SCI, particularly inflammatory gene upregulation, lipid processing, cell adhesion, and proliferation, are controlled by microglia.

Although microglia exert major effects on MDMs and astrocytes, scRNA-seq also revealed microglia-dependent effects on other cell types that help to explain anatomical and functional outcomes after SCI in mice depleted of microglia. For example, the persistence of blood in injured spinal cords without microglia (Sup. Fig. 5) could indicate an increase in hemorrhage due to a failure of Homeostatic endothelial cells to shift toward Differentiating and Neovascularizing endothelial phenotypes or impaired clearance/phagocytosis of blood from the lesion site (Sup. Fig. 11a–e). Microglia control the expression of endothelial cell genes that are essential for vascular repair, including those controlling proliferation, vessel remodeling, solute transport, and responsiveness to inflammatory molecules (Sup. Fig. 11f–l). SCI- and microglia-dependent effects were also observed in: ependymal cells (particularly lipid processing genes; e.g., *Apoe*, *Ctsd*, *Tyrobp*, *Arl6*, *Prnc2*, and *Id4*; Sup. Fig. 12), oligodendrocyte lineage cells (e.g., *Tmsb4x*, *Ntm*; Sup. Fig. 13), neutrophils (e.g. *Cxcr4*, *Pram1*, *Ier3*; Sup Fig. 14), and immature monocytes (differentiation and antigen-presenting genes e.g., Rpl/Rps genes, *Ccnd3*, *H2-T23*; Sup Fig. 15).

### Microglia-dependent signaling networks in the injured spinal cord

**Fig. 8:**
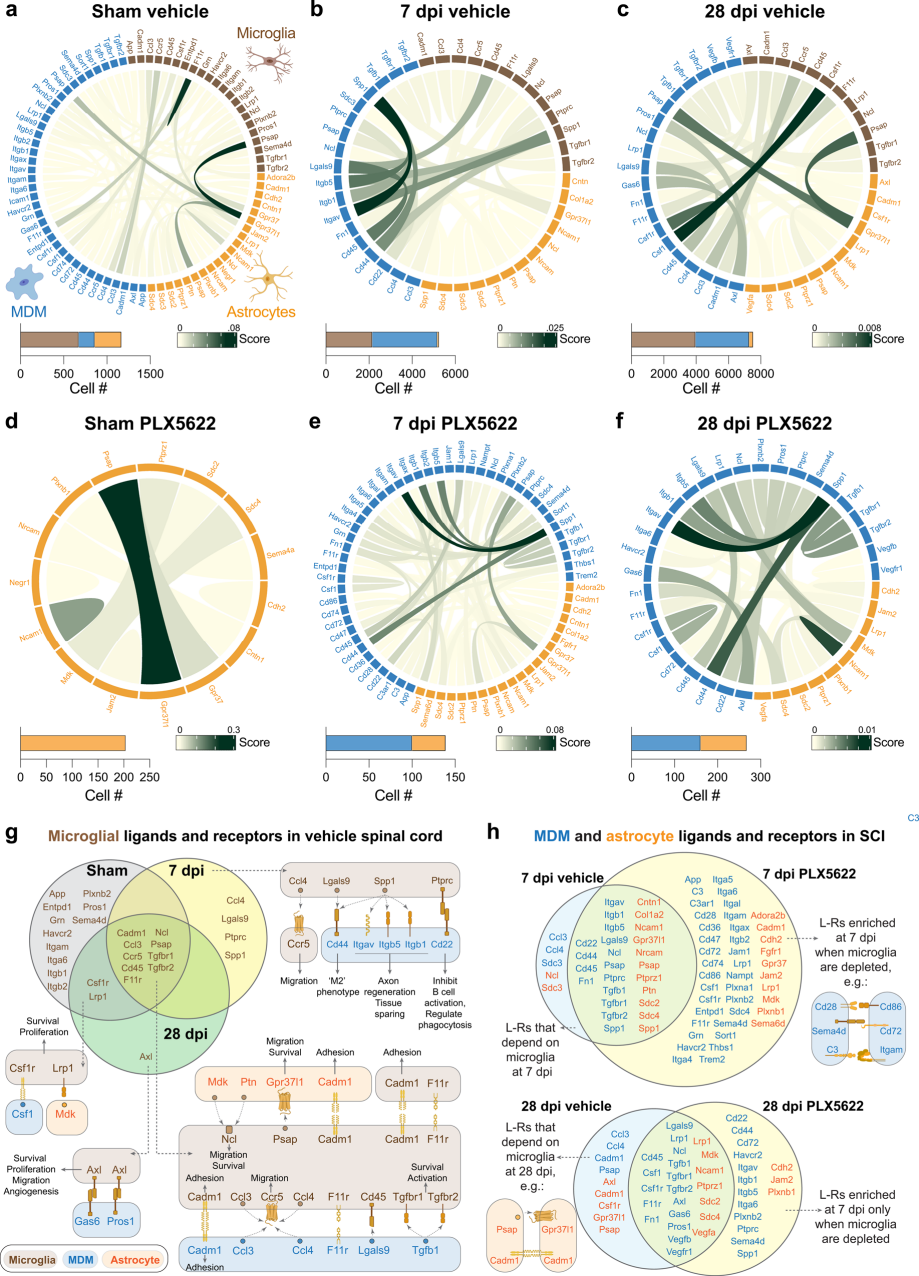
CellChat analysis reveals that microglia-dependent cell-cell communication (CCC) and disruption of MDM and astrocyte CCC following microglia depletion. **a**–**f** Chord plots showing interactions between microglia (brown), MDMs (blue) and astrocytes (orange) in sham, 7 dpi, and 28 dpi spinal cords from vehicle (**a**–**c**) and microglia depleted (**d**–**f**) animals. Note the disorganization in astrocyte/MDM interactions in **e** and **f** vs. **b** and **c**. The strength of the interaction is indicated by the CCC score. The score is defined as the probability of CCCs by integrating gene expression data with prior database knowledge^43^ of the interactions between signaling ligands, receptors, and their cofactors. Each chord plot has been scaled to appreciate the edges depicting CCCs with smaller probabilities. The cell number of each cell type is indicated in the stacked bar graph under each chord plot. **g** Venn diagram showing enriched microglial ligands and receptors (L-R) in sham, 7 dpi and 28 dpi spinal cords. Schematics show how they interact with microglial, MDMs and astrocyte L-Rs in SCI conditions and their functional implications (where known). **h** Venn diagram showing MDM and astrocyte L-Rs enriched in SCI, and the effect of microglia depletion on these interactions. Note the number of additional enriched MDM L-Rs, and alteration of several astrocyte signaling axes, in the absence of microglia. Source data are provided as a Source Data file.

To quantitatively infer and analyze microglia-dependent communication networks after SCI, we computationally isolated the microglia, MDM, and astrocyte clusters from the scRNAseq datasets, then used CellChat^43^ to extract complex signaling patterns involving soluble and membrane-bound ligand-receptor interactions. Unlike other methods (e.g., NicheNet, iTALK), CellChat uses systematically curated classifications of ligand-receptor pairs with functionally related signaling pathways, does not exclude receptors that function as multi-subunit complexes, and considers signaling cofactors and co-receptors that can act as agonists and antagonists. “Chord plots” in Fig. 8 show autocrine and paracrine signaling interactions with color-coded ligand-receptor interaction scores, and the number of significant interactions in each condition.

In the intact spinal cord, a highly scored interaction is microglia Psap (Prosaposin) with astrocyte Gpr37l1 (Fig. 8a, g). Psap signaling via Gpr37l1 protects astrocytes from oxidative stress and death^44^. When microglia are depleted in the intact spinal cord, reduced microglia-dependent Gpr37l1 crosstalk is compensated for in an autocrine manner by Astrocyte Psap (Fig. 8d).

At 7 dpi, prominent signaling networks include microglial Spp1, which interacts with MDM receptors Cd44, Cd45, and the integrins Itgb5, Itgb1, Itgav (Fig. 8b, g). Spp1 encodes for osteopontin, which limits inflammation and can promote axon regeneration^45,46^. At 28 dpi, enriched microglial ligand-receptor (L-R) interactions include Csf1r with MDM Csf1, an axis known to promote SCI recovery^16^ (Fig. 8c, g). The microglial L-Rs consistently enriched in sham, 7, and 28 dpi include cytokines (Ccl3, Ccr5, Tgfbr1/2) that coordinate MDM recruitment, microglial Psap, which activates the neuroprotective and glioprotective astrocyte receptor Gpr37l1, and microglial cell adhesion molecule 1 (Cadm1), which binds astrocyte and MDM Cadm1 to promote cell adhesion (Fig. 8a–c, g). Figure 8g. shows the microglial L-Rs enriched at specific time points after SCI, with schematics showing how they interact with microglial, MDMs, and astrocyte L-Rs in SCI conditions and their functional implications (where known). Interestingly, several of these L-Rs (Ccl4, Ccl3, Csf1r, Ptprc, Tgfbr2, Lgals9) were detected in our bulk RNAseq data as being increased in SCI and failing to increase without microglia present (Fig. 2, Supplemental Table 1).

Figure 8h shows MDM and astrocyte L-Rs enriched in SCI and the effect of microglia depletion on these interactions. At 7 dpi, with microglia depleted, there are dozens of additional MDM L-Rs that are known to promote secondary injury, including: Sema4d-Cd72/integrin, which induces axon growth cone collapse^47^, CD28/CD86 interactions^10^, and CR3/Itgam (Mac-1)^48^ (Fig. 8b, e, h). At 28 dpi, in addition to abolishing microglia Psap interactions with astrocyte Gpr37l1, microglia depletion also disrupts astrocyte autocrine Psap-Gpr37l1 interactions and cadherin-cadherin (Cadm1) interactions important for astrogliosis and cell-cell adhesion (Fig. 8c, f, h). Considered with the scRNA-seq data, these communication maps reveal discrete microglia-dependent signaling networks that are required for tissue repair and homeostasis post-SCI.

## Discussion

Using reporter mice or antibodies that distinguish microglia from MDMs^49,50^ and microglia-specific depletion techniques^51–56^, both beneficial^16,18,57,58^ and detrimental^59,60^ roles for microglia in SCI have been observed. Data herein extend those reports and show that microglia are essential for restoring tissue homeostasis and achieving optimal recovery after SCI. Microglia exert these beneficial effects by regulating the transcriptional fate, function, and intercellular crosstalk between various non-neuronal cell types.

SCI triggers the recruitment of monocytes, which differentiate into MDMs^61^. Infiltrating MDMs persist indefinitely as part of a central fibrotic lesion “core” that is sequestered from surrounding healthy tissue by a reactive glial border. We show a core microglial transcriptional signature or “sensome”, controls this neuroinflammatory cascade after SCI; without microglia, MDM recruitment is delayed and when MDMs finally enter the injured spinal cord, they fail to adhere to or be corralled within the primary lesion. Instead, MDMs invade adjacent white matter where they propagate secondary injury.

Building on recent observations^21,62,63^, we show that functionally heterogeneous intraspinal microglia regulate the production of MDM chemoattractants (e.g., CCL2) and also the expression of MDM genes that control the recognition of DAMPs (e.g., TLR2), lipid-binding, lipid processing, calcium-binding and extracellular matrix proteases (e.g. Mmp12)^64^. Without microglia, a degradative MDM phenotype predominates and exacerbates tissue pathology.

Although we did not try to manipulate all aspects of the microglia-MDM axis to effect more efficient repair, nearly complete rescue of the microglia depletion phenotype was achieved by reconstituting microglia-depleted spinal cords with TLR2 ag and rCCL2. CellChat data revealed that microglia-MDM CCL3/CCL4-CCR5 interactions are key in the acute post-injury period. However, amplifying  chemokine signaling in injured spinal cords with microglia present exacerbates pathology and worsened functional recovery, indicating that hyper-activation of normal microglia-MDM cross-talk can cause pathology. Interestingly, delayed microglia depletion was reported to improve SCI recovery^59,60^, suggesting that excessive or prolonged activation of microglia subsets may be detrimental, although sex-specific effects and use of different CSF1R inhibitors could also explain these observations.

Microglia per se form a border around necrotic tissue^16^. Our data show that microglia also control astroglial border formation around SCI lesions, presumably by regulating the expression of genes that control astrocyte survival, actin polymerization, proliferation and adhesion, and gliosis (e.g. *Apoe*^65^, *Tmsb4x*). Microglia modulate astrocyte fate and function through e.g. Psap-Gpr37l1 and Cadm1-Cadm1, which protect against oxidative stress^44^ and promote proliferation and adhesion to ECM proteins, respectively. Gene changes in astrocyte and NG2 cells that are microglia-dependent (e.g., *Tmsb4x, Il4, and Ntm*) could also control cell proliferation and endogenous repair mechanisms in these cells^23^. CellChat data also revealed that microglia normally produce ligands like osteopontin (Spp1), that can promote CNS regeneration and neuroprotection^45,46,66^.

Potential limitations of our study include the use of only female mice aged 10–12 weeks and possible off-target effects of PLX5622. Young female mice were used exclusively to eliminate morbidity and other logistical challenges associated with performing SCI in male and aged mice^67^. However, since microglia are sexually dimorphic^68^ and their transcriptional phenotype changes with age^69^, male vs. female and young vs. old cohorts should be compared in future studies. Such comparative studies might also have translational value since the SCI population is predominantly male and the median age at the time of injury has increased from 29 in the 1970s to 43 more recently^70^. Also, even though PLX5622 predominantly affects microglia (see Sup. Fig. 2), the drug could have “off-target” effects, including on monocyte-derived macrophages^71^. Despite these limitations, our results provide a robust foundational dataset for examining how biological factors (age, sex, injury level, and severity), environmental variables (diet, enrichment, stress), and potential experimental therapeutics, affect microglia-dependent repair mechanisms after contusion SCI.

In summary, our data reveal a central role for microglia in controlling SCI pathobiology and functional recovery and identify molecules and interactions that should be manipulated in future studies to repair the injured spinal cord.

## Methods

### Mice

All surgical and postoperative care procedures were performed in accordance with The Ohio State University Institutional Animal Care and Use Committee. Adult female (8–10 week old) female C57BL/6 J (WT) mice were purchased from Jackson Laboratories (RRID: ISMR_JAX:000664). Mice were age and weight-matched within experiments. Animals were housed under conventional ventilation conditions on a 12 h light-dark cycle with *ad libitum* access to food and water. Room temperature was between 20–26 °C and humidity was between 30–70%.

### Pharmacological microglia depletion

A colony-stimulating factor 1 receptor (Csf1R) antagonist, PLX5622 (formulated at 1200 mg/kg chow p.o; 290 ppm, Plexxikon) was used to pharmacologically deplete microglia. PLX5622 is an orally bioavailable, blood-brain barrier permeable compound that specifically inhibits Csf1R tyrosine kinase activity with 50-fold selectivity over four related kinases^51^. For all experiments using PLX5622, animals were randomly assigned to cages then cages were randomly assigned to a diet group using QuickCalcs (GraphPad software). Animals within a cage received the same diet. For dosing studies (Sup. Fig. 1a–e, h), uninjured mice received PLX5622 chow continuously for 3, 7, 14, or 21 days. For drug washout studies (Sup. Fig. 1f, g, h), mice received PLX5622 diet for 14 days, followed by 3 or 7 days of vehicle diet. Control mice received vehicle diet for 21 days. For initial contusion SCI studies (35 days post-injury (dpi) endpoint) (Fig. 1, Sup. Fig. 2, Sup. Fig. 3), mice continuously received either vehicle or PLX5622 diet from −14 dpi to 35 dpi. For crush SCI experiments, mice were continuously fed vehicle or PLX5622 from −14 to 14 dpi (L1 crush, Sup. Fig. 4a–r) or −14 to 28 dpi (T9 crush, Sup. Fig. 4s–v). For bulk RNAseq studies (7 dpi endpoint) (Fig. 2), mice received continuous PLX5622 or vehicle diet from −14 to 7 dpi. For SCI timeline studies (7, 14, or 21 dpi endpoint) (Sup. Fig. 5), mice were continuously fed PLX5622 or vehicle diet from −14 to 7 dpi, 14 dpi, or 21 dpi. For single-cell RNAseq studies (7 or 28 dpi endpoint) (Figs. 4–6, Sup. Figs. 6–14), mice were continuously fed PLX5622 or vehicle diet from −14 until the endpoint. For experiments using intraspinal injections, mice were continuously fed vehicle or PLX5622 from −14 to 7 dpi (Sup. Fig. 6) or 35 dpi (Fig. 3). To ensure experimenters were blinded to diet groups, mice were acclimated to diet-free cages before testing, and these cages were coded (e.g. ‘A’, ‘B’ ‘C’) prior to testing. Experimenters were also blinded to diet group for histology, tissue processing, image acquisition, and data analysis by coding the animals and tissue.

### LPS administration

To determine whether PLX5622 affected myeloid lineage cells other than microglia at baseline or following immune challenge, mice were fed vehicle or PLX5622 chow starting two weeks before receiving four consecutive daily i.p. injections of 1 mg/kg LPS (*E. coli*, serotype 055:B5, Sigma) or 0.1 M PBS^72,73^. Body weight was measured before and during LPS challenge to confirm bioactivity of LPS in each treatment group (Sup. Fig. 1p–z’). Mice were anesthetized using 1.5x the surgical dose of anesthetic (see above) 24 h after the last injection for flow cytometry or MethoCult assay (see below).

### Flow cytometry

#### Blood

Blood was collected via cardiac puncture with a 25 G syringe and transferred to blood collection tubes coated with EDTA. A 50 μl sample of whole blood per mouse was used for flow cytometry Sup. Fig. 1q–t).

#### Spleen

The spleen was rapidly dissected, weighed, and placed in a small volume of DMEM (Sup. Fig. 1r, u–w’). Spleens were minced with sterile dissection scissors and mashed through a 40 μm sterile cell filter using the plunger of a 3 ml syringe and rinsed with 10 ml of IMDM.

#### Bone marrow (BM)

Both femurs from each mouse were removed, cleaned, and placed in a small volume of DMEM. Pictures of representative bones were captured using an iPhone 6 s and pseudocolored in ImageJ (Sup. Fig. 1r, x–z**’**). Bone marrow cells were isolated by flushing bones with 10 ml of DMEM through a 40 μm sterile cell filter.

#### Sample processing

Samples were processed as described previously^74^. Briefly, blood, BM, and spleen samples were diluted 1:5 with NH_4_Cl red blood cell lysis buffer (StemCell Technologies, # 7850) and incubated for 5 mins at RT. Cells were centrifuged (300 x g for 4 mins) and resuspended in 0.1 M PBS. Cells were then incubated with 1:100 zombie green viability dye (BioLegend, 423112) for the exclusion of dead cells. Cells were washed then resuspended in flow buffer (0.1 M PBS with 2% FBS) containing rat anti-CD16/32 (1:200; BD Bioscience) for 10 mins on ice to block F_c_ receptors. Cells were then incubated with flow cytometry antibodies (**see Key Resources Table**) for 30 mins on ice, washed, and resuspended in flow buffer. 10 μl of liquid counting beads (BD Biosciences, 335925) were added to allow for quantification of absolute cell numbers (Sup. Fig. 1s, t, v, w, y, z). Samples were processed on a BD Fortessa flow cytometer (BD Biosciences) running FACS Diva software (v9.0) (BD) and analyzed using FlowJo (v10.8.1) (BD). OneComp ebeads (ThermoFisher, #01-1111-41) were used to set voltage intensities and compensation thresholds to remove spectral overlap. Unstained controls, isotype controls, and fluorescence minus one controls were used to identify background staining levels and determine gate placement. Doublets were excluded based on linearity of FSC-A and FSC-H. From singlets, live cells were identified as the Zombie-FITC^lo/-^ population. Neutrophils were designated Ly6G^+^Ly6C^+^ cells and monocytes were designated Ly6G^-^Ly6C^+^ cells. The gating strategy for blood samples (using the Veh + PBS mouse in Sup. Fig. 1r) is shown in Sup. Fig. 1q. This gating strategy was the same for spleen and bone marrow, but using tissue-specific controls to set the gates. CD11b and CD11c staining were also used to confirm cell identity.

### Methocult assay

The MethoCultTM 430 GF M3434 ex vivo culture assay (StemCell Technologies) was used to determine whether treatment with PLX5622 affected the ability of BM and spleen stem cells to differentiate into myeloid lineage cells under baseline conditions and during the immune challenge (Sup. Fig. 1w’, z’). This assay consists of a methocellulose media containing cytokines and growth factors to support the development of cell colonies from single hematopoietic stem/progenitor cells (HSPCs)^74^. After administering 1.5x surgical dose of anesthesia and preparing single-cell suspensions (as described above for flow cytometry), cells were counted with a hemocytometer and 7 500 BM cells were plated, and 200 000 splenocytes were plated. Each sample was cultured in a meniscus-free 6-well SmartDishTM 433 (StemCell Technologies) and placed in an incubator at 37 °C and 5% CO_2_. At 10 days after plating, colonies were quantified by standard inverted light microscopy using a StemGridTM 435 counting underlay (StemCell Technologies). Colonies were classified as blast forming unit-erythrocyte (BFU-E), granulocyte (CFU-G), monocyte (CFU-M), granulocyte/monocyte (CFU-GM), or granulocyte/erythrocyte/monocyte/ megakaryocyte (CFU-GEMM), based on colony composition and cell morphology.

### Spinal cord injury and post-operative care

#### T9 contusion

Mice were anesthetized using a cocktail of ketamine (80 mg/kg; i.p.) and xylazine (10 mg/kg; i.p.). For spinal contusion injuries, the ninth thoracic vertebra (T9) was identified based on anatomical landmarks and the lamina removed. Mice received a moderate (75 kdyne) contusion SCI using the Infinite Horizons impactor (Precision Systems and Instrumentation, LLC.)^75^. There were no differences in injury parameters between experimental groups.

#### L1 crush and T9 crush

For L1 crush, the T11 lamina was removed. A bilateral crush injury was performed at the first lumbar (L1) spinal level by inserting #4 Dumont forceps (Fine Science Tools #11241-30, with tip width narrowing from 0.4–0.2 mm) 2 mm ventrally into the vertebral column on both sides of the spinal cord then laterally compressing the spinal cord for 2 s without breaking the dura^41,76^. Injury level and dura integrity were confirmed at post-mortem dissection and using histology. The same method was applied to crush the spinal cord under vertebral level T9, except here the T9 lamina was removed.

#### Post-operative care

After SCI, the muscle was closed with 5.0 polyglactin dissolvable sutures, and the skin was closed with wound clips. Animals were injected with saline (2 ml, s.q.) and then placed into warmed cages (35 °C) until they recovered from anesthesia. To prevent dehydration and infection, mice were supplemented with daily saline (1-2 ml s.q.) and Gentocin (1 mg/kg s.q.) for the first 5 dpi. Because acutely spinal injured mice cannot rear to reach the food hopper in conventional cages, Vehicle or PLX5622 pellets were also placed in the cages for easy access. Bladders were manually voided twice daily for the duration of the experiments. Body weight and urinary pH were monitored weekly.

### Axon tracing

To trace corticospinal (CST) axons in mice with a T9 crush SCI (Sup. Fig. 4s–v’), mice were anesthetized with ketamine (80 mg/kg; i.p.) and xylazine (10 mg/kg; i.p.). A rectangular cranial opening (1.5 mm × 1 mm) was created above the sensorimotor cortex (Bregma: −0.3 to −1.8 mm, lateral: 1 mm to 2 mm) using a pneumatic drill and fine forceps. 1 μl (total) of biotinylated dextran amine (BDA; 10%) was injected at a depth of 0.6 mm into the sensorimotor cortex at three sites in the window area using a Nanoliter 2000 system. BDA injections were performed at 14 dpi, a time when CST axons are undergoing axon dieback and activated macrophages are evident in the degenerating CST^29^. After injection, the head was sutured closed and animals received 2 ml of saline s.c. and were kept on warmers (37 °C) until anesthesia recovery.

### BrdU

To label proliferating cells, mice were injected with the thymidine analog, 5-bromo-2′-deoxyuridine (BrdU) (50 mg/kg i.p. in 0.9% saline; Sigma-Aldrich #10280879001) daily from 1–7 dpi.

### Behavior

#### Open field locomotion

Two investigators, blind to the experimental condition, assessed mouse hindlimb function in open-field using the Basso Mouse Scale (BMS) for locomotion^77^. BMS testing was performed at −1 (baseline), 1, 3, 7, 10, and 14 dpi, and weekly thereafter until the experimental endpoint. BMS subscores were also assigned to quantify finer aspects of locomotion.

#### Horizontal ladder task

Three days before SCI, mice were trained to walk along a horizontal ladder as described previously^78^. This task requires mice to navigate across a horizontal ladder with rungs spaced 1.25 cm apart for 40 cm with a mirror below. Mice were video recorded from the side view and the underside view during the task. Mice were motivated to walk along the ladder by placing home cage bedding at the end. A paw falling below the rungs of the ladder during a step in the forward direction was counted as one mistake. The total number of mistakes was averaged across three trials per mouse. Baseline data were recorded beginning 2 d pre-SCI. SCI mice were tested on the horizontal ladder task at 3 weeks post-SCI, and then weekly thereafter until the experimental endpoint.

#### Mechanical allodynia

Mechanical sensitivity was assessed by measuring threshold responses to a calibrated von Frey rigid tip (IITC Life Science) as described previously^79^ (Sup. Fig. 1m). Mice were placed on a mesh platform in a clear compartment (8 cm × 12 cm × 5.5 cm) that allows unrestrained exploration, locomotion, and grooming. Animals acclimated to the testing environment for 30 mins before testing. The mid-line of the plantar surface of the left and right hind paws was probed three times with progressively increasing force intensities to determine the force that repeatedly elicits withdrawal of the hind paw from the calibrated rigid tip. The log_10_stimulus intensity (mg*10) was the force required to elicit consistent withdrawal of the hind paw. Measurements were taken on three different days and the data averaged over the three trials.

#### Thermal sensitivity

A hot plate task was used to assess thermal sensitivity^80^ (Sup. Fig. 1n, o). Briefly, mice were placed in a clear compartment (8 cm × 12 cm × 5.5 cm) that allows unrestrained exploration, locomotion, and grooming on a hot plate set to 25 °C. Mice were allowed to acclimate for 30 mins. The heat was set to increase to 50 °C over a period of 4 mins. The time and temperature at which mice reflexively raised one or both hind paws was recorded, at which time the mice were removed from the hot plate.

### Tissue processing

Mice were anesthetized with a lethal mixture of ketamine/xylazine (1.5 x surgical dose) then transcardially perfused with 0.1 M PBS followed by 4% paraformaldehyde. Spinal cords were removed, post-fixed in 4% PFA for 2 h then transferred to 0.1 M PBS overnight before being cryoprotected by incubation in 30% sucrose for 48 h. Images of representative spinal cords were captured on an iPhone 6 s (Sup. Fig. 5a). Spinal cords were blocked into 10 mm segments centered on the impact site with the dorsal columns facing up, then embedded in Tissue-Tek optimal cutting temperature medium (VWR International), and rapidly frozen on dry ice. Tissue sections were cut in series (10 μm thick, 10 slides per series) using a Microm cryostat (HM 505 E) and collected on SuperFrost Plus slides (Thermo Fisher Scientific). Contused thoracic spinal cord segments were cut along the coronal (rostral-caudal) axis. Crushed thoracolumbar spinal cord segments were cut along the horizontal (dorsal-ventral) axis. Slides were stored at −20 °C until immunostaining.

### Immunostaining

#### Fluorescence immunolabeling

Slides were dried at room temperature (RT) for 2 h, rinsed (0.1 M PBS) then blocked with 4% bovine serum albumin (BSA) and 0.3% Triton X-100 (BP^3+^) in PBS for 1 h at RT. Sections were incubated overnight with primary antibodies (**Key Resources Table**) at 4 °C in humidified chambers. After washing in 0.1 M PBS (3 ×4 mins), sections were incubated with secondary antibodies (**Key Resources Table**) diluted in 4% bovine serum albumin and 0.1% Triton X-100 (BP^+^) in PBS. Sections were washed again in PBS (3 × 4 mins), then coverslipped with Immumount (Thermo Fisher Scientific). For BrdU labeling, after the initial rinse but before blocking, slides were incubated with 2 N HCl for 25 mins at 37 °C.

#### Immunoperoxidase labeling

After the initial rinse slides were incubated at RT in methanol containing 6% H_2_O_2_ for 30 mins to quench endogenous peroxidase activity. After washing in 0.1 M PBS (3 ×4 mins), blocking and primary antibody incubations were performed as above. Sections were then incubated with biotinylated secondary antibodies for 1 h at RT. Bound antibody was visualized using Elite-ABC reagent (Vector laboratories) with ImPACT diaminobenzidine as a substrate (Vector Laboratories Cat #SK-4105). Sections were dehydrated through sequential 2 min incubations in 70%, 70%, 90%, and 100% ethanol solutions, followed by 3 ×2 min incubations in Histoclear. Slides were coverslipped with Permount (Thermo Fisher Scientific).

To visualize myelin, following immunoperoxidase development of neurofilament, slides were rinsed in dH_2_O and immersed in acetone for 8 mins. Samples were then rinsed in dH_2_O before incubation in eriochrome cyanine (EC) for 30 min at RT. Slides were washed in dH_2_O and differentiated in 5% iron alum and borax ferricyanide for 5–10 mins before dehydration and coverslipping. The injury epicenter in contusion SCI was defined visually as the section with the smallest visible rim of spared myelin.

### Pathology

#### General pathology

EC/neurofilament stained sections were digitized using a ScanScope XT scanner (Aperio; OSU Comparative Pathology and Mouse Phenotyping Shared Resource; College of Veterinary Medicine) and ImageScope software (Leica Biosystems). Lesion analysis was performed in ImageJ by inspecting sections 0.2 mm apart across a 4 mm segment extending from 2 mm rostral to 2 mm caudal from the lesion epicenter. Lesioned tissue within this region, delineated by neurofilament staining, was outlined in ImageJ using the polygon selection tool. Lesion volume was calculated by: Σ (area of lesioned tissue per section x distance between sections). Lesion length was the rostral-caudal distance spanned by sections containing lesioned tissue. Three-dimensional reconstructions of contusion SCI lesions were generated with M3D from MCID Elite Software. For lesion analysis in contusion SCI, spared myelin and/or spared neurofilament were outlined in ImageJ using the polygon selection tool, and expressed as a percentage of the section area. For crush SCI, the epicenter was defined as the horizontal section at the level of the central canal; the area of spared myelin was expressed as a percentage of EC^+^ tissue in a 2 mm-length area of tissue centered on the crush site.

#### Microglia

For contusion SCI, sections 0.2 mm apart extending from 2 mm rostral to 2 mm caudal to the epicenter were stained for P2RY12 and developed using immunoperoxidase staining. Sections were digitized using a ScanScope XT scanner (Aperio) and ImageScope (Leica Biosystems). For contusion SCI, the number of P2RY12^+^ cells was counted using Imaris and expressed as a percentage of the section area, which was outlined in ImageJ. For crush SCI, the area of microglia was expressed as a percentage of P2RY12^+^ tissue in a 2 mm-length area of tissue centered on the crush site.

#### MDMs

For contusion SCI, spinal sections 0.2 mm apart extending from 2 mm rostral to 2 mm caudal to the epicenter were stained for P2RY12 and the pan macrophage marker F4/80. Sections were imaged using a Leica TCS SP8 confocal microscope. Because MDMs are difficult to individually differentiate within the central lesion core at 35 dpi, the abundance of intraspinal P2RY12^-^F4/80^+^ MDMs in whole spinal cord sections was expressed as a percentage of the section area. For epicenter region of interest (ROI) analysis, 0.1 mm^2^ boxes were placed in the center of the section and over the ventromedial white matter. The area of P2RY12^-^F4/80^+^ staining in each ROI was selected using the threshold tool in ImageJ and expressed as a percentage of the ROI area. To assess the size of phagocytically active MDMs in remaining ventral white matter, sections were immunostained with P2RY12, CD68, and myelin basic protein (MBP). Images were captured using a Leica TCS SP8 confocal microscope. The spared ventral white matter was manually outlined as accurately as possible, and the surface area of individual P2RY12^-^CD68^+^ cells in this region were quantified using Imaris (total of 177–395 cells per animal). The average cell area in this region was calculated for each animal. For crush SCI, the area of F4/80^+^ cells in horizontal sections at the lesion epicenter were manually outlined in ImageJ, and expressed as a percentage of F8/80^+^ tissue in a 2 mm-length area of tissue centered on the crush site.

#### Cell proliferation

Sections were immunostained for GFAP, NG2 and BrdU then imaged using a Leica TCS SP8 confocal microscope. GFAP^+^BrdU^+^ and NG2^+^Brdu^+^ double-labeled cells were counted 0.6 mm rostral and caudal to the epicenter and at the epicenter using Imaris v8.0 (Bitplane Scientific Software), or only at the epicenter section for crush SCI. For this, GFAP/BrdU and NG2/BrdU co-localization channels were built based on absolute staining intensity, and touching objects were split. The channel was converted into a surface and thresholds set based on cell area and encapsulation of a BrdU^+^ cell nucleus by GFAP or NG2. NG2^+^ macrophages were excluded using morphological criteria^81^.

#### GFAP intensity analysis

To quantify the intensity of GFAP labeling, horizontal sections from mice with crush SCI were immunostained for GFAP. Sections were imaged using a Leica TCS SP8 confocal microscope and converted to 8-bit format. With the rostral side oriented left, a rectangular box centered over the crush site was drawn on the image. The box was 1.5 mm wide along the rostral-caudal axis and as high as the pia mater; care was taken not to include background pixels. The grayscale profile along the horizontal axis was plotted in ImageJ and the data was exported to Microsoft Excel.

#### Neurons

To estimate the number of neurons in horizontal sections of crushed spinal cords, sections were immunostained for neuronal nuclei (NeuN). Four, 500 μm wide regions of interest (ROI) were overlaid on the section (ROI #1–4), with ROI 1 starting at the lesion edge which was delimited by dense astrogliosis. ROI #2–4 started at the end of the previous ROI, covering a total distance of 2 mm from the lesion edge. NeuN^+^ cell bodies in each ROI were manually counted.

#### Lipid phagocytosis

Oil Red O stain was used to measure phagocytosed lipid debris. Sections were rinsed in dH_2_O and 70% ethanol each for 2 min before being placed in a saturated solution of Oil Red O (catalog # O0625-25G; Sigma-Aldrich) dissolved in 70% ethanol at 60 °C for 30 min. The tissue was differentiated in ethanol, washed in distilled water, and then coverslipped with Immumount (Thermo Fisher Scientific). The area and number of phagocytosed lipid clusters were manually quantified in ImageJ.

#### Axon dieback

Tiled montages of horizontal sections stained for BDA, GFAP, and F4/80 (depth of ~280 μm) were captured using a Leica TCS SP8 confocal microscope. Retraction of BDA^+^ dorsal CST axons from the T9 crush lesion edge (identified by GFAP labeling), was measured in one horizontal section per mouse in ImageJ. Higher magnification images of BDA + axons were also captured.

### MDM reprograming

Pilot studies were performed to optimize the dose and route of administration of MDM-reprograming factors that yields a robust MDM response in the microglia-depleted injured spinal cord. At 2 dpi, mice were injected intraspinally with 1 μl of sterile saline containing 50 ng of recombinant mouse Ccl2 (rCcl2, R&D systems, 479-JE-05) and/or 500 ng of a Tlr2 agonist (Pam2CSK4, tlrl-pm2s-1 InvivoGen; Tlr2 ag)^29^ at a rate of 300 nl/min using a Nanoliter 2000 system. These times were chosen based on the peaks of endogenous intraspinal Ccl2^27^ and Tlr2 expression^28^. To target the central gray matter, stereotaxic injections were made at a depth of ~0.6 mm at the midline in the center of the injury. After microinjection, muscle layers were sutured with 4-0 nylon suture and the skin was closed with surgical staples. Post-operatively, animals received 1 ml of subcutaneous saline, were kept warm on a heating plate during recovery from anesthesia, with free access to food and water. At 3 dpi, mice were also injected i.p. with Tlr2 ag (50 μg/kg i.p.) or sterile PBS. Pilot animals were perfused at 7 dpi. Pilot studies determined that injections of rCCL2 i.s., Tlr2 ag i.s. and i.p. elicit an immune response in microglia depleted animals that is the most similar to the normal inflammatory response (compare column 1 vs. column 7 in Sup. Fig. 6b, c), so this combination was chosen for the 35 dpi study.

For the 35 dpi study, mice received vehicle or PLX5622 diet from −14–35 dpi and either rCCL2 and Tlr2 ag i.s. (or saline) at 2 dpi and Tlr2 ag i.p. (or PBS) at 3 dpi. Mice received a 75 kdyne contusion SCI as described above. Post-operative care, behavioral analysis (BMS and horizontal ladder), and pathology were performed as described above.

### Bulk RNA sequencing

#### Groups

Female mice (*n* = 34 per group) were fed Vehicle or PLX5622 (1200 mg/kg chow) from −14 to 7 days post-surgery. Mice in each diet group received either a T9 laminectomy (sham) or 75 kdyne T9 contusion SCI.

#### Tissue dissociation

Mice were euthanized with 1.5x the surgical dose of ketamine and xylazine then perfused transcardially with DEPC buffer until the liver and blood were cleared. A 1 cm piece of spinal cord (centered on the T9 laminectomy site) was quickly dissected and homogenized in 1 ml of TRIzol reagent (ThermoFisher). RNA was extracted according to the manufacturers instructions. Briefly, the tissue lysate was centrifuged at 4 °C for 5 mins at 12,000 g. The supernatant was incubated for 5 mins to dissociate nucleoproteins. Samples were incubated with 0.2 ml of chloroform for 2.5 mins then shaken vigorously for 15 sec. Samples were centrifuged at 4 °C for 15 mins at 12 000 g. The aqueous phase was collected and mixed with 0.5 ml of isopropanol with 2 ul of glycogen then stored at −20 °C. Samples were centrifuged (4 °C, 5 mins, 12,000 g) and the pellet was resuspended in 75% ethanol. The sample was vortexed, centrifuged (4 °C, 5 mins, 7500 g), the supernatant removed and pellet dried for 5–10 mins. The pellet was resuspended in 20 μl RNAse-free water and warmed to 55 °C. RNA yield was determined using Nanodrop. RNA was purified using the QIAGEN RNeasy spin column steps according to manufacturers instructions. RNA quality and final yield was then measured using a RNA 6000 NanoAssay by Chip. Samples with RIN > 7 were included for analysis (average RIN = 8.6). Samples were stored at −80 °C until sequencing.

#### Sequencing and analysis

RNA-seq was carried out by the UCLA Technology Center for Genomics and Bioinformatics. In brief, the library was prepared using Nugen Ovation RNA Ultra Low Input (500 pg) + Kapa Hyper. The quantity and quality of cDNA were assessed using an Agilent 2100 Expert High Sensitivity DNA Assay. cDNA samples were sequenced at UCLA Technology Center for Genomics and Bioinformatics. Reads were aligned to the mouse GRCm38 reference genome using STAR (v.2.4.0). Read counts for RefSeq genes (mm10) were generated by HTSeq v.0.6.1. Low-count genes were filtered and fragments per kilobase per million mapped reads (FPKM) values were generated. In total, 17,939 genes were identified. Differentially expressed genes were identified using the DESeq2 package in R Studio (v3.6.3) and verified using Gene Network Analyst 3.0^82^. Significantly increased or decreased genes had an adjusted *p* value of <0.05 and a LogFC cutoff of < −1 (decreased) or >1 (increased) (https://www.networkanalyst.ca/). GO analysis was performed separately for up- and downregulated gene lists using Web-based Gene Set Analysis Toolkit (WebGestalt), with the Gene Ontology Reference Genome set as the reference gene list^25^. The Bioinformatics and Evolutionary Genomics server (http://bioinformatics.psb.ugent.be/webtools/Venn/, Ghent University) was used to create gene Venn diagram in Fig. 2k. Genemania was used to construct the network diagrams (Fig. 2l–n), with co-expression and predicted functional interactions selected as linkages.

### Single-cell RNA sequencing

#### Groups

Mice were fed Vehicle or PLX5622 (1200 mg/kg chow) from two weeks before surgery until the experimental endpoint of 7 or 28 days post-surgery. Mice received either a T9 laminectomy (sham) or 75 kdyne T9 contusion SCI. Samples from 3-4 mice in the same group were pooled to yield data representative of the group.

#### Tissue dissociation

Mice were euthanized with 1.5x the surgical dose of ketamine and xylazine. A 1 cm piece of unperfused, fresh spinal cords (centered on the T9 laminectomy site) was quickly dissected. Tissue was dissociated to a single cell suspension using a gentleMACS dissociator (Miltenyi) and mouse adult brain dissociation kit (Miltenyi, 130-107-677). Dissociated cells were passed through a sterile 70 μm filter to remove large particulate debris. Myelin debris was removed using myelin removal beads (Miltenyi, 130-096-733). Dead cells were removed using Viahance dead cell removal (BioPAL, #CP-50VQ02). Cells were diluted to 1000 cells/μl. This method optimized cell viability, allows comparison of leukocyte profiles present in both sham and SCI conditions, and enriches for resident glia and peripheral immune cells but does not enrich for neuronal populations^83^.

#### Sequencing

The single-cell suspension and Chromium Single Cell 3’ V3 reagents (10X Genomics) were loaded into chips to collect individual cells encapsulated by gel beads in emulsion (GEMs) using the 10X Chromium Controller. cDNA and library amplification for an optimal signal was 13–16 cycles. Sequencing was conducted on HiSeq4000 (Novogene). Fastq sequence files were de-multiplexed, aligned, and annotated using the mouse ENSMBL database and Cell Ranger v3.0 software. Gene expression was counted using unique molecular identifier barcodes, and gene-cell matrices were constructed.

#### Analysis

Data processing and visualizations of the scRNA-seq data were performed using the Seurat package (v.3.2.0) in R (3.6.3)^84^. Our initial dataset contained 23,696 cells with data for 24,062 genes in total. For the initial quality control filtering, we removed individual cells that having greater than 20% mitochondrial RNA, cells detecting less than 200 genes or more than 3,000 genes, and genes that were detected in less than three cells. After applying the quality control criteria, 21,016 single cells and 16,003 genes remained and were included in downstream analyses. Data were scaled to 10,000 transcripts per cell, and transformed to log space. The canonical correlation analysis (CCA) integration method of Seurat was used to correct batch effects. Highly variable genes in the dataset were computed based on dispersion and mean. Principal component analysis (PCA) was performed on the top 2000 variable genes. The top 30 PCs were used to build a k-nearest-neighbors cell–cell graph with k = 20 neighbors. Clusters were identified by the use of the Louvain graph-clustering algorithm with a resolution set to 0.15. The entire dataset was projected onto the two-dimensional space using uniform manifold approximation and projection (UMAP) dimensionality reduction on the top 30 principal components. For each cell cluster, we assigned the cell-type labels using statistical enrichment for sets of marker genes, and manual evaluation of gene expression for small sets of known marker genes. For example, the major cluster designated “microglia” had enriched expression of *P2ry12, Siglech, and Tmem119*, whereas the major cluster designated “MDMs” was enriched for *Gpnmb, Spp1, and Fabp5*^49,83,85–90^. The sub-clustering within cell types was performed based on standard scree plot methods to determine the variability accounted for by each PC. Although some cell clusters (e.g. astrocytes, endothelial cells) contained relatively few cells, a bootstrap analysis, extrapolating to 10,000 cells, confirmed the robustness of clustering and our conclusions. We computationally selected individual cell types for between-group analysis. Significance of difference in violin/scatter plots was determined using a Wilcoxon Rank Sum test with Bonferroni correction. Significantly increased or decreased genes had an adjusted p value of < 0.05 and the Seurat command “logfcthreshold” was 0.176 (1.5-fold change). DEG lists esported as.csv files were entered into GraphPad Prism for creation of volcano plots. Functional gene set enrichment analysis to identify gene ontology terms was performed using Web-based Gene Set Analysis Toolkit (WebGestalt), with the Gene Ontology Reference Genome set as the reference gene list^25^.

### Prediction and quantification of cell-cell communications using CellChat

The cell-cell communications were predicted using the R package CellChat v 1.0.0^43^. Specifically, we isolated the microglia, MDM, and astrocyte clusters from our scRNA-seq datasets within each of the six experimental conditions, i.e., Control Sham, Control 7 dpi, Control 28 dpi, PLX5622 Sham, PLX5622 7 dpi, and PLX5622 28 dpi. Library-size normalization and log-transformation with a pseudocount 1 were performed on each gene expression matrix by the normalizeData function in CellChat. We then loaded a gene expression matrix and its associated cell meta data to create a CellChat object for each of the six experimental conditions using the createCellChat function. We set the ligand-receptor interaction database as CellChatDB.mouse and filtered genes in each CellChat object by retaining the signaling genes to reduce computational load. Overexpressed genes and overexpressed ligand-receptor interactions were identified using the identifyOverExpressedGenes and identifyOverExpressedInteractions functions. Then, we calculated the cell-cell communication probability by the computeCommunProb function. Finally, we filtered out the cell-cell communications if only a few cells (10 by default in CellChat) were in certain cell types. The cell-cell communications among different cell types within each experimental condition were visualized using the Chord diagrams.

### Trajectory inference

To better understand the trajectory of microglia and MDMs after SCI, these objects were extracted then merged in Seurat v4.1.1. Based on the expression matrix and metadata in the Seurat object, a new Monocle object was created in Monocle 3 v 1.0.0^91^. PCA dimension reduction and batch effect removal were performed. UMAP embeddings were generated, with the minimum distance set to 0.1 and 20 set as the number of neighbors. Cells were clustered and the trajectory graph learned. Root cells were determined, and all cells were ordered based on the root cells. Trajectory plots were generated with labels pseudotime, cell types, samples, and marker genes. For prediction of ‘intermediate cells’ (CNS macrophages), the pseudotime value was extracted for each cell, and density curves were generated in ggpubr v0.4.0 for microglia and MDMs, respectively, in the same plot. Based on density curves, we regard pseudotime with the lowest density between 10 and 20 as intermediate cells. Please note that values of the y-axis in the density plot indicate the estimated density instead of the occupied proportion of the two cell types. Data, code and plots are available at https://github.com/OSU-BMBL/Spinal-cord-scRNAseq.

### Statistics and reproducibility

GraphPad Prism (v 8.0.2) was used for general data visualization and statistical analyses. BMS and horizontal ladder data were analyzed using two-way repeated-measures ANOVA with Bonferroni post-hoc tests. Two-sided Student’s t-tests were used to compare differences in one variable between two groups, One-way ANOVA with Bonferroni post-hoc tests were used to compare one variable between three or more groups. Two-way ANOVA with Bonferroni post-hoc tests was used to compare two variables between two or more groups. Linear regression was used to determine the relationship between tissue pathology and functional recovery. All data are shown as mean and SEM, with statistical significance, determined at *p* < 0.05. Sample sizes were determined a priori from historical data using behavioral measures as the primary outcome, with power (1-β) set to 0.8 and α = 0.05. Bulk and single-cell RNA sequencing experiments used groups of mice with sample sizes determined to be sufficient by previous studies^18^. Data in Fig. 1 were successfully reproduced in Fig. 3 and in Sup. Fig. 5, and in two different SCI models in Sup. Fig. 4. Data in Fig. 3 (rCCL2 and TLR2 ag) were successfully reproduced in Sup. Fig.`Yyyyy 6. Microglia depletion data **in** Sup. Figs. 1, 2, and 5 were successfully reproduced twice in quality control experiments for each batch of PLX5622 diet received. Data in Sup. Fig. 2 were successfully reproduced in Sup. Fig. 5. Bulk and single-cell RNA sequencing experiments in Figs. 2, 4–8 and Sup. Figs. 7–15 used 3-4 mice per group and were not replicated due to cost and logistics. Figures were prepared in Adobe Illustrator (v 25.2.3) and graphical images created with Biorender.com.

### Reporting summary

Further information on research design is available in the Nature Research Reporting Summary linked to this article.

## Supplementary information

Supplementary Information

Peer Review File

Reporting Summary

## Data Availability

Source data are provided with this paper and deposited to the Open Data Commons for Spinal Cord Injury (ODC-SCI) Accession: 695, 10.34945/F5B012, Gene expression data for bulk and single-cell RNA sequencing experiments are available on Gene Expression Omnibus (GEO) (GSE196928).

## Source data

Source Data

## Code availability

The R code used for data analysis is available on GitHub at https://github.com/OSU-BMBL/Spinal-cord-scRNAseq with the DOI code on Zenodo: 10.5281/zenodo.659055210.5281/ZENODO.6590552.
