# Supplementary material for: Microglia coordinate cellular interactions during spinal cord repair in mice
Source: Nat Commun. 2022 Jul 14;13:4096. doi: 10.1038/s41467-022-31797-0 (PMC9283484; doi:10.1038/s41467-022-31797-0)
Supplement: Supplementary file 1 — Supplementary Information [file 41467_2022_31797_MOESM1_ESM.pdf]

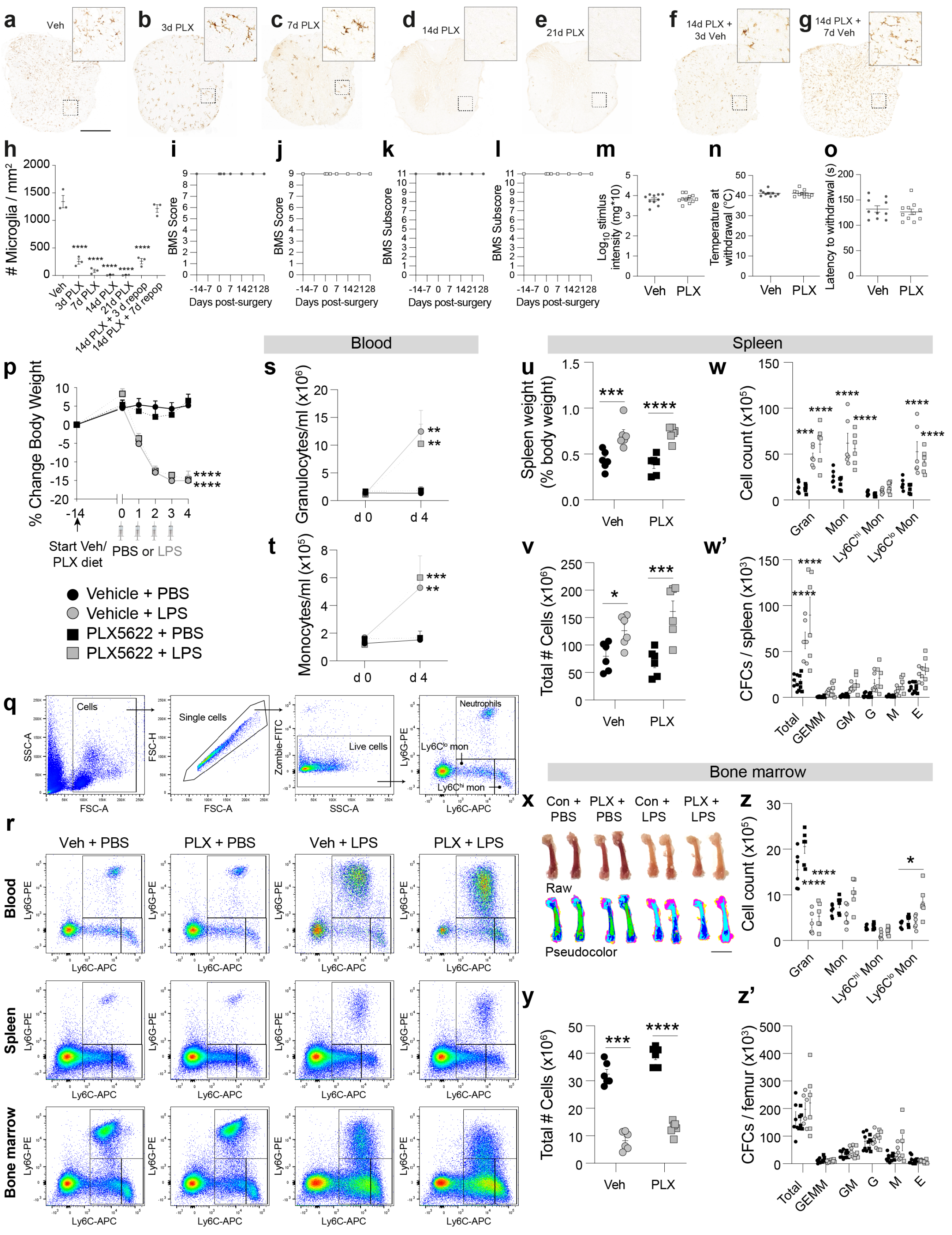

a Veh
b 3d PLX
c 7d PLX
d 14d PLX
e 21d PLX
f 14d PLX + 3d Veh
g 14d PLX + 7d Veh
h # Microglia / mm²
Veh
3d PLX
7d PLX
14d PLX
21d PLX
14d PLX + 3 d repop
14d PLX + 7d repop
i BMS Score
Days post-surgery
j BMS Score
Days post-surgery
k BMS Subscore
Days post-surgery
l BMS Subscore
Days post-surgery
m Log10 stimulus intensity (mg*10)
Veh PLX
n Temperature at withdrawal (°C)
Veh PLX
o Latency to withdrawal (s)
Veh PLX
Blood
Spleen
p % Change Body Weight
Start Veh/ PLX diet
PBS or LPS
s Granulocytes/ml (x10⁶)
d 0 d 4
t Monocytes/ml (x10⁵)
d 0 d 4
u Spleen weight (% body weight)
Veh PLX
v Total # Cells (x10⁶)
Veh PLX
w Cell count (x10⁵)
Gran Mon Ly6Chi Mon Ly6Clo Mon
w' CFCs / spleen (x10³)
Total GEMM GM G M E
Vehicle + PBS
Vehicle + LPS
PLX5622 + PBS
PLX5622 + LPS
q
Cells
Single cells
Live cells
Neutrophils
Ly6Clo mon
Ly6Chi mon
SSC-A FSC-A
FSC-H FSC-A
Zombie-FITC SSC-A
Ly6G-PE Ly6C-APC
r
Veh + PBS
PLX + PBS
Veh + LPS
PLX + LPS
Blood
Spleen
Bone marrow
Ly6G-PE
Ly6C-APC
Bone marrow
x
Con + PBS
PLX + PBS
Con + LPS
PLX + LPS
Raw
Pseudocolor
z Cell count (x10⁵)
Gran Mon Ly6Chi Mon Ly6Clo Mon
y Total # Cells (x10⁶)
Veh PLX
z' CFCs / femur (x10³)
Total GEMM GM G M E

Supplemental Figure 1: PLX5622 depletes microglia. a-e: Representative thoracic (T7) spinal cord sections stained for P2RY12 after feeding vehicle diet (**a**), PLX5622 for 3 d (**b**), 7 d (**c**), 14 d (**d**), or 21 d (**e**) and microglia quantification (**h**). Scale bar (in **a**) = 500 μm. **h**: One-Way ANOVA with Bonferroni post-hoc tests comparing each group to the Vehicle group; n=3 mice per group; mean + SEM ****p<0.0001. **i-o**: Continuous feeding of PLX5622 chow for six weeks (-14-28d) does not impair motor function or sensory function in sham-operated mice as measured by open field (BMS) testing (**i-l**), Von Frey hair testing (p=0.73) (**m**) and hot plate testing (**n,** p=0.46, **o,** p=0.96) **i-l**: Two-Way ANOVA, n=6 mice/group. **m-o**: Student's two-sided t tests, n=9-11 mice/group; mean ± SEM. **p-z**: PLX5622 diet does not affect innate immune cells at rest or in response to LPS. **p**: Mice were continuously fed vehicle or PLX5622 diet for two weeks then challenged with 4 consecutive daily doses of LPS (LPSx4) or PBS. All LPS-injected mice lost ~15% body weight. Blood was collected prior to injections and after the fourth injection. **q, r**: Gating strategy (**q**) and representative plots (**r**) for data in **s, t, v, w, y, z**. Mobilization of granulocytes (**s**) and monocytes (**t**) into the blood after LPSx4. **u-w**: Mice have splenomegaly (**U**), increased total splenocytes (**v**) and blood leukocytes (**w**) after LPSx4 regardless of diet. **w':** The colony-forming cell (CFC) assay showed that splenic hematopoiesis is unaffected by PLX5622. **x, y**: LPSx4 reduces bone marrow cellularity regardless of diet. Scale bar (**x**) = 0.5 cm. **z, z'**: PLX5622 increases the number of Ly6c^{lo} monocytes retained in the BM following LPSx4 challenge (**z**) but does not affect bone marrow hematopoiesis in CFC assay (**z'**). **p, s, t**: Two-way repeated measures ANOVA with Tukey post-hoc tests; **u-w', x-z'**: Two-way ANOVA with Tukey post-hoc tests; n=6 mice/group, *p<0.05, **p<0.01, ***p<0.001, ****p<0.0001, mean ± SEM. **w', z'**: E=Burst-forming unit-erythroid; G=Colony-forming unit-granulocyte; GEMM=Colony-forming unit-granulocyte, erythrocyte, macrophage, megakaryocyte; GM=Colony-forming unit-granulocyte-macrophage. **Related to Figures 1-8.**

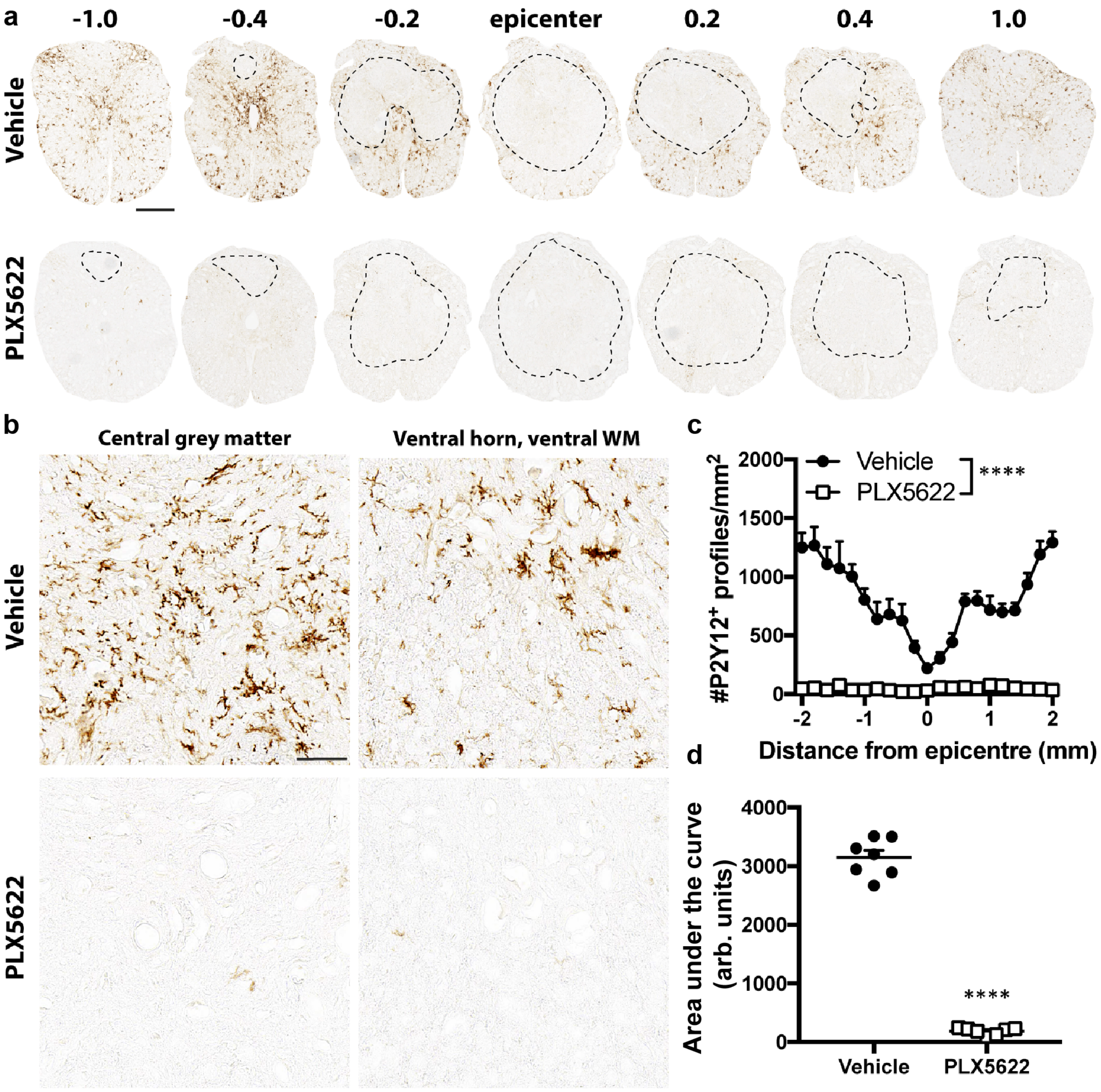

Supplemental Figure 2: PLX5622 depletes microglia in SCI. a: At 35 dpi, intraspinal microglia were identified by immunostaining for P2RY12 (brown). Rostral is left. The frank lesion core is lineated by a black dotted line. In mice fed vehicle (top row), P2RY12+ cells were observed at the lesion margins and in the spared white matter, decreasing in abundance toward the epicenter. In mice fed PLX5622 (bottom row), P2RY12+ cells were rarely observed. Scale bar = 300 µm. **b**: High power magnification showing the morphology of P2RY12+ cells 2 mm rostral to the lesion epicenter in each group. Microglia within 2 mm of the lesion epicenter adopt an amoeboid-shaped morphology. Scale bar = 50 µm. **c, d**: Quantification (**c**) and area under the curve analysis (**d**) confirmed robust depletion of microglia in mice fed PLX5622. **c**: Two-way ANOVA with Bonferroni post-hoc tests; **d**: Student's two-sided t test; n=7 mice per group; mean + SEM; ****p<0.0001. **Related to Figure 1.**

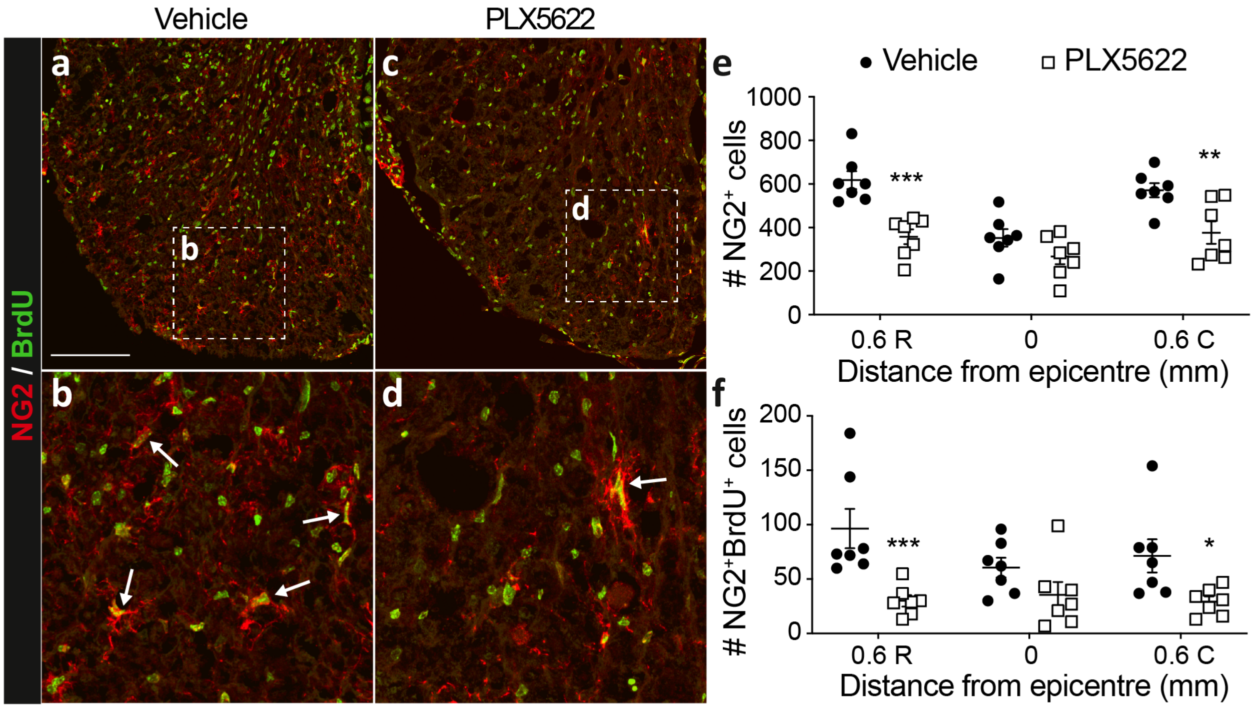

Supplemental Figure 3: Microglia depletion reduces NG2^{+} glia presence and proliferation. a-d: Representative images of NG2 and BrdU co-staining in the white matter 0.6 mm rostral to the lesion epicenter. White arrows point to examples of proliferating NG2 glia. Scale bar = 100 µm. **e, f**: Quantification of total NG2^{+} cell counts per section (**e**) and NG2^{+}BrdU^{+} cell counts per section (**f**) shows fewer numbers of total and proliferating NG2 glia rostral and caudal to the lesion epicenter in mice with no microglia. R, rostral, C, caudal. **e, f**: Two-way ANOVA with Bonferroni post-hoc tests; n=7 per group; mean ± SEM; *p<0.05; **p<0.01; ***p<0.001. **e**: 0.6 mm Rostral, p=0.0001, 0.6 mm caudal, p=0.0043, **f**: 0.6 mm rostral, p=0.0009, 0.6mm caudal, p=0.0487. **Related to Figure 1.**

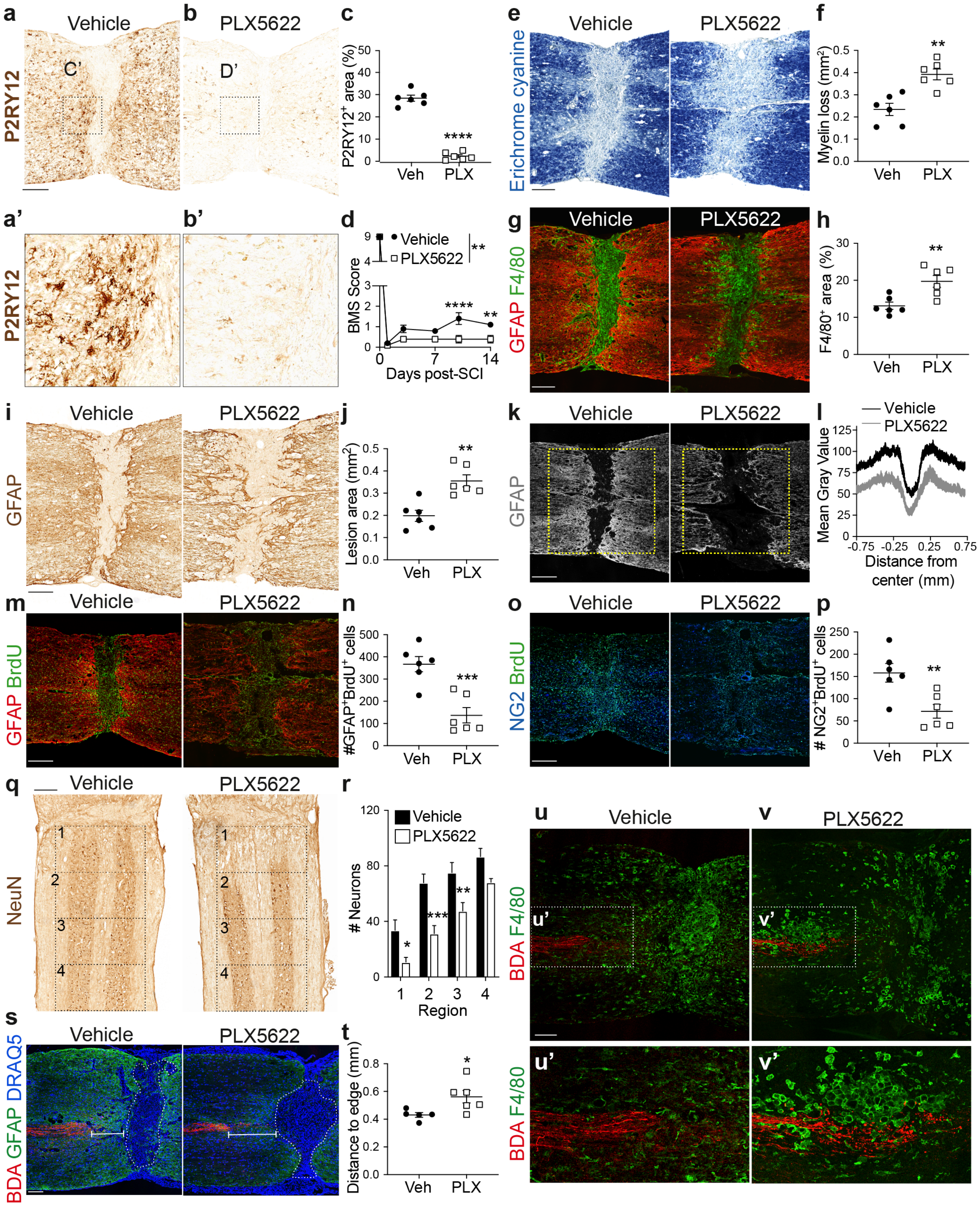
a
b
c
d
e
f
g
h
i
j
k
l
m
n
o
p
q
r
s
t
u
v
a'
b'
u'
v'
Vehicle
PLX5622
P2RY12
C'
D'
P2RY12⁺ area (%)
Veh
PLX
BMS Score
Days post-SCI
Erichrome cyanine
Myelin loss (mm²)
GFAP F4/80
F4/80⁺ area (%)
GFAP
Lesion area (mm²)
Mean Gray Value
Distance from center (mm)
GFAP BrdU
#GFAP⁺BrdU⁺ cells
NG2 BrdU
NG2⁺BrdU⁺ cells
NeuN
Neurons
Region
BDA GFAP DRAQ5
Distance to edge (mm)
BDA F4/80

Supplemental Figure 4: Microglia depletion worsens outcomes from crush SCI. Mice were fed vehicle or PLX5622 from 14 d before injury until 14 d post L1 forceps-crush SCI. **a-c**: Representative horizontal sections (**a, b**) and quantification (**c**) showing PLX5622 depletes spinal cord microglia. Scale bar = 250 μm. **d**: BMS scoring shows worse recovery in mice with microglia depletion. **e, f**: Representative horizontal sections (**e**) and quantification (**f**) showing increased demyelination in spinal cords without microglia. Scale bar = 225 μm. **g, h**: Representative images (**g**) and quantification (**h**) showing spinal cords without microglia have more widespread area of F4/80$^+$ cells beyond the lesion margins. Scale bar = 200 μm. **i-l**: Microglia depletion impairs astroglial border formation and increases lesion area based on GFAP immunoperoxidase staining (**i, j**) and immunofluorescent staining (**k, l**). Scale bar (**i**) = 225 μm, (**k**) = 345 μm. **m-p**: Microglia depletion is associated with reduced astrocyte proliferation (**m. n**) and NG2 cell proliferation (**o, p**). Scale bar (**m, o**) = 320 μm. **q**: Representative images showing staining for NeuN at 14 dpi. Scale bar = 250 μm. **r**: Quantification of NeuN$^+$ cell bodies shows reduced numbers of NeuN$^+$ cells near the lesion in the injured spinal cord without microglia. **s-v**: In a separate experiment, mice received a T9 forceps-crush SCI, injected with an anterograde tracer (biotinylated dextran amine, BDA) into the motor cortex at 14 dpi and perfused at 28 dpi. **s, t**: Representative images (**s**) and quantification (**t**) showing increased BDA$^+$ corticospinal tract axon dieback from the lesion edge when microglia are absent. Scale bar = 167 μm. **u, v**: F8/80$^+$ macrophages were often observed away from the lesion core and clustering around degenerating BDA$^+$ corticospinal axons in spinal cords without microglia. Scale bar = 186 μm. **c, f, h, j, n, p, t**: Student's two-sided t-test; **d**: Two-way repeated measures ANOVA with Bonferroni post hoc tests; **r**: Two-way ANOVA with Bonferroni post hoc tests; n=6 mice per group; **t**: Student's two-sided t-test; n=5-6 mice/group; all data are mean and SEM; *p<0.05; **p<0.01; ***p<0.001. ****p<0.001. **Related to Figure 1.**

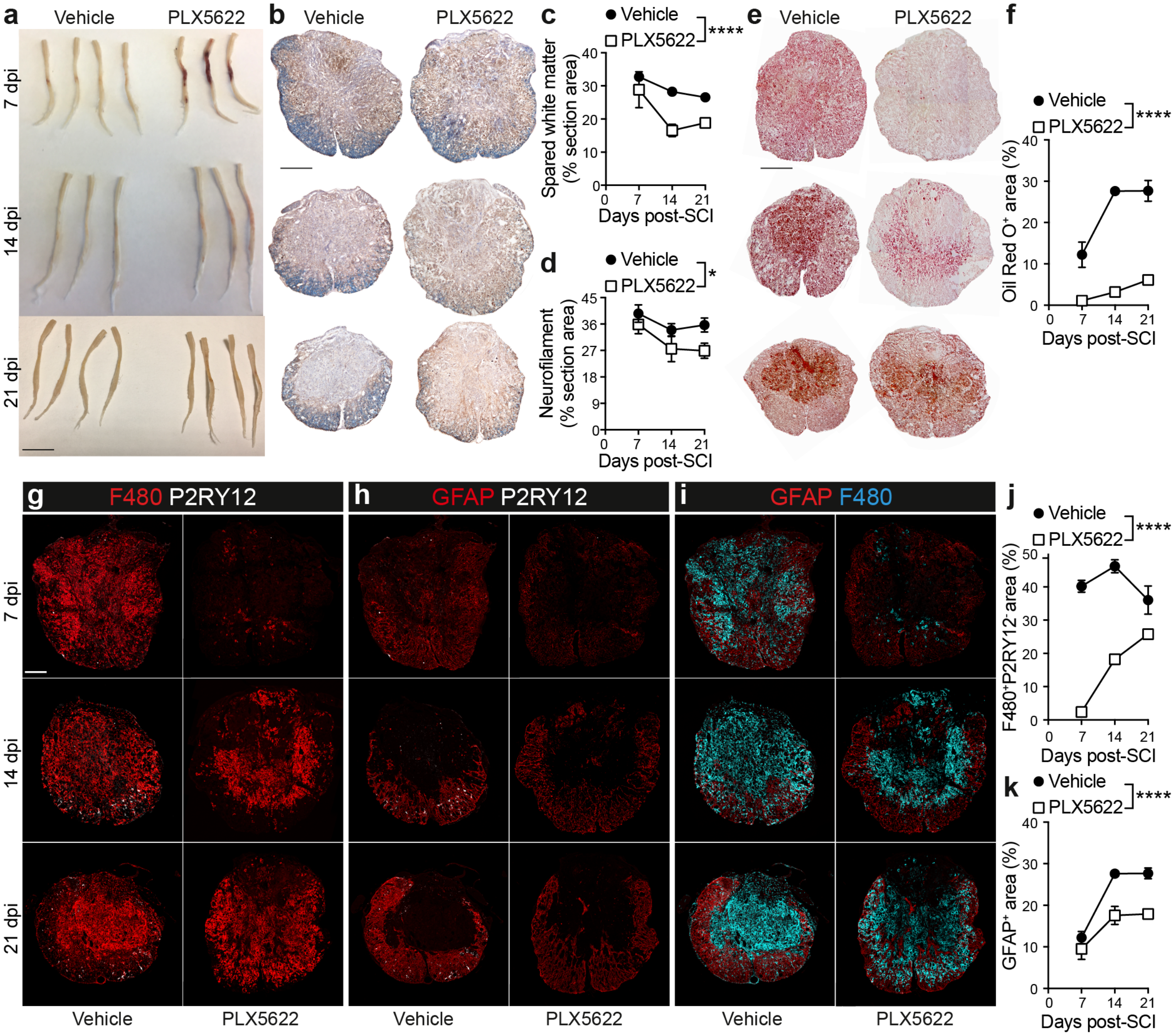

Supplemental Figure 5: Microglia depletion increases tissue pathology in subacute SCI. a: Gross spinal cord images at 7, 14 and 21 dpi. Scale bar = 1 cm. **b-d**: EC (blue) and neurofilament (brown) staining (**b**) and quantification of spared white matter (**c**) and neurofilament (**d**) at the lesion epicenter. **b, e**: Scale bar = 300 µm. **e, f**: Images of Oil Red O staining (**e**) and quantification (**f**) showing impaired lipid clearance in the absence of microglia. **g-k**: Microglia depletion delays recruitment of MDMs ($F4/80^+P2RY12^-$ cells and delays astroglial border formation. Scale bar **g-i** (in **g**, top left) = 200 µm **c, d, f, j, k**: Two-way ANOVA with Bonferroni post-hoc tests, n=3-4 mice per group (n=4 Vehicle 7d, n=3 Vehicle 14 d, n=4 Vehicle 21d, n=3 PLX5622 7d, n=3 PLX5622 14d, n=4 PLX5622 21d); mean ± SEM; *$p<0.05$; ****$p<0.001$. **Related to Figure 1.**

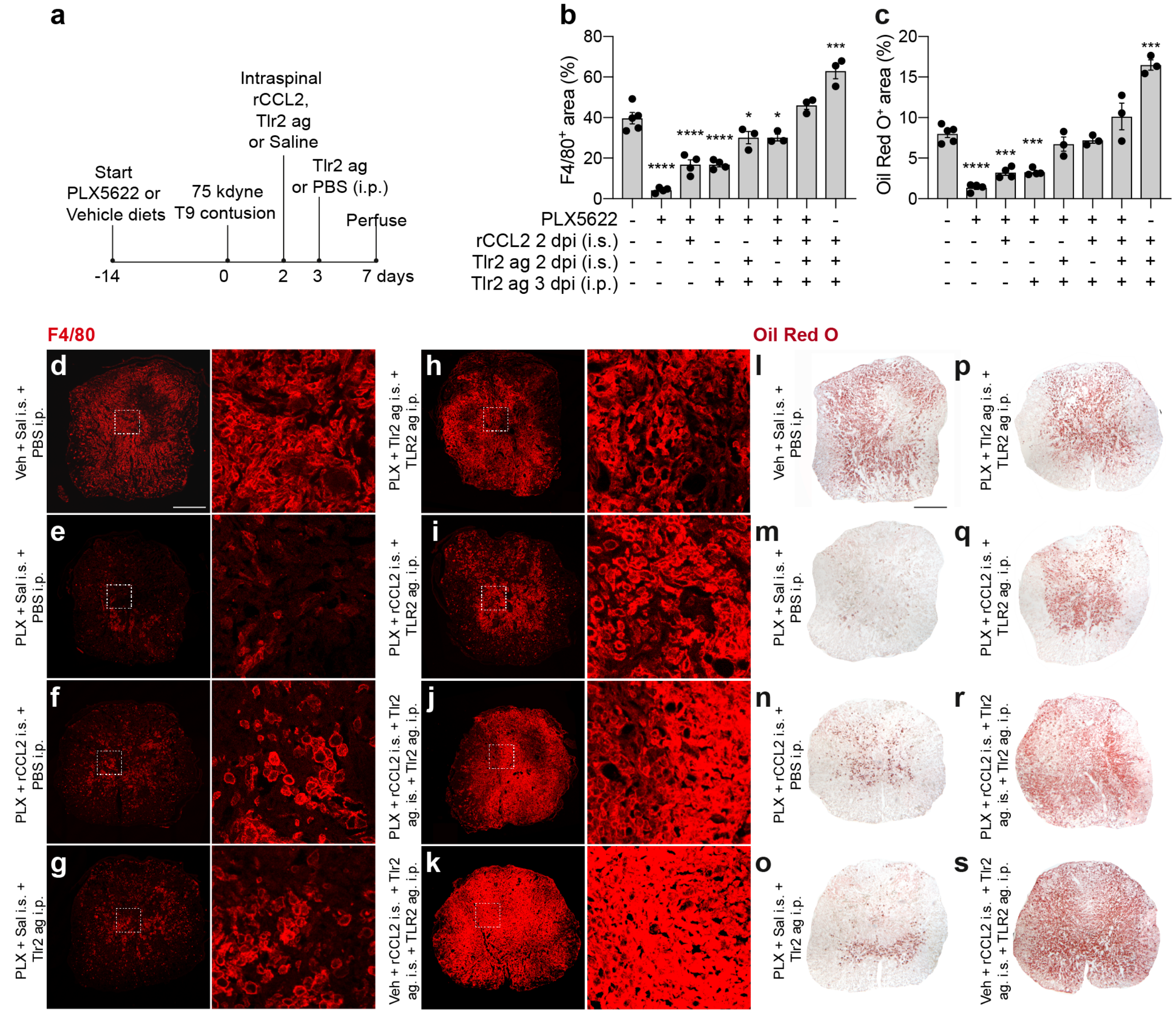

Supplemental Figure 6: Pilot experiments to determine the optimal combination, timing, and route of administration of rCCl2 and Tlr2 agonist (PAM2CSK4, Tlr2 ag) required to elicit a robust macrophage and lipid clearance response in SCI animals without microglia. a: Experimental timeline. Mice received either vehicle or PLX5622 from -14 d to 7 d after 75 kdyne T9 contusion SCI. At 2 dpi, animals were injected intraspinally with 1 µl of rCCL2 + saline, Tlr2 ag + saline, rCCl2 + Tlr2 ag, or saline + saline. At 3 dpi, mice were injected i.p. with Tlr2 ag or PBS. Animals were perfused at 7 dpi. **b, c**: Compared to samples without microglia depletion, microglia depletion with control injections (saline/PBS) showed limited F4/80$^+$ area and Oil Red O$^+$ area in spinal lesions at 7 dpi. However, this phenotype could be partially reversed by administering either CCL2 or Tlr2 ag intraspinally. The most robust increase in an immune response in microglia-depleted spinal cords was achieved by delivering rCCL2 i.s and Tlr2 ag i.s. at 2 dpi and TLR2 ag i.p. at 3 dpi (**b, c,** columns 7 vs. columns 1). This treatment to vehicle mice increased inflammation beyond baseline levels (**b, c**, columns 8 vs. columns 1). **d-s**: Representative images of F4/80$^+$ staining (**d-k**) and Oil Red O staining (**l-s**) in each group. Scale bar (**d-k**) = 225 µm; scale bar (**l-s**) = 200 µm. **b, c**: One-way ANOVA with Dunnett post-hoc tests comparing all groups to Vehicle + saline i.s. + PBS i.p (column 1); n=3-5 mice per group; mean ± SEM; *p<0.05; ***p<0.001; ****p<0.0001. **Related to Figure 3.**

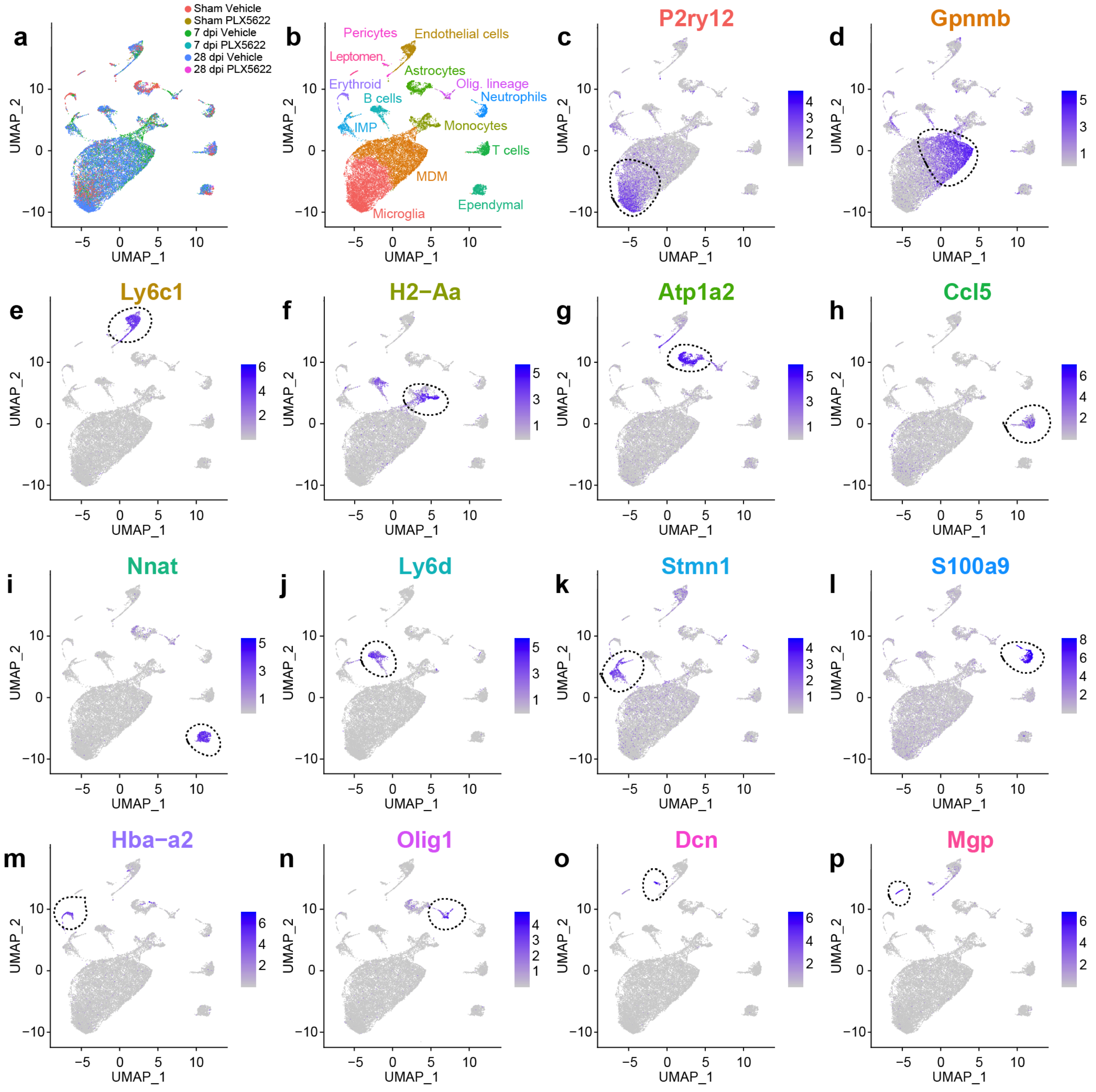

Supplemental Figure 7: UMAP plots showing cell types captured and representative DEGs in each cluster. Cell-type specific genes included *P2ry12* (microglia), *Gpnmb* (monocyte-derived macrophages), *Ly6c1*, (endothelial cells), *H2-Aa*, (undifferentiated monocytes), *Atp1a2* (astrocytes), *Ccl5* (T cells), *Nnat* (ependymal cells), *Ly6d* (B cells), *Stmn1* (intermediate progenitors), *S100a9* (neutrophils), *Hba-a2* (erythroid cells), *Olig1* (oligodendrocyte lineage cells), *Dcn* (pericytes), and *Mgp* (leptomeningeal cells). **Related to Figure 4.**

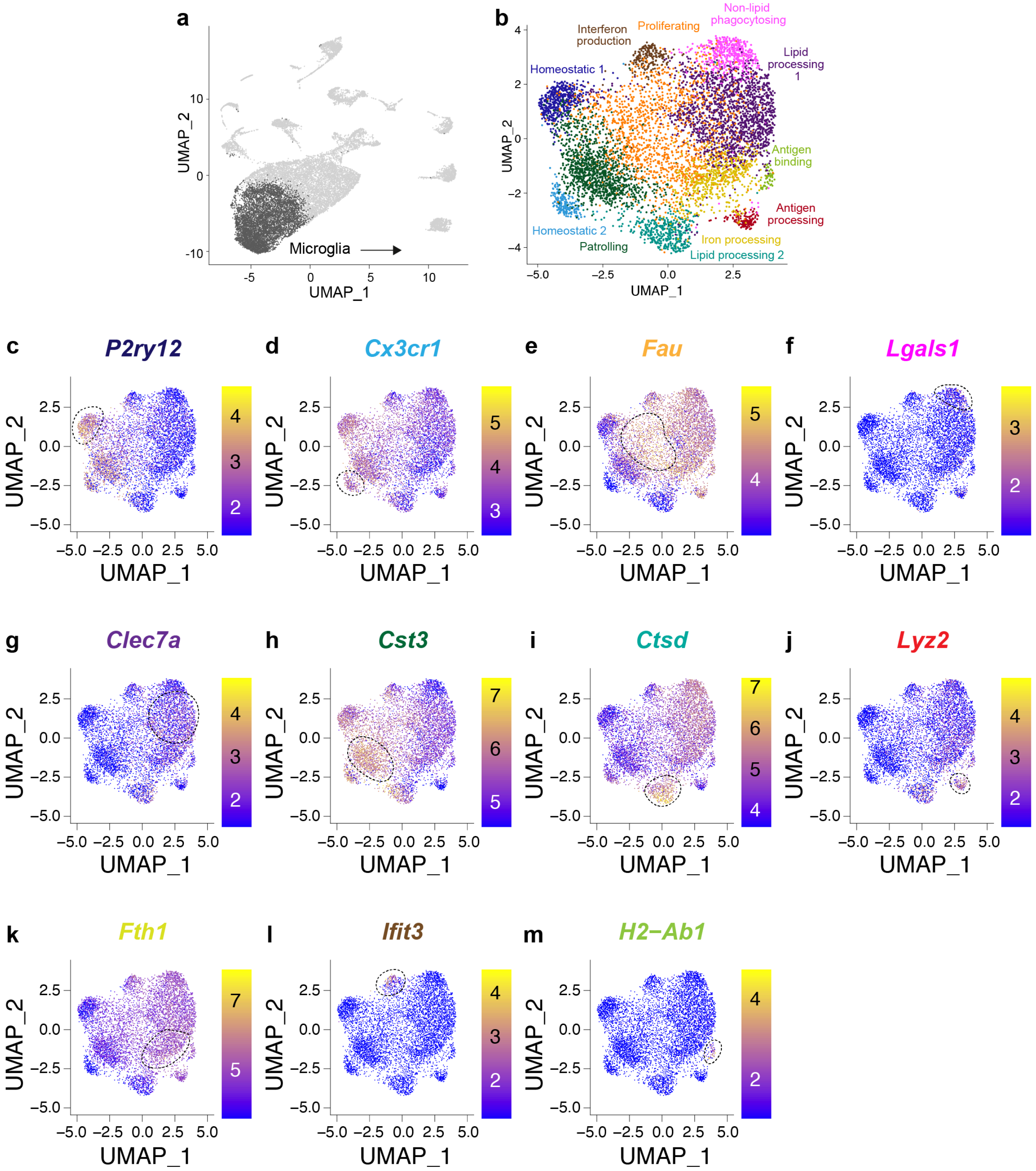

Supplemental Figure 8: UMAP plots showing subsets of microglia and representative DEGs. Related to Figures 4 and 5.

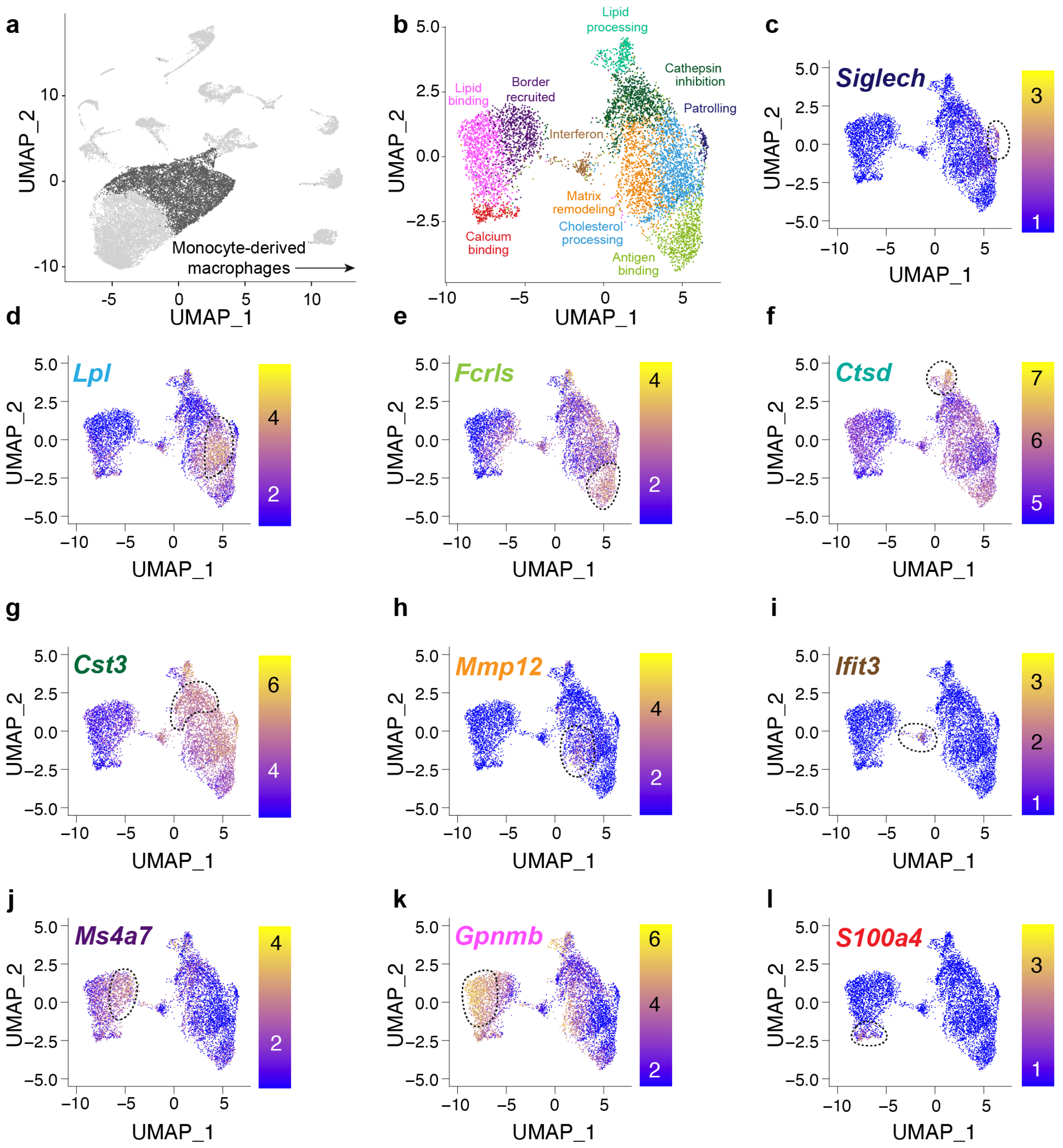

Supplemental Figure 9: UMAP plots showing subsets of MDMs and representative DEGs. Related to Figures 4 and 6.

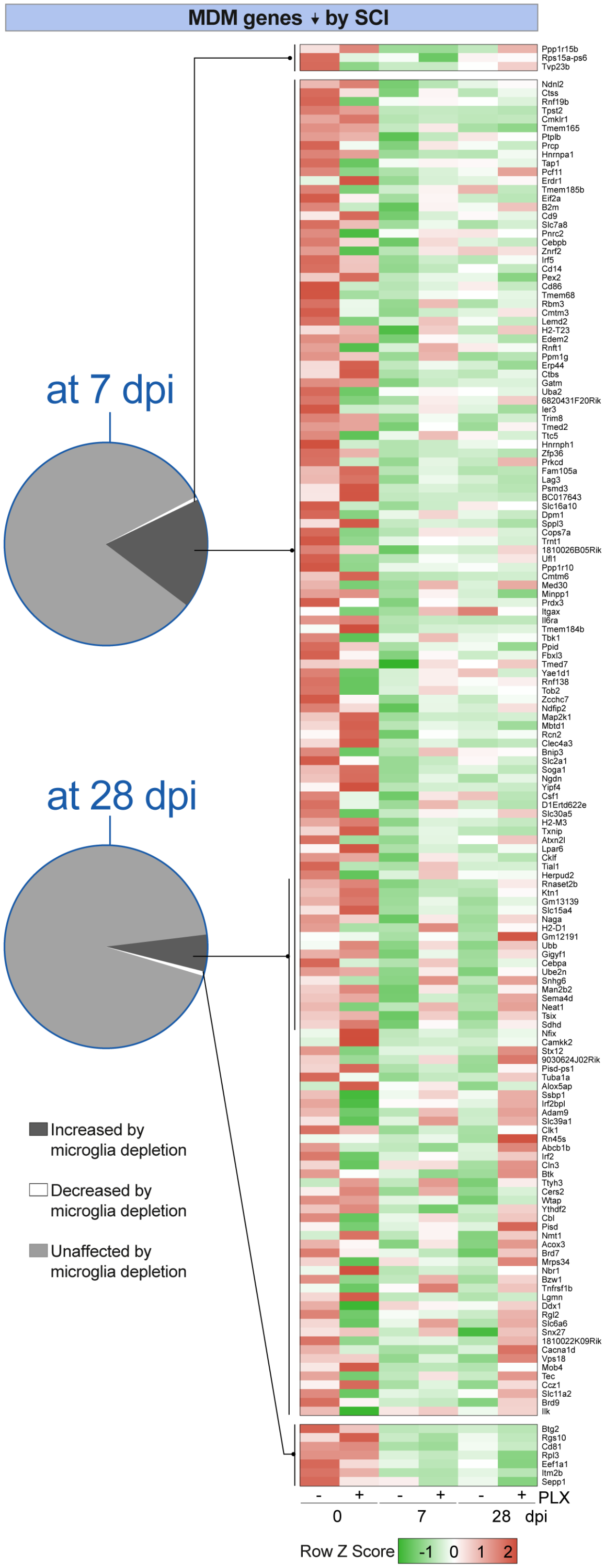

Supplemental Figure 10: The effect of microglia depletion on MDM-specific genes that were also decreased by SCI. Related to Figure 6s.

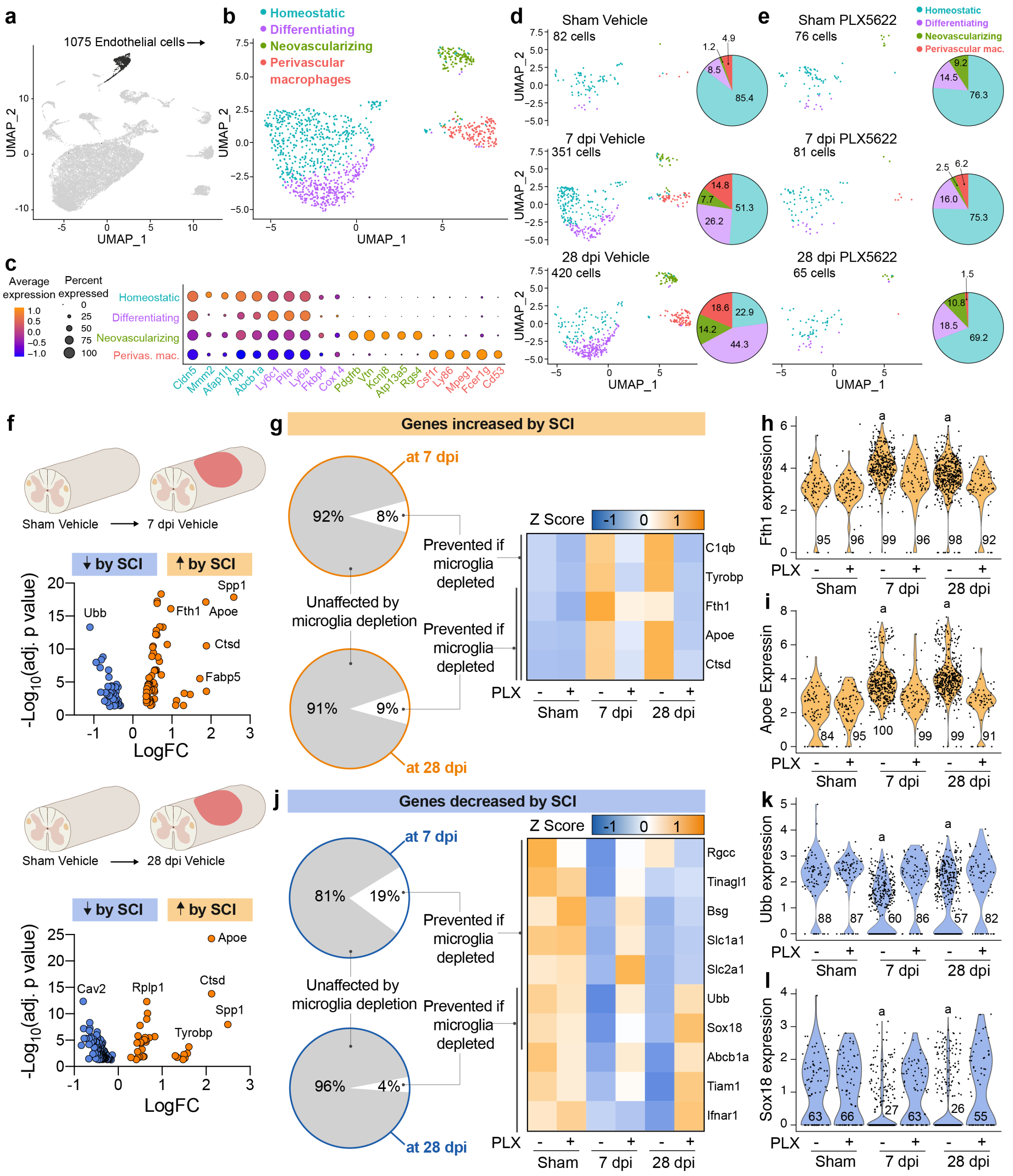

a
1075 Endothelial cells
UMAP_1
UMAP_2
b
Homeostatic
Differentiating
Neovascularizing
Perivascular macrophages
c
Average expression
Percent expressed
Homeostatic
Differentiating
Neovascularizing
Perivas. mac.
Cldn5 Mmrn2 Afap1l1 App Abcb1a Ly6c1 Pltp Ly6a Fkbp4 Cox14 Pdgfrb Vtn Kcnj8 Atp13a5 Rgs4 Csf1r Ly86 Mpeg1 Fcer1g Cd53
d
Sham Vehicle
82 cells
7 dpi Vehicle
351 cells
28 dpi Vehicle
420 cells
e
Sham PLX5622
76 cells
7 dpi PLX5622
81 cells
28 dpi PLX5622
65 cells
Homeostatic
Differentiating
Neovascularizing
Perivascular mac.
f
Sham Vehicle → 7 dpi Vehicle
↓ by SCI
↑ by SCI
-Log10(adj. p value)
LogFC
Sham Vehicle → 28 dpi Vehicle
g
Genes increased by SCI
at 7 dpi
at 28 dpi
Prevented if microglia depleted
Unaffected by microglia depletion
Z Score
C1qb
Tyrobp
Fth1
Apoe
Ctsd
PLX
Sham
7 dpi
28 dpi
h
Fth1 expression
i
Apoe Expressin
j
Genes decreased by SCI
Rgcc
Tinagl1
Bsg
Slc1a1
Slc2a1
Ubb
Sox18
Abcb1a
Tiam1
Ifnar1
k
Ubb expression
l
Sox18 expression

Supplemental Figure 11: Microglia depletion impairs SCI-induced transcriptional changes in endothelia. **a**: The endothelial cell cluster was computationally isolated. **b**: Endothelial cells divided into four subsets based on dominant gene expression, and were characterized as: Homeostatic (cyan), Differentiating (purple), Neovascularizing (green), and Perivascular macrophages (red). **c**: Dot Plot showing the top DEGs in each cluster. **d, e**: UMAP plots showing endothelial cells in each condition. Differentiating, neovascularizing and perivascular macrophages increase as a function of time post-SCI in control endothelial cells, but this shift fails to occur without microglia. Numbers in pie charts show each subset as a percent of total endothelial cells in each group. **f**: Volcano plot showing SCI-induced gene changes in all endothelial cells at 7 dpi (top) and at 28 dpi (bottom). **g**: Most genes increased by SCI in endothelial cells at 7 or 28 dpi were not affected by microglia depletion, although *C1qb, Tyrobp, fth2, Apoe,* and *Ctsd* failed to increase without microglia. **h, i**: Violin plot showing expression of selected genes. The percentages under each violin indicate the percent of cells in the group that express the gene. $^{a}p_{adj}<0.0001$ compared to all other groups, Wilcox rank-sum test, n=65-420 endothelial cells per group pooled from 3-4 mice/group. **j**: Most genes decreased by SCI in endothelial cells at 7 or 28 dpi were not affected by microglia depletion, although genes related to inhibition of the cell cycle and solute transport failed to decrease when microglia were absent. **k, l**: Violin plot showing expression of selected genes. The percentages under each violin indicate the percent of cells in the group that express the gene. $^{a}p_{adj}<0.0001$ compared to all other groups, **h, i, k, l**: Wilcox rank-sum test, n=65-420 endothelial cells per group pooled from 3-4 mice/group. **Related to Figure 4.**

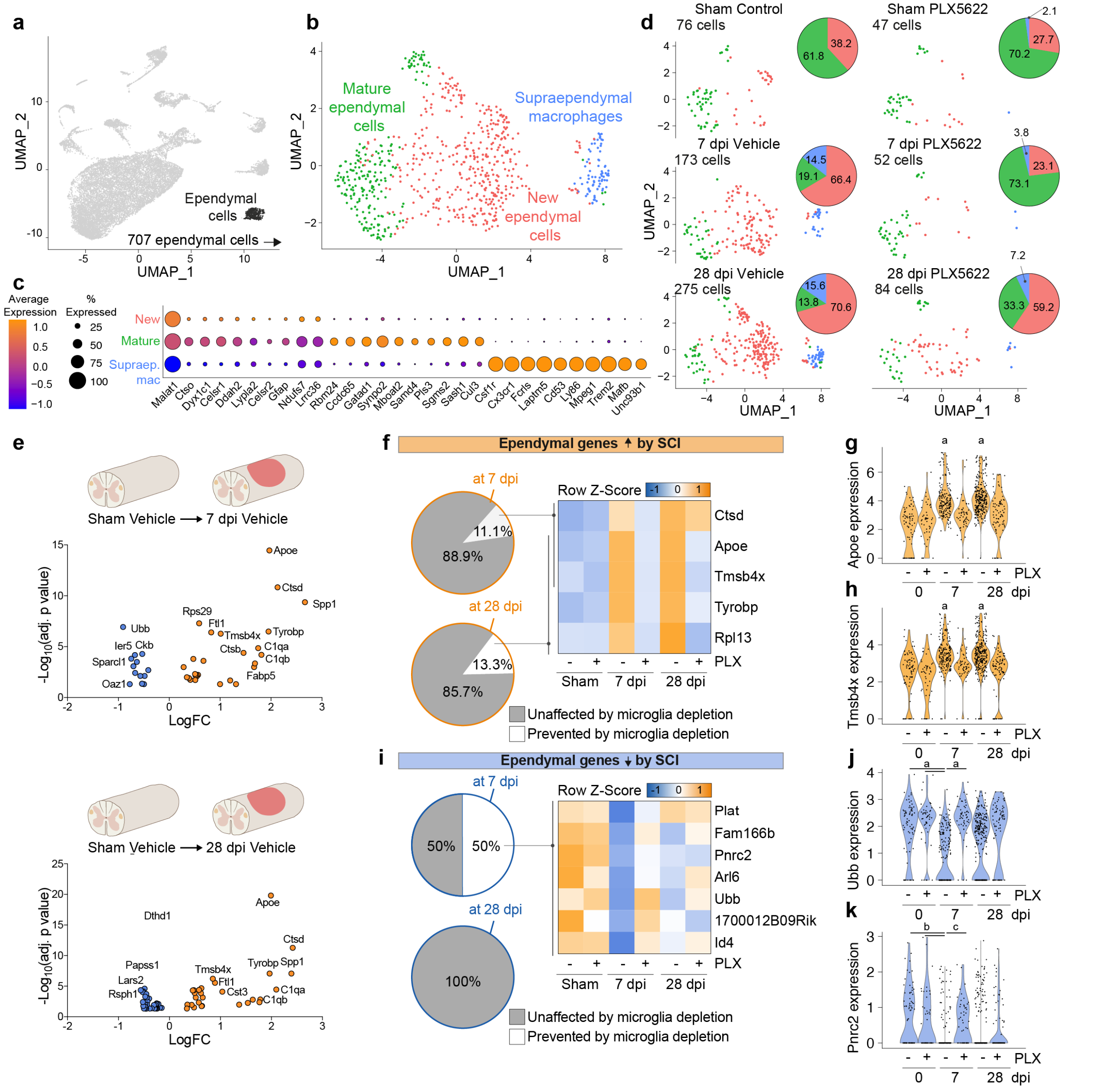

Supplemental Figure 12: SCI increases formation of new ependymal cells. a: The ependymal cell cluster was isolated for analysis between experimental conditions. **b**: Ependymal cells subdivided into three clusters based on dominant gene expression, and were characterized as: mature ependymal cells (green), new ependymal cells (red), and supraependymal macrophages (blue). **c**: Dot Plot showing the top ten DEGs in each cluster. **d**: UMAP plots showing ependymal cells in each condition. In the vehicle group, mature ependymal cells dominate in the intact spinal cord. At 7 and 28 dpi, new ependymal cells dominate, and the proportion of supraependymal macrophages also increases. This shift is delayed and impaired in mice fed PLX5622. Numbers in pie charts are percentages of ependymal cell subsets in each group. **e**: Volcano plot showing SCI-induced gene changes in all ependymal cells at 7 dpi (top) and at 28 dpi (bottom). **f**: Most genes increased by SCI in ependymal cells at 7 or 28 dpi were not affected by microglia depletion, although *Ctsd, Apoe, Tmsb4x, Tyrobp, and Rpl13* failed to increase after SCI if microglia are absent. **g, h**: Violin plot showing selected genes increased by SCI, $^{a}p_{adj}<0.0001$ compared to all other groups, Wilcox rank-sum test, n = 47-275 ependymal cells per group pooled from 3-4 mice per group. **I**: Half of the genes decreased by SCI in endothelial cells at 7 dpi failed to be decreased if microglia were absent. **j, k**: Violin plot showing selected genes decreased by SCI, $^{a}p_{adj}<0.0001$, $^{b}p_{adj}<0.01$, $^{c}p_{adj}<0.05$; **g, h, j, k**: Wilcox rank-sum test, n = 47-275 ependymal cells per group pooled from 3-4 mice per group. **Related to Figure 4.**

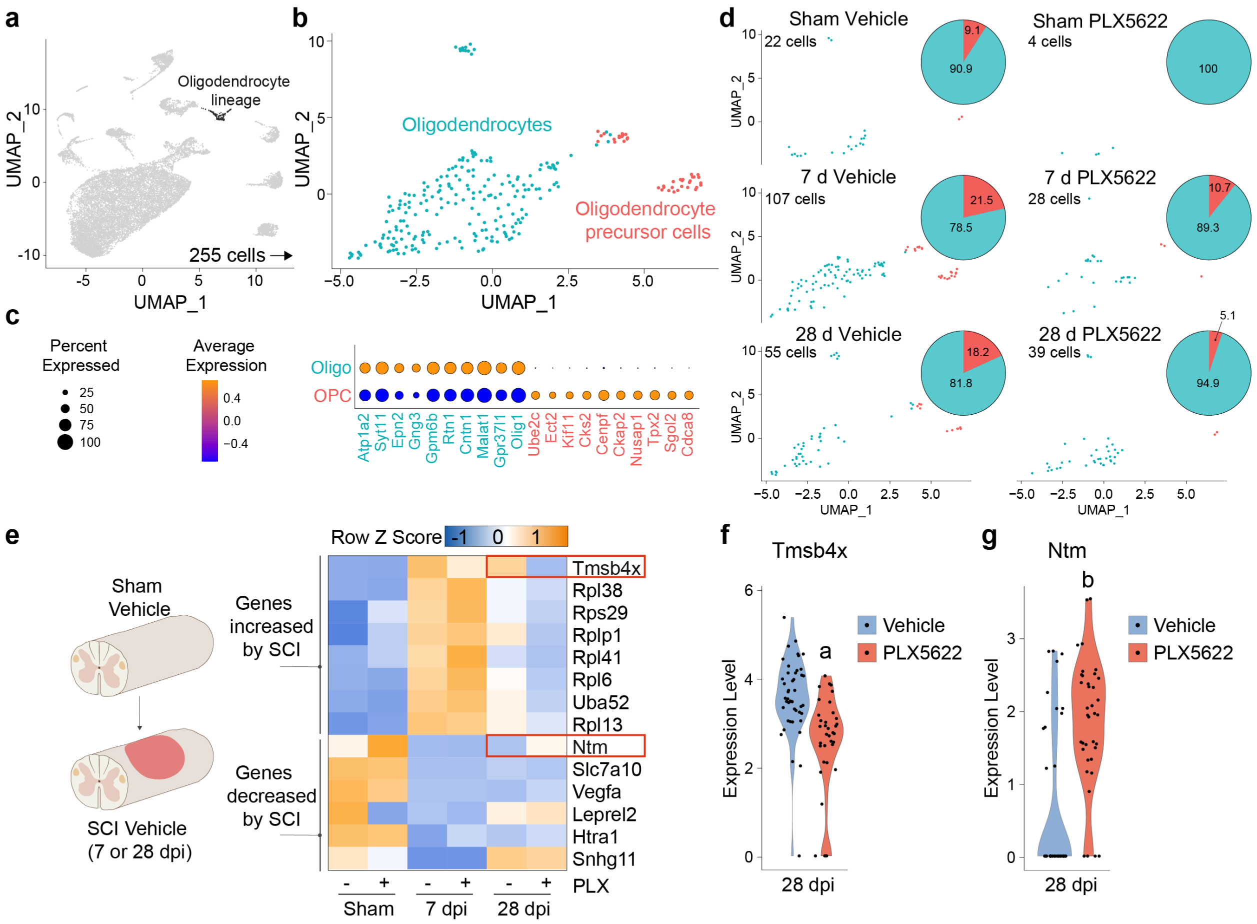

Supplemental Figure 13: SCI increases proliferation genes in oligodendrocyte precursors. a: The oligodendrocyte lineage cluster was isolated for analysis between experimental conditions. **b**: The oligodendrocyte lineage subdivided into oligodendrocyte precursor cells (OPcs, red) and oligodendrocytes (cyan). **c**: Dot Plot showing the top ten DEGs in each cluster. **d**: UMAP plots showing oligodendrocyte lineage cells in each condition. In the vehicle group, the proportion of OPCs expands after SCI. The expansion in OPCs after SCI is limited without microglia. **e**: All the genes in the oligodendrocyte lineage were compared between sham and SCI conditions. Genes that were increased by SCI included *Tmsb4x, Rpl38, Rps29, Rplp1, Rpl41, Rpl6, Uba52* and *Rpl13*. Genes that were decreased by SCI were *Ntm, Slc7a10, Vegfa, Leprel2, Htlra1, and Snhg11*. Of these, at 28 dpi, microglia depletion reversed an SCI-induced increase in thymosin beta 4 (*Tmsb4x)*, which regulates the actin cytoskeleton and promotes cell proliferation, migration and differentiation (**f**). Microglia depletion also reversed the SCI-induced decrease in the cell adhesion molecule neurotrimin (*Ntm*) (**g**). **f, g**: $^{a}p_{adj}<0.001$, $^{b}p_{adj}<0.01$; Wilcox rank-sum test, n=39-55 oligodendrocyte lineage cells per group pooled from 3-4 mice per group. **Related to Figure 4.**

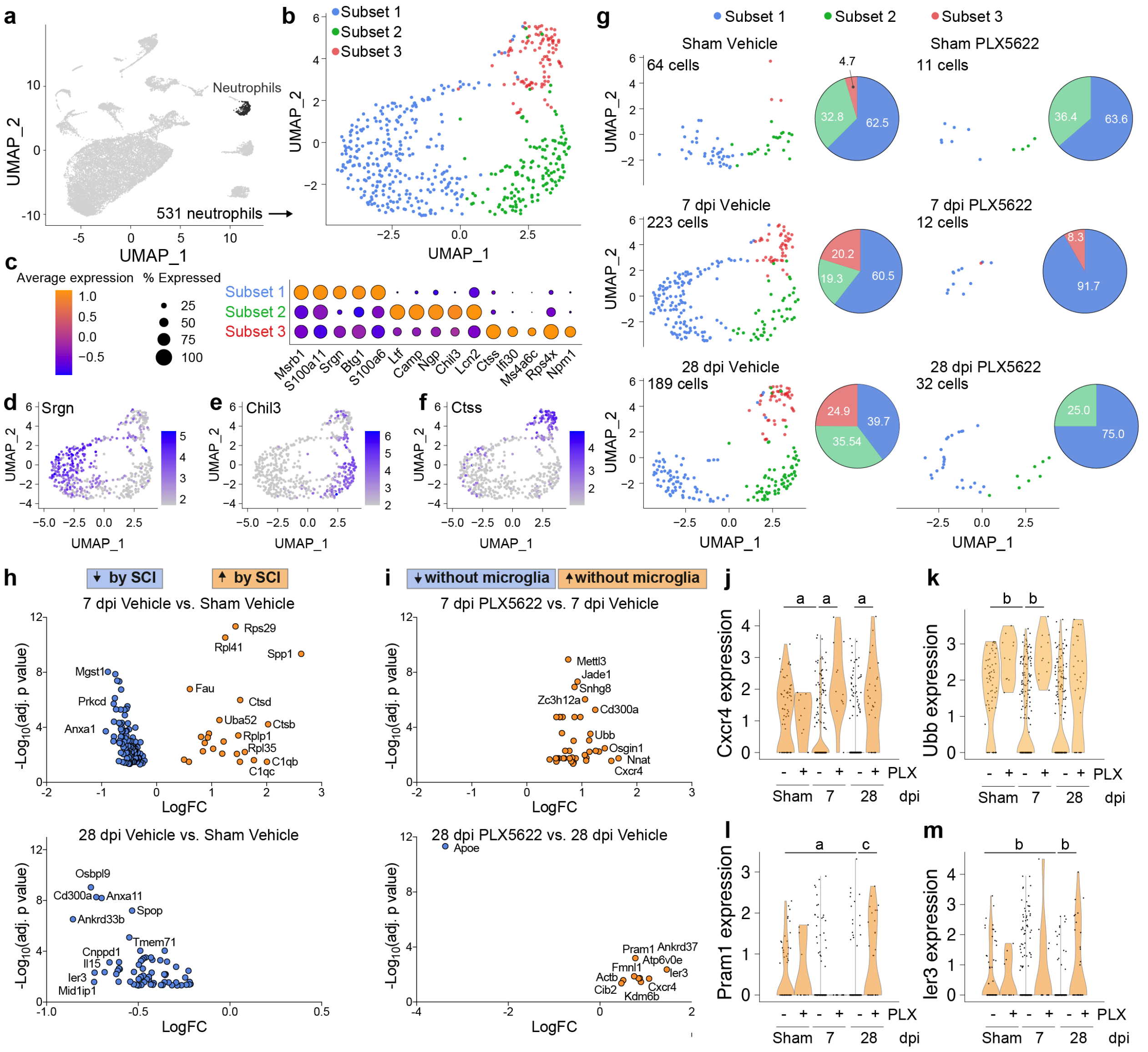

Supplemental Figure 14: SCI and microglia control neutrophil transcriptional phenotype. a: The neutrophil cluster was isolated for analysis between experimental conditions. **b**: Neutrophils subdivided into three clusters based on dominant gene expression, and were characterized as Subset 1, Subset 2 and Subset 3. **c**: Dot Plot showing the top five DEGs in each cluster. **d-f**: UMAP plots showing expression of representative genes (Srgn (**d**), Chil3 (**e**), Ctss (**f**)) in each of the three clusters. **g**: UMAP plots showing neutrophils in each condition. In the vehicle group, the proportion of Subset 3 neutrophils expands after SCI, and this expansion is reversed if microglia are depleted. **h**: Neutrophil genes were compared between sham and SCI conditions. Genes that were upregulated by SCI were mostly lipid processing and inflammation genes (e.g. *Ctsd, C1qb, C1qc*). SCI caused a decrease in cell death genes (e.g. *Anxa11, Anxa1*). **i**: The major effect of microglia depletion on neutrophil transcriptional profile was an increase in neutrophil genes at both 7 dpi (top) and 28 dpi (bottom) (e.g. *Cxcr4, Nnat, Cd300a*). Microglia depletion only decreased one gene in neutrophils at 28 dpi, *Apoe*. **h, i**: Wilcox rank-sum tests. **j-m**: Violin plots showing SCI-affected genes in neutrophils that were decreased by SCI and increased by microglia depletion. **j-m**: $^{a}p_{adj}$ p<0.001, $^{b}p_{adj}$ <0.01, $^{c}p_{adj}$ <0.05; Wilcox rank-sum test, n=11-223 neutrophils per group pooled from 3-4 mice per group. **Related to Figure 4.**

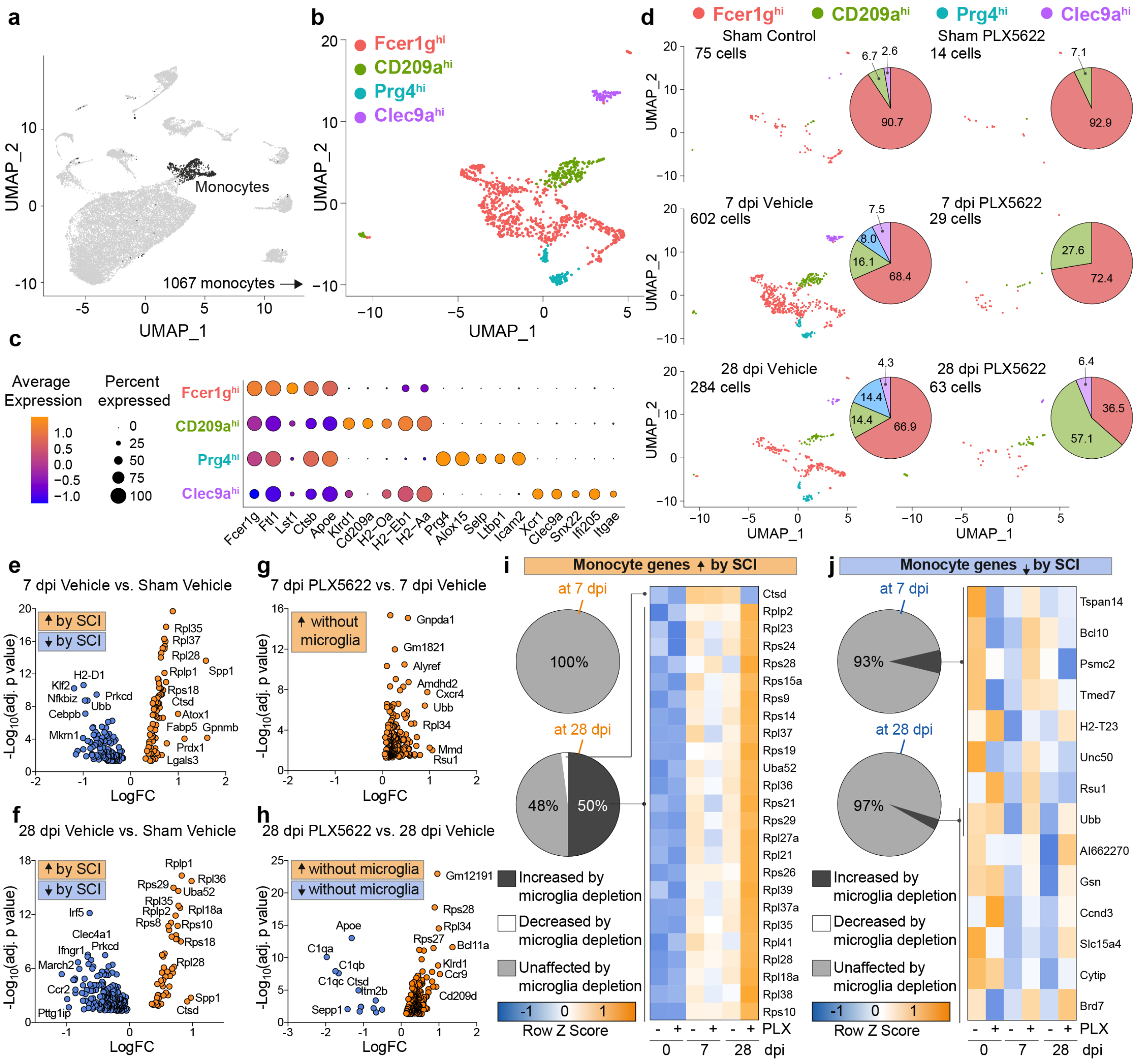

Supplemental Figure 15: SCI and microglia change intraspinal monocyte transcriptional phenotype. a: The monocyte cluster was isolated for analysis between experimental conditions. **b**: Monocytes subdivided into four clusters based on dominant gene expression, and were characterized as Fcer1g^{hi} (red), Cd209a^{hi} (green), Prg4^{hi} (cyan) and Clec9a^{hi} (purple). **c**: Dot Plot showing the top five DEGs in each cluster. **d**: UMAP plots showing monocytes in each condition. **e, f**: Monocyte genes were compared between sham and SCI conditions at 7 dpi (**e**) and 28 dpi (**f**). Genes that were increased by SCI were mostly related to transcription and immune response (e.g. *Rpl, Rps genes, Spp1, Lgals3*). **g, h**: The major effect of microglia depletion on monocytes was to increase in monocyte genes at both 7 dpi (**g**) and 28 dpi (**h**) (e.g. *Ccr9, Cd209d, Rps* genes). **e-h**: Wilcox rank-sum tests. **i, j**: The major effect of microglia depletion on genes that were increased by SCI (**i**) or decreased by SCI (**j**) was to further increase gene expression. n = 14-602 monocytes per group pooled from 3-4 mice per group. **Related to Figure 4.**

Table 1: Genes increased by SCI that fail to increase without microglia presence (n=512), related to Fig. 2F

AB124611	Ccdc88b	Ctsh	Gm14461	Il1rl2	Mmp12	Pon3	Sp140	Unc93b1
Abcc3	Ccl12	Ctss	Gm15413	Il21r	Mmp27	Prdm1	Spink5	Usp18
Abcg3	Ccl2	Ctsz	Gm15880	Il27	Mnda	Prelid2	Spint1	Vav1
Abi3	Ccl3	Cx3cr1	Gm1966	Il2rg	Mndal	Prss46	Srgn	Was
Adam3	Ccl4	Cxcl13	Gm4841	Il7r	Mpeg1	Psd4	St14	Wdfy4
Adam8	Ccl5	Cxcl16	Gm4951	Inpp5d	Ms4a4b	Psmb8	Stab1	Xcr1
Adap2	Ccl6	Cxcl9	Gm5086	Irf5	Ms4a4c	Psmb9	Stap1	Xdh
AF251705	Ccl8	Cyba	Gm5150	Irf7	Ms4a6b	Ptafr	Stat4	Xlr
AI427809	Ccl9	Cybb	Gm5431	Irf8	Ms4a6c	Ptk2b	Stat6	Xlr4a
AI607873	Ccr1	Cysltr1	Gm5547	Irg1	Ms4a6d	Ptpn18	Stxbp2	Zbp1
AI662270	Cd14	Cyth4	Gm6377	Itgam	Ms4a7	Ptpn6	Susd3	Zc3h12d
Aif1	Cd180	Cytip	Gna15	Itgb2	Msr1	Ptpn7	Syk	Zranb3
Alox5ap	Cd200r1	D830046C22Rik	Gngt2	Itgb5	Mx1	Ptprc	Tagap	1300002K09Rik
Ang	Cd200r4	Dab2	Golm1	Kcnk6	Mylip	Pycard	Tbxas1	1600010M07Rik
Anpep	Cd209a	Ddx60	Gp49a	Kcnn4	Myo1f	Pydc4	Tcirg1	1810011H11Rik
Aoah	Cd22	Dennd1c	Gpnmb	Klhl38	Myo1g	Pyhin1	Tfec	3830403N18Rik
Apbb1ip	Cd244	Dennd2c	Gpr157	Klhl6	Naglu	Rab19	Tgfb1	4632428N05Rik
Apobec1	Cd28	Dhrs3	Gpr160	Klk7	Naip2	Rab20	Tgfbr2	4930486L24Rik
Apobr	Cd300a	Dhrs9	Gpr183	Klra17	Naip5	Rab32	Ticam2	4930556M19Rik
Apoc1	Cd300lb	Dhx58	Gpr35	Klra3	Naip6	Rac2	Tifab	5031425F14Rik
Apoc2	Cd300ld	Dnase1l1	Gpr65	Klrb1b	Naip7	Raet1d	Tjp3	5430427O19Rik
Apoc4	Cd300lf	Dnase2a	Gpr84	Klrd1	Ncf1	Rasal3	Tlr1	5430435G22Rik
Apoe	Cd33	Dock2	Gpsm3	Kmo	Ncf2	Rbm47	Tlr13	5830416I19Rik
Arg1	Cd36	Dock8	Grn	Lair1	Ncf4	Rcvrn	Tlr2	5830432E09Rik
Arhgap19	Cd37	Dpep2	Gsdmd	Laptm5	Nckap1l	Rgs1	Tlr4	6330407A03Rik
Arhgap25	Cd4	Elf4	Gsg1	Lat2	Neurl3	Rgs18	Tlr6	9930111J21Rik1
Arhgap30	Cd48	Emr1	Guca1a	Lcp1	Nfam1	Rhbdf2	Tlr7	9930111J21Rik2
Arhgap9	Cd52	Epsti1	Gusb	Lcp2	Nlrc4	Rhoh	Tlr8	A130077B15Rik
Arhgdib	Cd53	F630028O10Rik	H2-DMb1	Lgals3bp	Nlrp1a	Rin3	Tlr9	A430093F15Rik
Arl11	Cd5l	F630111L10Rik	H2-K1	Lgals9	Nlrp1b	Rnase4	Tmem106a	A530088E08Rik
Atp8b4	Cd68	F9	Hal	Lilrb4	Nlrp3	Runx1	Tmem150b	A630001G21Rik
AU022793	Cd72	Fam105a	Havcr2	Lipa	Npl	Runx3	Tmem154	A630033H20Rik
Avil	Cd84	Fam111a	Hck	LOC100038947	Nuak2	Saa3	Tmem173	
B2m	Cd86	Fam167b	Hcls1	LOC100503676	Oas1a	Saa4	Tmem221	
B430306N03Rik	Cdca7l	Fam26f	Hcst	Lpar5	Oas1g	Samsn1	Tmem37	
B4galnt2	Cdk6	Fam46c	Hexa	Lpl	Oasl1	Sash3	Tmem71	
B4galt1	Ceacam16	Fcer1g	Hexb	Lpxn	Oasl2	Selenbp2	Tmem86a	
Batf	Cebpa	Fcgr1	Hhex	Lrmp	Olfml3	Serpinb1c	Tnf	
Baz1a	Cfp	Fcgr2b	Hist1h2ab	Lrrc25	Olfr111	Sh2d1b1	Tnfaip3	
BC021767	Ch25h	Fcgr3	Hist1h2ag	Lrrc39	Osm	Sh3bp2	Tnfaip8	
Bcl2a1a	Ciita	Fcgr4	Hk2	Lst1	P2ry6	Siglec1	Tnfaip8l2	
Bcl2a1b	Clec12a	Fcrl1	Hk3	Ly86	Parp14	Sirpb1a	Tnfrsf11a	
Bcl2a1d	Clec2i	Fcrls	Hlx	Ly9	Parp9	Sirpb1b	Tnfrsf13b	
Bin2	Clec5a	Fermt3	Hmga2-ps1	Lyl1	Parvg	Sla	Tnfrsf1b	
Blnk	Clec7a	Fes	Hmha1	Lyn	Pdcd1	Slamf6	Tnip3	
Bst2	Cmtm7	Fgr	Hpgds	Lyz1	Pf4	Slamf8	Tns4	
Btk	Cnr2	Fli1	Hpse	Lyz2	Phldb3	Slamf9	Traf3ip3	
C130050O18Rik	Col6a5	Folr2	Htr2b	Lyzl4	Pik3ap1	Slc11a1	Trem2	
C1qa	Cotl1	Frrs1	Hvcn1	Mafb	Pik3cg	Slc15a3	Treml2	
C1qb	Cox6a2	Fyb	Ifi204	Man2b1	Pik3r5	Slc16a10	Trim14	
C1qc	Csf1r	Galnt3	Ifi30	Map4k1	Pilra	Slc16a3	Trim30a	
C3ar1	Csf2rb	Gbp10	Ifi44	Mcm3	Pira2	Slc17a9	Trpv1	
C5ar1	Csf2rb2	Gbp8	Igf1	Mcoln2	Pira6	Slc37a2	Tspan32	
C6	Csf3r	Gcnt1	Igsf6	Mcoln3	Pla2g15	Slc39a4	Tyrobp	
C920009B18Rik	Cst7	Gdf3	Iigp1	Mcpt2	Plau	Slc7a7	Uba7	
Capg	Ctla2b	Glipr1	Ikzf1	Mdfic	Plcb2	Slfn10-ps	Ucp2	
Car9	Ctsa	Glrp1	Il10ra	Mir142	Plcg2	Slfn2	Ugt1a6b	
Card11	Ctsb	Gm12185	Il10rb	Mis18bp1	Pld4	Slfn8	Ugt1a7c	
Casp1	Ctsc	Gm12250	Il12rb2	Mki67	Plek	Snx20	Ulbp1	
Casp8	Ctsd	Gm13212	Il1a	Mmp10	Plin2	Sp110	Unc13d	

Table 2: Reactome pathways and sub-pathways increased by SCI that depend on microglia

Immune System			Signal transduction	Metabolism	Cell cycle	Hemostasis
Innate Immunity<br><br>•Toll-like receptor cascades<br>•Complement cascade<br>•NLR signaling<br>•Cytosolic sensors of pathogen-associated DNA<br>•Fc gamma receptor dependent phagocytosis<br>•DAP12 interactions<br>•Fc epsilon receptor signaling<br>•C-type lectin receptors<br>•Antimicrobial peptides<br>•Neutrophil degranulation<br>•ROS/RNS production in phagocytes	*Cytokine signaling by:*<br><br>•Interleukins<br>•Interferons<br>•Growth hormone receptor<br>•TNFR2 non-canonical NF-kB pathway<br>•FLT3	*Adaptive Immunity*<br><br>•TCR signaling<br>•Co-stimulation of CD28 family<br>•Signaling by B cell receptor<br>•MHC class I antigen processing & presentation<br>•MHC class II antigen presentation<br>•Lymphoid-non-lymphoid cell interactions<br>•Butyrophilin family interactions	•Receptor tyrosine kinases<br>•TGF-beta family<br>•GPCR<br>•WNT<br>•Hedgehog<br>•Integrins<br>•Nuclear receptors<br>•MAPK family<br>•Intracellular signaling by second messengers<br>•Rho GTPases<br>•Non-receptor tyrosine kinases<br>•mTOR<br>•Death receptors<br>•Erythropoietin	•Carbohydrate metabolism<br>•Lipid metabolism<br>•Inositol phosphate metabolism<br>•Integration of energy metabolism<br>•TCA cycle and electron transport<br>•Nucleotides<br>•Vitamins and cofactor metabolism<br>•Amino acids and derivatives<br>•Biological oxidations<br>•Reversible hydration of CO_2	•G1/S checkpoints<br>•G2/M checkpoints<br>•Mitotic G1 phase and G1/S transition<br>•S Phase<br>•Mitotic G2-G2/M phases<br>•M phase<br>•Regulation of mitosis<br>•Nucleosome assembly<br>•Telomere maintenance	•Platelet homeostasis<br>•Platelet adhesion to exposed collagens<br>•Platelet activation, signaling and aggregation<br>•Clotting cascade<br>•Dissolution of fibrin clot<br>•Cell surface interactions at vascular wall<br>•Megakaryocyte development and platelet production

Table 3: Specific Genes in the Biological Process GO Terms: Phagocytosis, Response to cytokine, Cytokine production, Endocytosis, and Protein secretion (see Fig. 2K).	
**Biological Process(es)**	(**# Genes**) Gene names
Five biological processes	
Phagocytosis<br>Response to cytokine<br>Cytokine production<br>Endocytosis<br>Protein secretion	(**4**) Syk, Pycard, Tnf, Tlr2
Four biological processes	
Phagocytosis<br>Response to cytokine<br>Cytokine production<br>Endocytosis	(**6**) Fcer1g, Slc11a1, Ccl2, Cyba, Irf8, Ptprc
Phagocytosis<br>Response to cytokine<br>Cytokine production<br>Protein secretion	(**3**) Tgfb1, Fgr, Cd36
Response to cytokine<br>Cytokine production<br>Endocytosis<br>Protein secretion	(**1**) Cd14
Three biological processes	
Phagocytosis<br>Cytokine production<br>Endocytosis	(**2**) Nckap1l, Fcgr2b
Phagocytosis<br>Response to cytokine<br>Endocytosis	(**5**) Aif1, Cd300lf, Rab20, Trem2, Stap1
Cytokine production<br>Response to cytokine<br>Protein secretion	(**9**) Osm, Mmp12, Casp1, Il1a, Tnfrsf1b, Ccl5, Ccl3, Csf1r, Tlr4
Cytokine production<br>Endocytosis<br>Protein secretion	(**1**) Htr2b
Two biological processes	
Phagocytosis<br>Endocytosis	(**14**) P2ry6, Myo1g, Arhgap25, Fcgr1, Ncf2, Msr1, Ncf4, Unc13d, Cd300a, Vav1, Dock2, Itgb2, Pld4, Hck
Cytokine production<br>Response to cytokine	(**13**) Ptpn6, Tmem173, Irf7, Btk, Cd4, Pf4, Il12rb2, Ccl4, Ticam2, Cx3cr1, Il1rl2, Arg1, Bst2
Cytokine production<br>Endocytosis	(**2**) B2m, Plcg2
Cytokine production<br>Protein secretion	(**22**) Nlrp3, Lyn, Nlrp1b, Tlr6, Gsdmd, Tlr1, Lgals9, Lcp2, Tlr9, Havcr2, Card11, Lpl, Srgn, Tnfaip3, Cd200r1, Unc93b1, Nlrc4, Clec5a, Nlrp1a, Mcoln2, Tlr8, Cd84
Response to cytokine<br>Endocytosis	(**2**) Was, Cxcl16
Endocytosis<br>Protein secretion	(**1**) Cd22
One biological process	
Cytokine production	(**26**) Sash3, Clec2i, Ptafr, Cybb, Tlr7, Trim30a, Il27, Cd28, Hpse, Lipa, Naip5, Dhx58, Gpnmb, Adam8, Ly9, Tnfaip8, Cd300ld, C5ar1, Slamf6, Ulbp1, Inpp5d, Batf, Ccdc88b, Nfam1, C3ar1, Gpsm3
Response to cytokine	(**42**) Parp9, Ccl6, Ccr1, Ccl8, Mndal, Gbp10, Hcls1, Tnfrsf11a, Saa3, Hk2, Ccl9, Casp8, 9930111J21Rik1, Csf3r, Iigp1, Laptm5, Ifi204, Csf2rb2, Gm12185, Ciita, Il10ra, Il2rg, Csf2rb, Dock8, Cebpa, Ccl12, Gm4841, Trpv1, Stxbp2, Ptk2b, Il7r, Gpr35, Stat6, Il21r, Gm4951, Cxcl13, Zbp1, Parp14, Kmo, Gbp8, Stat4, Cxcl9
Endocytosis	(**12**) Dab2, Dennd1c, Apobr, Fcrls, Tgfbr2, Cd209a, Lgals3bp, Pik3cg, Cd5l, Apoe, Apoc1, Siglec1
Protein secretion	(**6**) Kcnn4, Ang, Plek, Rhbdf2, Ucp2, Igf1

**Key Resources Table: Immunostaining reagents**			
Antigen	***Host, dilution***	***RRID***	***Vendor, catalog number***
Primary antibodies			
**BrdU**	Sheep, 1:200	AB_302944	Abcam, ab2284
**CD11b**	Rat, 1:200	AB_321293	Bio-Rad/AbD Serotec, MCA74G
**CD68**	Rat, 1:500	AB_322219	Bio-rad/AbD Serotec, MCA1957
**F4/80**	Rat, 1:500	AB_323279	Bio-Rad/AbD Serotec, MCA497R
**GFAP**	Rabbit, 1:500	AB_10013382	Dako, Z0334
**GSTπ**	Rabbit, 1:500	AB_10778283	Biorbyt, orb18037
**Iba-1**	Rabbit, 1:500	AB_839504	Wako, 019-19741
**MBP**	Rabbit, 1:500	AB_2313550	Aves Labs, MBP
**NeuN**	Rabbit, 1:400	AB_10711153	Abcam, ab104225
**NFH**	Chicken, 1:500	AB_2313552	Aves Labs, NF-H
**NG2**	Rabbit, 1:500	AB_91789	Millipore, AB5320
**P2RY12**	Rabbit, 1:1000	AB_2298886	Anaspec, AS-55043A
Secondary amplification			
**Biotin-chicken IgY**	Goat, 1:1000	AB_3213506	Aves Labs, B-1005
**Biotin-rabbit IgG**	Goat, 1:1000	AB_954902	Abcam, Ab6720
**Chicken IgY 488**	Goat, 1:500	AB_2534096	Thermo Fisher Scientific, A-11039
**Chicken IgY 546**	Goat, 1:500	AB_2534097	Thermo Fisher Scientific, A-11040
**Rabbit IgG-488**	Goat, 1:500	AB_2576217	Thermo Fisher Scientific, A-11034
**Rabbit IgG-546**	Goat, 1:500	AB_2534093	Thermo Fisher Scientific, A-11035
**Rabbit IgG-633**	Goat, 1:500	AB_2535731	Thermo Fisher Scientific, A-21070
**Rat IgG 546**	Goat, 1:500	AB_2534125	Thermo Fisher Scientific, A-11081
**Streptavidin 488**	1:250	AB_2336881	Thermo Fisher Scientific, S11223
**Streptavidin 546**	1:500	AB_2532130	Thermo Fisher Scientific, S11225A
**Other dyes**			
**DRAQ5**	1:4000		Abcam, ab108410
**Oil Red O**	In 70% EtoH		Sigma, O0625
**Zombie Green**	1:100		BioLegend 423111
**Flow cytometry**			
**CD11b-PeCy7**	Rat, 1:100	AB_394491	BD Bioscience, 552850
**CD11c-BV711**	Hamster, 1:100	AB_2734778	BD Bioscience, 563048
**CD16/32**	Rat, 1:200	AB_394657	BD Biosciences 553142
**Ly6C-APC**	Rat, 1:100	AB_1727554	BD Bioscience, 560595
**Ly6G-PE**	Rat, 1:100	AB_394208	BD Bioscience, 551461
