# Supplementary material for: Microglia coordinate cellular interactions during spinal cord repair in mice
Source: Nat Commun. 2022 Jul 14;13:4096. doi: 10.1038/s41467-022-31797-0 (PMC9283484; doi:10.1038/s41467-022-31797-0)
Supplement: Supplementary file 3 — Reporting Summary [file 41467_2022_31797_MOESM3_ESM.pdf]

nature portfolio

Corresponding author(s): Phillip G. Popovich

Last updated by author(s): June 3 2022

Reporting Summary

Nature Portfolio wishes to improve the reproducibility of the work that we publish. This form provides structure for consistency and transparency in reporting. For further information on Nature Portfolio policies, see our Editorial Policies and the Editorial Policy Checklist.

Statistics

For all statistical analyses, confirm that the following items are present in the figure legend, table legend, main text, or Methods section.

n/a	Confirmed	
☐	☒	The exact sample size (*n*) for each experimental group/condition, given as a discrete number and unit of measurement
☐	☒	A statement on whether measurements were taken from distinct samples or whether the same sample was measured repeatedly
☐	☒	The statistical test(s) used AND whether they are one- or two-sided<br>*Only common tests should be described solely by name; describe more complex techniques in the Methods section.*
☐	☒	A description of all covariates tested
☐	☒	A description of any assumptions or corrections, such as tests of normality and adjustment for multiple comparisons
☐	☒	A full description of the statistical parameters including central tendency (e.g. means) or other basic estimates (e.g. regression coefficient) AND variation (e.g. standard deviation) or associated estimates of uncertainty (e.g. confidence intervals)
☐	☒	For null hypothesis testing, the test statistic (e.g. F, t, r) with confidence intervals, effect sizes, degrees of freedom and P value noted<br>*Give P values as exact values whenever suitable.*
☒	☐	For Bayesian analysis, information on the choice of priors and Markov chain Monte Carlo settings
☒	☐	For hierarchical and complex designs, identification of the appropriate level for tests and full reporting of outcomes
☒	☐	Estimates of effect sizes (e.g. Cohen's d, Pearson's r), indicating how they were calculated

Our web collection on statistics for biologists contains articles on many of the points above.

Software and code

Policy information about availability of computer code

Data collection

Bulk tissue RNA-seq was carried out by the UCLA Neuroscience Genomics Core (UNGC). Reads were aligned to the mouse GRCm38 reference genome using STAR (v.2.4.0). Read counts for RefSeq genes (mm10) were generated by HTSeq v.0.6.1. Low-count genes were filtered and fragments per kilobase per million mapped reads (FPKM) values were generated. In total, 17,939 genes were identified. Single cell RNA sequencing was conducted on HiSeq4000 (Novogene). Fastq sequence files were de-multiplexed, aligned, and annotated using the mouse ENSMBL database and Cell Ranger software. Gene expression was counted using unique molecular identifier barcodes, and gene-cell matrices were constructed.

Data analysis

For bulk RNAseq, differentially expressed genes were identified using the DESeq2 (v l.14.1) and verified using Gene Network Analyst 3.0 (Zhou et al., 2019). Pathway and network analysis was performed using: Web-based Gene Set Analysis Toolkit (WebGestalt), with the Gene Ontology Reference Genome set as the reference gene list (Liao, et al., 2019), Reactome pathway database (Jassal, et al., 2020), Genemania, and the The Bioinformatics and Evolutionary Genomics server. Fastq sequence files were de-multiplexed, aligned, and annotated using the mouse ENSMBL database and Cell Ranger v3.0 software. Data processing and visualizations were performed using Seurat package (v.3.2.0) in R (3.6.3) (Stuart et al., 2019) and Monocle 3 (v1.0.0) (Trapnell et al., 2014). The cell-cell communications were predicted using the R package CellChat (v1.0.0) (Jin, et al., 2021). FACS Diva software (v9.0) (BD) and FlowJo (v10.8.1) (BD) were used for analysis of flow cytometry data. Graph Pad Prism (v 8.0.2) was used for general data visualization and statistical analyses. Figures were prepared in Adobe Illustrator (v 25.2.3) and graphical images created with Biorender.com. Source data are provided with this paper and deposited to the Open Data Commons for Spinal Cord Injury (ODC-SCI) Accession: 695, DOI:10.34945/F5B012, [https://odc-sci.org/data/695]. Gene expression data for bulk and single cell RNA sequencing experiments are available on Gene Expression Omnibus (GEO) (GSE196928) [https://www.ncbi.nlm.nih.gov/geo/query/acc.cgi?acc=GSE196928]. The R code used for data analysis is available on GitHub at https://github.com/OSU-BMBL/Spinal-cord-scRNAseq with the DOI code on Zenodo: 10.5281/zenodo.6590552 [https://doi.org/10.5281/ZENODO.6590552].

For manuscripts utilizing custom algorithms or software that are central to the research but not yet described in published literature, software must be made available to editors and reviewers. We strongly encourage code deposition in a community repository (e.g. GitHub). See the Nature Portfolio guidelines for submitting code & software for further information.

Data

Policy information about availability of data

All manuscripts must include a data availability statement. This statement should provide the following information, where applicable:

- Accession codes, unique identifiers, or web links for publicly available datasets
- A description of any restrictions on data availability
- For clinical datasets or third party data, please ensure that the statement adheres to our policy

Source data are provided with this paper and deposited to the Open Data Commons for Spinal Cord Injury (ODC-SCI) Accession: 695, DOI:10.34945/F5B012, [https://odc-sci.org/data/695]. Gene expression data for bulk and single cell RNA sequencing experiments are available on Gene Expression Omnibus (GEO) (GSE196928) [https://www.ncbi.nlm.nih.gov/geo/query/acc.cgi?acc=GSE196928].

Field-specific reporting

Please select the one below that is the best fit for your research. If you are not sure, read the appropriate sections before making your selection.

☒ Life sciences ☐ Behavioural & social sciences ☐ Ecological, evolutionary & environmental sciences

For a reference copy of the document with all sections, see nature.com/documents/nr-reporting-summary-flat.pdf

Life sciences study design

All studies must disclose on these points even when the disclosure is negative.

Sample size	Sample sizes were determined a priori from historical data using behavioral measures as the primary outcome, with power (1-β) set to 0.8 and α = 0.05. Bulk and single cell RNA sequencing experiments used groups of mice with sample sizes determined to be sufficient by previous studies (Li et al., 2020).
Data exclusions	No data were excluded from the analysis
Replication	Data in Fig. 1 were successfully reproduced in Fig. 3 and in Sup. Fig. 5, and in two different SCI models in Sup. Fig. 4. Data in Fig. 3 (rCCL2 and TLR2 ag) were successfully reproduced in Sup. Fig. 6. Microglia depletion data in Sup. Figs. 1, 2 and 5 were successfully reproduced twice in quality control experiments for each batch of PLX5622 diet received. Data in Sup. Fig. 2 were successfully reproduced in Sup. Fig. 5. Bulk and single cell RNA sequencing experiments in Fig. 2, 4-8 and Sup. Figs. 7-15 used 3-4 mice per group and were not replicated due to cost and logistics.
Randomization	For all experiments using PLX5622, animals were randomly assigned to cages then cages were randomly assigned to a diet group using QuickCalcs (GraphPad software).
Blinding	To ensure experimenters were blinded to diet groups, mice were acclimated to diet-free cages before testing, and these cages were coded (e.g. 'A', 'B' 'C') prior to testing. Experimenters were also blinded to diet group for histology, tissue processing, image acquisition and data analysis by coding the animals and tissue.

Reporting for specific materials, systems and methods

We require information from authors about some types of materials, experimental systems and methods used in many studies. Here, indicate whether each material, system or method listed is relevant to your study. If you are not sure if a list item applies to your research, read the appropriate section before selecting a response.

Materials & experimental systems

n/a	Involved in the study	
☐	☒	Antibodies
☒	☐	Eukaryotic cell lines
☒	☐	Palaeontology and archaeology
☐	☒	Animals and other organisms
☒	☐	Human research participants
☒	☐	Clinical data
☒	☐	Dual use research of concern

Methods

n/a	Involved in the study	
☒	☐	ChIP-seq
☐	☒	Flow cytometry
☒	☐	MRI-based neuroimaging

Antibodies

Antibodies used	Please see "Key Resources Table" as a supporting document to this paper for a complete list of antibodies used.
Validation	Please refer to the RRID identifier information for antibodies detailed in the "Key Resources Table" for validation and citation information.

March 2021

Animals and other organisms

Policy information about studies involving animals; ARRIVE guidelines recommended for reporting animal research

Laboratory animals	All surgical and postoperative care procedures were performed in accordance with The Ohio State University Institutional Animal Care and Use Committee. Adult female (8-10 week old) female C57BL/6J (WT) mice were purchased from Jackson Laboratories (RRID: ISMR_JAX:000664). Mice were age and weight-matched within experiments. Animals were housed under conventional ventilation conditions on a 12 hour light-dark cycle with *ad libitum* access to food and water. Room temperature was between 20-26ºC and humidity was between 30-70%.
Wild animals	No wild animals were used in this study
Field-collected samples	No field samples were used in this study
Ethics oversight	All surgical and postoperative care procedures were performed in accordance with The Ohio State University Institutional Animal Care and Use Committee.

Note that full information on the approval of the study protocol must also be provided in the manuscript.

Flow Cytometry

Plots

Confirm that:

- [X] The axis labels state the marker and fluorochrome used (e.g. CD4-FITC).
- [X] The axis scales are clearly visible. Include numbers along axes only for bottom left plot of group (a 'group' is an analysis of identical markers).
- [X] All plots are contour plots with outliers or pseudocolor plots.
- [X] A numerical value for number of cells or percentage (with statistics) is provided.

Methodology

Sample preparation	Mice were anesthetized using 1.5x the surgical dose of anesthetic.<br>**Blood**: Blood was collected via cardiac puncture with a 25G syringe and transferred to blood collcetion tubes coated with EDTA. A 50 A 50 µl sample of whole blood per mouse was used for flow cytometry.<br>**Spleen**: The spleen was rapidly dissected, weighed, and placed in a small volume of DMEM. Spleens were minced with sterile dissection scissors and mashed through a 40 µm sterile cell filter using the plunger of a 3 ml syringe and rinsed with 10 ml of IMDM.<br>**Bone marrow (BM)**: Both femurs from each mouse were removed, cleaned, and placed in a small volume of DMEM. Pictures of representative bones were captured using an iPhone 6s and pseudocolored in ImageJ. Bone marrow cells were isolated by flushing bones with 10 ml of DMEM through a 40 µm sterile cell filter.<br>**Sample processing**: Samples were processed as described previously74. Briefly, blood, BM and spleen samples were diluted 1:5 with NH4Cl red blood cell lysis buffer (StemCell Technologies, # 7850) and incubated for 5 mins at RT. Cells were centrifuged (300 x g for 4 mins) and resuspended in 0.1M PBS. Cells were then incubated with 1:100 zombie green viability dye (BioLegend, 423112) for the exclusion of dead cells. Cells were washed then resuspended in flow buffer (0.1M PBS with 2% FBS) containing rat anti-CD16/32 (1:200; BD Bioscience) for 10 mins on ice to block Fc receptors. Cells were then incubated with flow cytometry antibodies (see Key Resources Table) for 30 mins on ice, washed and resuspended in flow buffer. 10 µl of liquid counting beads (BD Biosciences, 335925) were added to allow for quantification of absolute cell numbers. Samples were processed on a BD Fortessa flow cytometer (BD Biosciences) running FACS Diva software (v9.0) (BD) and analyzed using FlowJo (v10.8.1) (BD). OneComp ebeads (ThermoFisher, #01-1111-41) were used to set voltage intensities and compensation thresholds to remove spectral overlap. Unstained controls, isotype controls, and fluorescence minus one controls were used to identify background staining levels and determine gate placement. Doublets were excluded based on linearity of FSC-A and FSC-H. From singlets, live cells were identified as the Zombie-FITClo/- population. Neutrophils were designated Ly6G+Ly6C+ cells and monocytes were designated Ly6G-Ly6C+ cells. CD11b and CD11c staining was also used to confirm cell identity.
Instrument	BD Fortessa flow cytometer (BD Biosciences)
Software	FACS Diva software (v9.0) (BD) and FlowJo (v10.8.1) (BD).
Cell population abundance	10 µl of liquid counting beads (BD Biosciences, 335925) were added to allow for quantification of absolute cell numbers.
Gating strategy	Unstained controls, isotype controls, and fluorescence minus one controls were used to identify background staining levels and determine gate placement. Doublets were excluded based on linearity of FSC-A and FSC-H. From singlets, live cells were identified as the Zombie-FITClo/- population. Neutrophils were designated Ly6G+Ly6C+ cells and monocytes were designated Ly6G-Ly6C+ cells. CD11b and CD11c staining was also used to confirm cell identity.

- [X] Tick this box to confirm that a figure exemplifying the gating strategy is provided in the Supplementary Information.
